# Azo-Based Iridium(III) Complexes as Multicolor Phosphorescent Probes to Detect Hypoxia in 3D Multicellular Tumor Spheroids

**DOI:** 10.1038/srep14837

**Published:** 2015-10-01

**Authors:** Lingli Sun, Guanying Li, Xiang Chen, Yu Chen, Chengzhi Jin, Liangnian Ji, Hui Chao

**Affiliations:** 1 MOE Key Laboratory of Bioinorganic and Synthetic Chemistry, School of Chemistry and Chemical Engineering, Sun Yat-Sen University, Guangzhou, 510275, China

## Abstract

Hypoxia is an important characteristic of malignant solid tumors and is considered as a possible causative factor for serious resistance to chemo- and radiotherapy. The exploration of novel fluorescent probes capable of detecting hypoxia in solid tumors will aid tumor diagnosis and treatment. In this study, we reported the design and synthesis of a series of “off-on” phosphorescence probes for hypoxia detection in adherent and three-dimensional multicellular spheroid models. All of the iridium(III) complexes incorporate an azo group as an azo-reductase reactive moiety to detect hypoxia. Reduction of non-phosphorescent probes **Ir1**-**Ir8** by reductases under hypoxic conditions resulted in the generation of highly phosphorescent corresponding amines for detection of hypoxic regions. Moreover, these probes can penetrate into 3D multicellular spheroids over 100 μm and image the hypoxic regions. Most importantly, these probes display a high selectivity for the detection of hypoxia in 2D cells and 3D multicellular spheroids.

Hypoxia is caused by the limited delivery of oxygen distant from functional blood vessels (>100 μm)^1^ and is a typical characteristic of solid tumors. Cells located in hypoxic areas often become quiescent, limiting the effects of anti-cancer drugs^2^. The hypoxic status has been considered an indicator of an adverse prognosis for solid tumors because it indicates tumor progression toward a more malignant phenotype with increased metastatic potential and resistance to treatment^3^,^4^,^5^,^6^. Therefore, the development of novel methods for the detection of hypoxic regions in a solid tumor is important. To date, many approaches have been used to selectively detect hypoxic regions in solid tumors^7^,^8^,^9^,^10^,^11^,^12^,^13^. Among these approaches, the fluorescence imaging method offers various advantages, including simple operation and high sensitivity.

Many small molecular-based probes for the detection of hypoxia exploit the phenomenon that hypoxia causes an increase in reductive stress. For example, some reductases such as nitro-reductase (NTR), quinone-reductase (QR) and azo-reductase (AzoR) are highly expressed in hypoxic tumors^14^. Taken advantages of these facts, many NTR-sensitive hypoxia probes utilizing nitro (or p-nitrobenzyl) moiety as NTR substrates have successfully developed^15^,^16^,^17^,^18^,^19^,^20^. Quinone-based hypoxia probes have been also reported^21^,^22^,^23^. In addition, AzoR is an important family of reductases that can reduce the azo bond in a stepwise manner to anilines^24^,^25^. The catalysis involves the sequential transfer of four electrons to form aniline after the final reduction; in the first step of reduction, the formation of azo-anion radical compounds is a reversible oxygen-dependent process (Fig. 1a). In normoxic environment, which is abundant in oxygen, the reduction is suppressed. Moreover, azo dyes, such as azobenzene derivatives, are non-luminescent because of the ultrafast conformational change around the N = N bond after photoexcitation^26^,^27^,^28^,^29^. Conjugating an azo moiety directly to a fluorophore can quench the original fluorescence. Exploiting these characteristics, certain azo compounds have been proposed in AzoR detection in bacteria^24^,^30^ and tumor-targeted therapy prodrugs^31^,^32^,^33^. Recently, some azo-based compounds showed chemical reactivity with biological reducing agents. Li *et al.* have reported an azo-based fluorescent probe for sulfide, which would react with sulfide and be reduced to amino produce^34^. Authors suggested that the electron deficiency of the azobenzene group was crucial to the reaction between the azo-based probe and sulfide. Our group has also synthesized some dinuclear metal complexes containing azo linker and found they could react with thiols or sulfite/bisulfite ions^35^,^36^,^37^. The metal coordination is also considerable electron deficient for azo group. A few examples have reported employing azo-based turn-on fluorescent probes to obtain images of the hypoxic level *in vitro* or *in vivo*^25^,^38^,^39^.

One limitation of these reductase-based fluorescent probes for the detection of AzoR or hypoxia was that they were evaluated with adherent cells in which the hypoxia phenomenon was artificially induced, such as through the presence of a glass cover on top of the cells^39^, the use of a drug treatment to abolish cardiac contractility^40^ or vessel ligation^25^. These models do not adequately reflect the microenvironment of cells *in vivo*. A three-dimensional (3D) model, such as multicellular spheroids, organotypic explants and 3D scaffolds, can overcome these problems because it more closely reflects the dimensionality of the tumor microenvironment, thus rendering results with a higher similarity to those in an *in vivo* tumor^41^. In particular, 3D multicellular spheroids are advantageous *in vitro* models for providing cell-cell and cell-matrix interactions and recreating certain mass transport limitations likely encountered *in vivo*^42^,^43^.

One valuable characteristic of these models is their diffusion limit of approximately 150–200 μm for many molecules, particularly oxygen^43^,^44^. Because of these mass transport limitations, spheroids with diameters larger than 400–500 μm generally display gradients of oxygen and form a hypoxic core in the center. Thus, 3D multicellular spheroids are excellent models for detecting hypoxia by generating a hypoxic environment without specialized hypoxic chambers. To date, only the Papkovsky group^45^,^46^ has evaluated oxygenation imaging in 3D multicellular spheroids using luminescent probes based on oxygen quenching of luminescence. To image hypoxia using bioreductase-reactive compounds is of high value to distinguish the long-term hypoxia in solid tumors with acute hypoxia. By far, using reductase-reactive luminescent probes to image hypoxia in 3D multicellular spheroids is still blank.

As described, we have successfully developed some azo-based dinuclear iridium(III) complexes as phosphorescent probes for thiols or sulfite/bisulfite ions^35^,^36^,^37^. We speculate that electron efficient groups would reduce the chemical reactivity between azo-group and biological reducing agents, and facilitate the reaction selectivity of azo compounds with AzoR. In this study, we presented a series of mononuclear Ir(III) complexes containing azo group as the bio-reducible moiety and phosphorescent trigger. Complexes [Ir(C^N)_2_(N^N)]^+^(**Ir1**-**Ir8**, Fig. 1b) were synthesized, and their capability of being reduced under hypoxic conditions was tested with fresh rat liver microsomes. Hypoxia imaging in cells was performed in HeLa cells cultured with different oxygen concentrations. More importantly, we employed these probes for hypoxia imaging in 3D multicellular spheroids, which display a hypoxic region in the center, closely reflecting *in vivo* solid tumors. Different sizes of 3D multicellular spheroids were cultured and imaged with these probes to correlate the hypoxic region within the 3D multicellular spheroids with their sizes.

## Results

### Synthesis and characterization

The p-azobpy and dmap-azobpy ligands were prepared by the reduction of nitro precursors. The preparations of the **Ir1-Ir8** complexes were performed through bridge splitting reactions of the dinuclear precursors [Ir(C^N)_2_Cl]_2_ with the N^N ligands p-azobpy and dmap-azobpy in a stoichiometric amount. The formations of all of the complexes were further confirmed using FT-IR, ESI-MS, ^1^H NMR and ^13^C NMR spectroscopy (Figures S1–S24).

**Figure 2:**
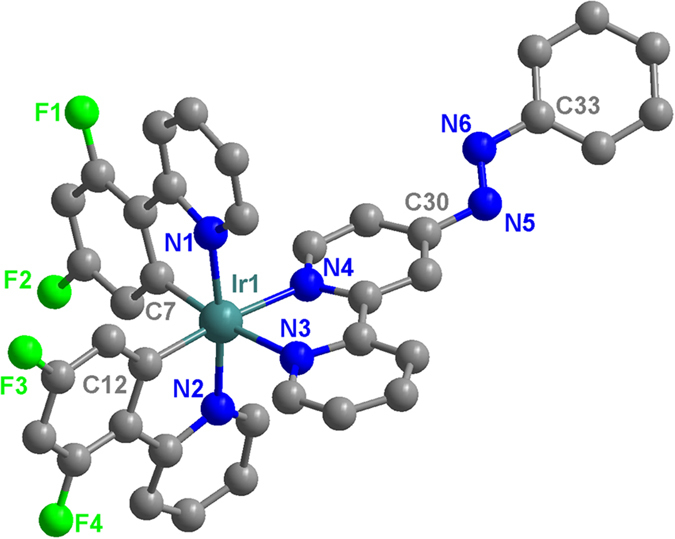
The crystal structure of **Ir1**. For clarity, the solvent molecules, the hydrogen atoms, and the counter anions are omitted.

The single crystal structure of **Ir1** was analyzed through single-crystal X-ray diffraction, and the crystal structure is shown in Fig. 2. Crystal data and structural refinements are shown in Table S1. Selected bond lengths and angles are presented in Table S2. The coordination of the iridium atom is a distorted octahedral. The cyclometalated C atoms of the dfppy ligands are in a mutual cis arrangement, and their high trans influence leads to Ir-N (p-azobpy) distances of 2.125(9) Å and 2.111(9) Å, which were longer than the corresponding distances of Ir-N (dfppy) (2.061(9) Å and 2.049(9) Å) in the cyclometalated ligand. This result was similar to the reported crystal structures of cyclometalated Ir(III) complexes [Ir(C^N)_2_(N^N)]^+^ 47,48.

### Electrochemical studies

We evaluated the reduction potential of these probes using cyclic voltammetry, and summarized the redox potentials of the azo group and the iridium centre of these probes in Table 1. Cyclic voltammograms of the probes in an anaerobic CH_3_CN solution displayed two reduction waves corresponding to consecutive one-electron reductions. The first reduction waves (corresponding to azo^0/−1^) were reversible, while the second waves (corresponding to azo^−1/−2^) were not completely reversible. The φ (azo^0/−1^) of these probes varied from −0.6748 to −0.8930 V vs SCE, while (azo^−1/−2^) ranged from −0.8442 to −1.1233 V vs SCE. The redox potential of the azo group in the non-substituted aromatic azo compounds (R = H, **Ir1-Ir4**) was less than that in the dimethylamine substituted compounds (R = NMe_2_, **Ir5-Ir8**), which is an electron-donating group. More importantly, compared with the free organic azo compounds, such as methyl red (φ = −1.2665 V) and disperse red (φ = −1.1295 V)^25^, the reduction potential of the azo group in these azo compounds decreased. We reasoned that the azo group is activated when conjugated to iridium(III) complexes. Thus, these azo-containing Ir(III) compounds appeared to be good candidates for use as a hypoxic reduction moiety and performed even more efficiently than organic azo compounds.

### Hypoxia response assays

**Figure 3:**
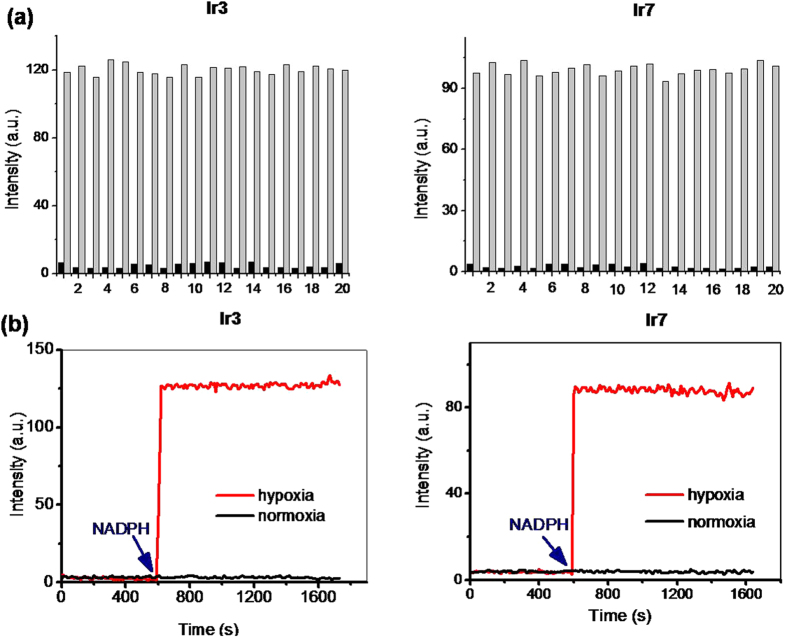
(**a**) Phosphorescence intensity of **Ir3** and **Ir7** (2.5 μM) treated with different amino acids or ions (1 mM of each species) in a mixed solution of 0.1 M potassium phosphate buffer (pH 7.4) under normoxic and hypoxic environments. Bars on the left represent the luminescent responses under a normoxic environment, while bars on the right represent the subsequent degassing with argon gas and addition of rat liver microsomes (0.25 mg protein/mL) and 50 μM NADPH for 30 min at 37 °C. From bar (1) to (20): Cys, cystine, GSH, sodium ascorbate, H_2_O_2_, Mg^2+^, Cu^2+^, Zn^2+^, Co^2+^, Ca^2+^, SO_4_^2**−**^, HCO_3_^−^, NO_2_^−^, NO_3_^−^, SO_3_^2−^, I^−^, ClO_4_^−^, ClO^−^, S^2−^, CO_3_^2−^. λ_ex/em_ = 405/580 nm; (**b**) Time-dependent changes of phosphorescence intensity of **Ir3** (left) and **Ir7** (right) (2.5 μM) under normoxic and hypoxic conditions. Data were measured at 37 °C in 0.1 M potassium phosphate buffer (pH 7.4) containing rat liver microsomes and NADPH as a cofactor. NADPH was added at the point indicated by the arrow. λ_ex/em_ = 405/580 nm.

The azo-based probes were non-emissive in organic solution and in aqueous PBS solution. We examined whether other ions could lead to a notable phosphorescent response toward these probes (Fig. 3a and S25). A high concentration of ions (1 mM of H_2_O_2_, Mg^2+^, Cu^2+^, Zn^2+^, Co^2+^, Ca^2+^, SO_4_^2−^, HCO_3_^−^, NO_2_^−^, NO_3_^−^, SO_3_^2−^, I^−^, ClO_4_^−^, ClO^−^, S^2−^, CO_3_^2−^) was added to a PBS solution of probes and incubated for 30 min at 37 °C. Among the additional tested ions, limited responses in phosphorescence intensity were observed. Considering that the azo group might be reduced inside of the cells, potential biological reducing agents, such as cysteine (Cys), cystine, sodium ascorbate, and reduced glutathione (GSH), were also tested. No enhancement in phosphorescence intensity was observed. This result suggests that these azo-based Ir(III) complexes are chemically inert toward common ions and bio-related reducing agents.

**Figure 4:**
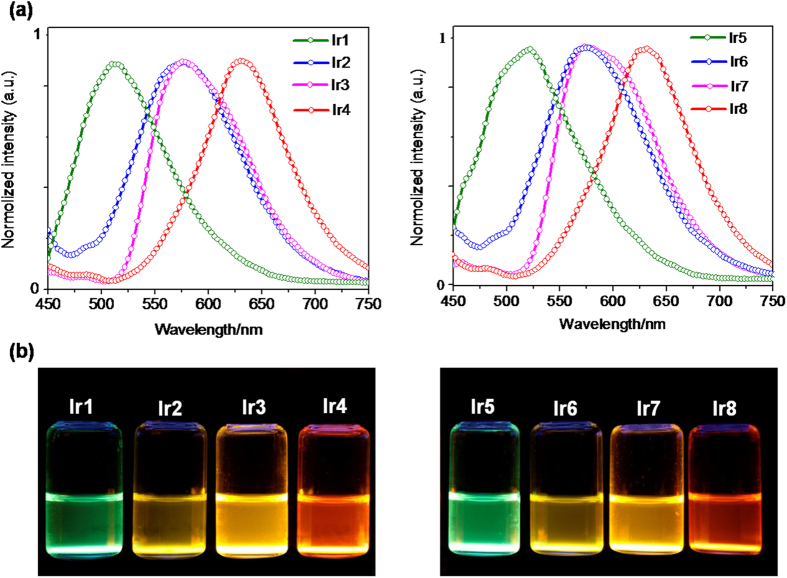
(**a**) Emission spectra of probes incubated with rat liver microsomes and 50 μM NADPH at 37** **°C for 30 min. (**b**) Photos of probes **Ir1**-**Ir8** after incubation with rat liver microsomes and NADPH were captured under a handle UV lamp (365  nm).

Next, we tested the capability of these probes to detect hypoxia in biological systems using rat liver microsomes, which contain various reductases. Dissolved oxygen was removed from a PBS solution of probes by argon bubbling, and then fresh microsomes from rat liver were added. When NADPH as a cofactor for the reductases was added into this mixture, the phosphorescence intensity increased sharply to maximum within 30 s and then remained constant (Fig. 3b and S26), suggesting that these probes were reduced rapidly by reductases in liver microsomes. More importantly, this enhancement in phosphorescence intensity was only observed under hypoxic conditions. When reduced under hypoxia, these probes exhibited enhancements in phosphorescence intensity of 17.5-fold for **Ir1**, 16.9-fold for **Ir2**, 39.0-fold for **Ir3**, 38.4-fold for **Ir4**, 11.4-fold for **Ir5**, 13.5-fold for **Ir6**, 58.8-fold for **Ir7**, and 54.2-fold for **Ir8**, respectively. After reduction under hypoxia, the maximum emission intensity ranged from 510 nm for **Ir1** and **Ir5** to 640 nm for **Ir4** and **Ir8**, displaying a green to red emissive solution, which could be observed by the naked eye (Fig. 4). Interestingly, the phosphorescent response to hypoxia could also be obtained in a mixed solution containing a high concentration of ions or bio-related reducing agents (Fig. 3a), indicating that these probes could also function in a complex biological background. Additionally, the phosphorescence intensities of these reduction products were only slightly affected by pH when the pH values were varied from 3.0 to 10.0. (Figure S27) Thus, the azo-based Ir(III) complexes **Ir1-Ir8** could be selective turn-on phosphorescent probes for hypoxia with a multi-emissive color.

**Figure 5:**
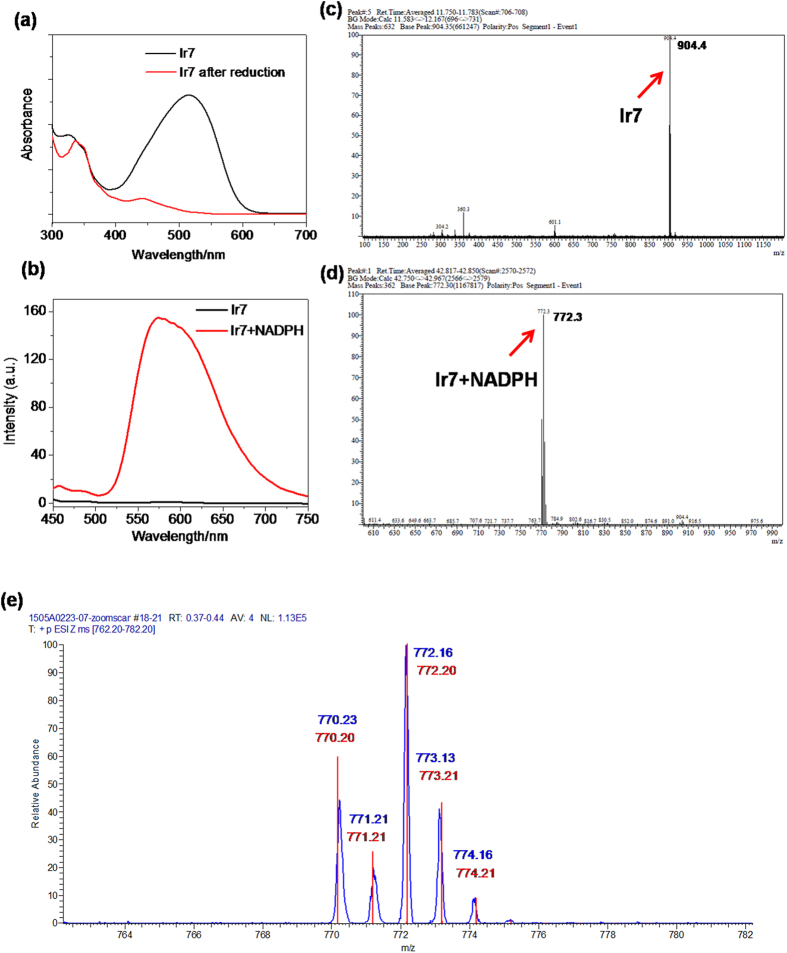
UV-Vis (a) and emission (b) spectral changes of **Ir7** in the presence of rat liver microsomes (0.25 mg protein/mL)/NADPH (50 μM) under normoxic (black) or hypoxic (red) conditions. Excitation wavelength: 405 nm. Both ex/em slits were set to 10 nm. (**c–e**): ESI-MS analysis of the reaction mixture generated by **Ir7**; (**c**) ESI-MS spectrum of **Ir7**. (**d**) ESI-MS spectrum of the product of **Ir7** after incubation with rat liver microsomes/NADPH at 37 °C for 30 min. (**e**) Zoom scan MS spectra of iridium isotope peaks of (**d**) and the simulation of **IrNH**_**2**_**3**.

We speculated that these azo compounds were completely reduced in the corresponding amine production (**IrNH**_**2**_**1**-**IrNH**_**2**_**4**) under hypoxic conditions, which were strongly phosphorescent emissive. The absorption spectra also demonstrated the reduction of the azo bond under hypoxia. For example, **Ir7** displayed a broad absorption peak at 510 nm (Fig. 5A), attributed to an electron transition of the azo bond. However, after reduction using liver microsomes under hypoxic conditions, this peak disappeared. We further confirmed the production of amines generated from **Ir7** reduction by liver microsomes under hypoxia using ESI-MS analysis (Fig. 5c–e). The mass spectrum of **Ir7** showed a peak at 904.4, corresponding to [M-Cl]^+^. After incubation with rat liver microsomes/NADPH under hypoxia, the reduced product was found, as shown by the appearance of the peak at 772.3. Zoom scan of the iridium isotope peaks was also conducted and the result was perfectly fit to the simulation of the amino reduction product (**IrNH**_**2**_**3**). Similar results were observed in other probes (Figures S28–S34).

### Imaging hypoxia in adherent cells

**Figure 6:**
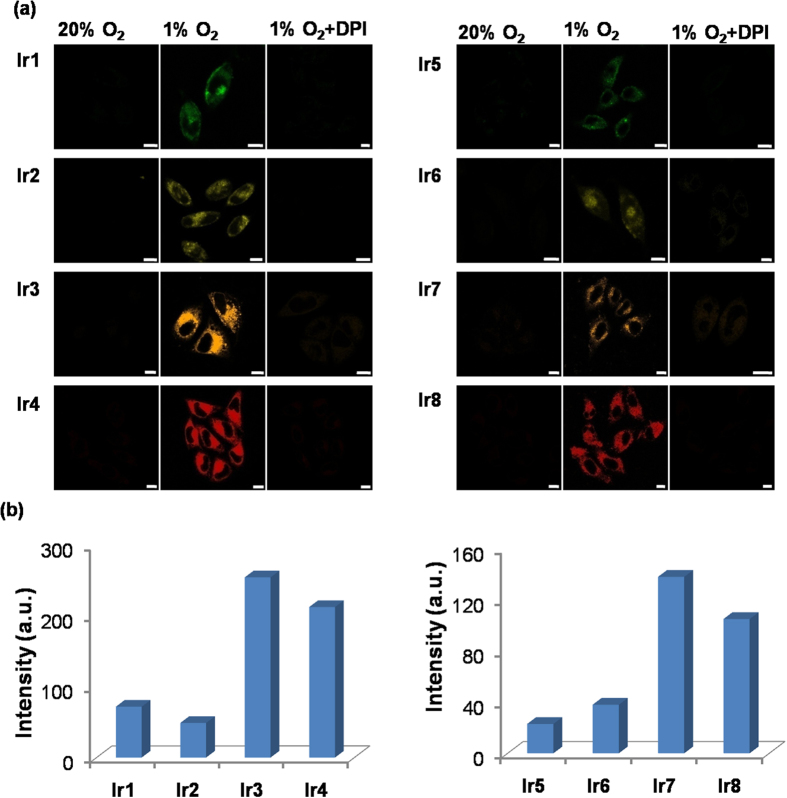
(**a**) Confocal images of adherent HeLa cells treated with probes under normoxia (20% O_2_) and hypoxia (1% O_2_) with or without DPI (100 μM). HeLa cells were incubated with compounds (2.5 μM) for 30 min. **Ir1**, **Ir5**: λ_ex/em_ = 405/510 ± 20 nm; **Ir2**, **Ir6**: λ_ex/em_ = 405/570 ± 20 nm; **Ir3**, **Ir7**: λ_em_ = 405/580 ± 20 nm; **Ir4**, **Ir8**: λ_em_ = 405/640 ± 20 nm; Scale bar: 10 μm. (**b**) Quantified phosphorescence intensities of HeLa cells incubated with probes **Ir1**-**Ir8** under hypoxia (1% O_2_).

We applied the probes to investigate whether they could detect hypoxia in living cells. As shown in Fig. 6a, when the probes were treated with HeLa cells under normoxia, no phosphorescent signal could be detected in the cells. However, when the cells were cultured under hypoxia (1% O_2_), the addition of the probes induced a remarkable increase in the phosphorescence intensity within the cells. Flavoproteins, such as NADPH-cytochrome P450 reductase, are thought to be responsible for the metabolic activation of bioreductive compounds under hypoxia^6^. When a flavoprotein inhibitor diphenyliodonium chloride (DPI)^49^,^50^ was added to the hypoxic cultured cells, the phosphorescence intensity was dramatically suppressed, suggesting that these probes are possibly reduced by flavoproteins. For all of these probes, the complexes with an unsubstituted aromatic azo ligand (**Ir1-Ir4**) displayed stronger phosphorescence intensity than those with dimethylamino group-substituted aromatic azo ligands (**Ir5-Ir8**) when they were reduced under hypoxia (Fig. 6b). Among the complexes, **Ir3** exhibited the strongest phosphorescence intensity. Additionally, these probes displayed different emissive colors when reduced under hypoxia in solution, as well as in cells, expanding the practical application in living cell imaging for hypoxia.

### Imaging of hypoxia in 3D multicellular spheroids

Inspired by the selective response in phosphorescence intensity toward hypoxia of these probes *in vitro* assays and in imaging of adherent cells, we sought to establish the ability of these probes to detect the hypoxic level in 3D multicellular spheroids, which are more appropriate models of a solid tumor offering more similar microenvironments to those of tumors than adherent tumor cells. Nutrients, particularly oxygen, only diffuse 50–100 μm into tumor spheroids, resulting in hypoxic regions of solid tumors farther than 100 μm from the blood vessels^44^,^51^. Therefore, 3D multicellular tumor spheroid cultures develop oxygen gradients at diameters between 200 μm and 500 μm due to the oxygen diffusion limitation.

**Figure 7:**
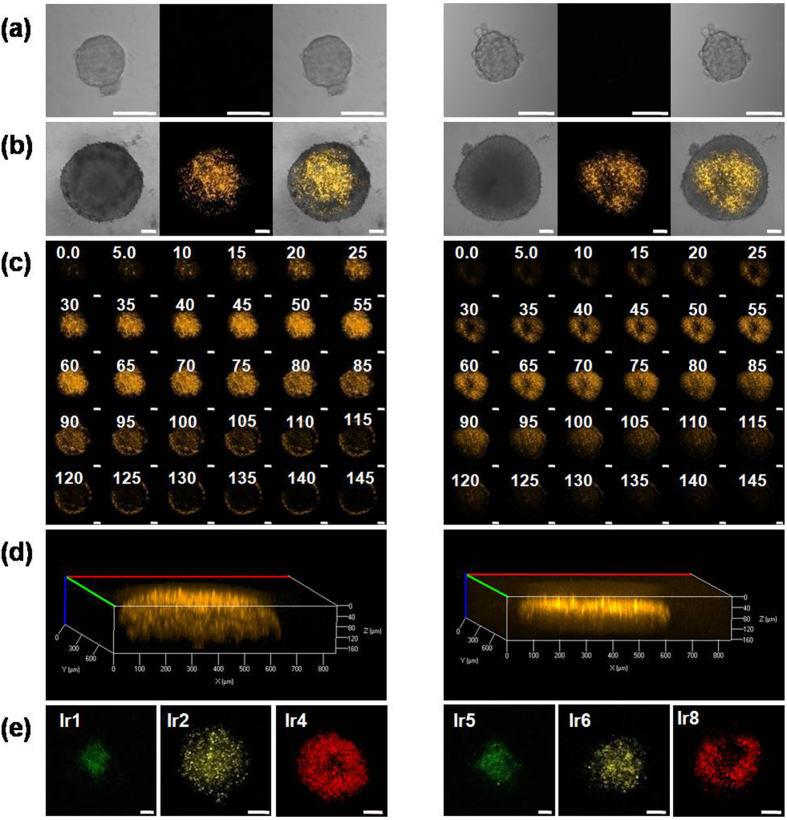
(**a–d**) Confocal images of 3D multicellular spheroids incubated with **Ir3** (left) and **Ir7** (right). λ_ex/em_ = 405/580 ± 20 nm; (**a**) small 3D spheroids (diameter of 100–150 μm); (**b**) large 3D spheroids (diameter of 400–500 μm); (**c**) Scanned Z-axis for large 3D spheroids at 5 μm intervals. Scale bar: 100 μm. (**d**) The merged images of Z-stack images of an intact spheroid. (**e**) Confocal images of 3D multicellular spheroids incubated with **Ir1, Ir2** and **Ir4** (left) and **Ir5, Ir6** and **Ir8** (right). λ_ex_ = 405 nm, **Ir1**, **Ir5:** λ_em_ = 510 ± 20 nm; **Ir2**, **Ir6:** λ_em_ = 570 ± 20 nm; **Ir3**, **Ir7:** λ_em_ = 580 ± 20 nm; **Ir4**, **Ir8:** λ_em_ = 640 ± 20 nm. The images were obtained under a 10 × objective. Scale bars represent 100 μm.

Spheroids with different sizes (approximately 150 μm and 500 μm in diameter) were cultured and exposed to these probes (2.5 μM), and then the phosphorescence was examined by confocal microscopy. (Fig. 7 and S35-S37) As shown in Fig. 7, no phosphorescence intensity could be detected in the spheroids of a small size because they are not sufficiently large to provide an efficient hypoxic region. In contrast, in large spheroids, remarkable phosphorescence intensity was observed in the center of the spheroids with a distance of approximately 100 μm from the outer edge, suggesting a hypoxic region in the spheroids. The phosphorescent images along the z-axis were also captured every 5 μm. The spheroids exhibited a substantially stronger phosphorescence intensity at depths of 40–80 μm, which could be considered the center region of these spheroids. This result indicates that these 3D multicellular spheroids have hypoxic regions in the center, as revealed by imaging of the hypoxic sensitive probes.

### Cell viability assay

**Figure 8:**
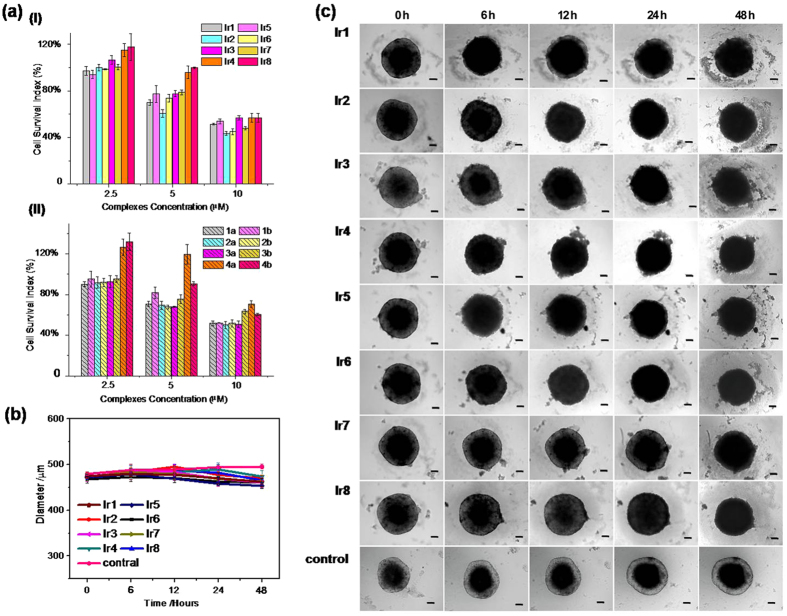
(**a**) Cell viability of adherent 2D HeLa cells incubated with different concentrations of probes for 12 h under normoxic (I) and hypoxic (II) environment. (**b**) Growth inhibition assay in 3D spheroids. Growth curves of spheroids after various treatments. (**c**) Representative images of spheroids treated with eight probes (2.5 μM). Spheroids cultured in DMEM as a control; Scale bars = 50 μm.

An ideal cellular probe for practical applications should minimally perturb living systems at the concentrations used. To verify the potential toxicity of **Ir1-Ir8** to cells, MTT assays^52^ were conducted under normoxic and hypoxic conditions (Fig. 8a). The complexes were exposed to HeLa cells with different concentrations (2.5 μM, 5 μM, and 10 μM) for 12 h, and then the cell viability was evaluated by an MTT assay. The results for the normoxic and hypoxic conditions were similar for all of the compounds tested. Generally, at probe concentrations of 2.5 μM, HeLa cells exhibited viability higher than 80% after incubation for 12 h. These results suggested that under our experimental conditions (2.5 μM for 0.5 h incubation), these probes exhibited only a slight cytotoxic effect against HeLa cells.

The inhibition of these probes against 3D multicellular spheroids was also evaluated^53^. 3D multicellular spheroids were grown until the diameters were larger than 400 μm. Then, the probes were added, the spheroids were cultured, and images were obtained by internal hours. As shown in Fig. 8b,c, spheroids without the treatment of probes grew slowly, as the diameter of the spheroids increased with time. Cells located in the spheroid periphery were relatively free moving while the innermost cells became compact. The loose region with a depth of approximately 100 μm is similar to that observed for the diffusion of oxygen^44^. The reason for this phenomenon may be due to the oxygen transport limitations in spheroids. When spheroids were treated with probes, their diameters initially increased and then remained unchanged after 1 day of culture. These results indicated that these compounds exhibited an inhibition effect against tumor spheroids after 1 day but only a slight toxic effect over a short incubation time (6 h in the imaging experiments).

## Discussion

Hypoxia is an important characteristic of malignant solid tumors and is considered a possible causative factor for serious resistance to chemo- and radiotherapy. The exploration of novel fluorescent probes capable of detecting hypoxia in solid tumors will aid tumor diagnosis and treatment. Previous work has revealed that electron deficiency of azo group will accelerate the chemical reactivity of azo compounds with reducing agents. To avoid the chemical reduction of azo group, we designed a series of azo-based Ir(III) complexes, in which **Ir1**-**Ir4** are non-substituted azobenzene derivatives (R = H), while **Ir5**-**Ir8** contain electron-donating dimethyl amino group (R = N(CH_3_)_2_). All these Ir(III) complexes are non-phosphorescent, because of azo bond quenching effect. As expected, they could not be reduced by chemical reducing agents, such as thiols, sodium ascorbate, sulfide or sulfite, but are capable of being reduced by endogenous bioreductases under hypoxia. The capability of being reduced by bioreductases is related with the first redox potentials of azo bond (φ (azo^0/−1^)), in which terms it is less negative in that R = H than that R = N(CH_3_)_2_, and it follows the order of **Ir1** > **Ir3** > **Ir4** > **Ir2**, and **Ir5** > **Ir7** > **Ir6** > **Ir8**. However, **Ir1** shows the weakest phosphorescence intensity after biological reduction. We speculate that axial ligands of **Ir1**, as well as **Ir5**, are di-fluorine containing, which provide rich hydrogen bonding environment and make **Ir1** and **Ir5** accessible to other proteins. On another side, the corresponding reduced amino products (**IrNH**_**2**_**1**-**IrNH**_**2**_**4)** are highly phosphorescent (quantum yields and lifetimes are listed in table S3. Except for **IrNH**_**2**_**1**, **IrNH**_**2**_**3** exhibits largest quantum yield value of 0.142 in aqueous solution. These facts could explain why **Ir3** and **Ir7** exhibit much stronger phosphorescence intensity than others.

T. Nagano and K. Hanaoka have developed azo-based turn-on fluorescent probes to obtain images of the hypoxic level in hypoxic cells, branch retinal artery occlusion (BRAO) and ligated mice^25^,^39^, yet not in a 3D multicellular spheroids model. 3D multicellular spheroids model is more reliable than hypoxic cultured adherent cells to study the hypoxia, by generating a hypoxic environment without specialized hypoxic chambers and providing cell-cell, cell-matrix interaction. Because of the inadequate supply of oxygen and nutrients, 3D multicellular spheroids exhibit diffusive gradients of DNA breaks, ATP distribution and enzyme activity, besides oxygen gradient^42^. To image hypoxia using bioreductase-reactive compounds is of high value to distinguish the long-term hypoxia of solid tumor with acute hypoxia. In the presented work, we employ cyclometalated iridium(III) complexes as phosphores, which posses advantages over organic fluorescent probes, such as evident Stokes shift, strong anti-photobleaching, tunable phosphorescence and relative long phosphorescent lifetime^54^. By changing the chemical structures of the axial C^N ligands, the turn-on emissive colors of the complexes can be tuned from green to red, showing multicolor phosphorescent probes for hypoxia and expanding hypoxia imaging application. Remarkable performance of the complexes was exhibited for the detection of a hypoxia environment not only in adherent living cells but also in oxygen-gradient 3D multicellular spheroids at depths of over 100 μm, which have many similarities to the *in vivo* environment. Thus, the probes **Ir1**-**Ir8** are practical and useful tools for studying hypoxia in solid tumors, which is crucial for guiding the subsequent therapeutic decisions, as well as for investigating a variety of hypoxia-related biological phenomena. Further work on the long phosphorescent lifetime complexes for phosphorescence lifetime imaging (PLIM) of hypoxia is ongoing in our lab.

## Methods

### General Procedure

All of the reagents were purchased from commercial sources and used without further purification unless otherwise specified. All of the buffer components were of biological grade and were used as received. All experiments were done according to institutional ethical guidelines on animal care, and all experimental protocols received prior approval by the Animal Care and Use Committee of Sun Yat-Sen University. IrCl_3_, 2-(2,4-difluorophenyl)pyridine (**dfppy**), 2-phenylpyridine (**ppy**), 2-phenylquinoline (**2-pq**), 2,2′-bipyridin-4-amine (**bpy-NH**_**2**_) 3-(4,5-dimethylthiazol-2-yl)-2,5-diphenyltetrazolium bromide (**MTT**) and diphenyliodonium chloride (**DPI**) were purchased from Sigma Aldrich and were used without further purification. Twice-distilled water was used throughout all of the experiments. The ligandsdibenzo[*f,h*]quinoxaline (**dbq**)^55^, 2,2′-bipyridin-4-amine (**bpy-NH**_**2**_)^56^, cyclometalated Ir(III) chloro-bridged dimer [Ir(C^N)_2_Cl]_2_ (C^N = **dfppy**, **ppy**, **2-pq** or **dbq**)^57^, [Ir(ppy)_2_(bpy-NH_2_)]Cl (**IrNH**_**2**_**2**) and [Ir(2-pq)_2_(bpy-NH_2_)]Cl (**IrNH**_**2**_**3**)^58^ were synthesized according to literature methods. Electrospray ionization mass spectra (ESI-MS) were recorded on a LCQ system (Finnigan MAT, USA) in positive mode. The ^1^H NMR spectra were recorded on a Mercury-Plus 500 spectrometer (500 MHz), and ^13^C NMR spectra were recorded on a 500 MHz Superconducting Fourier Transform Nuclear Magnetic Resonance Spectrometer (Varian, INOVA500NB). All of the chemical shifts are reported relative to tetramethylsilane (TMS). Electronic absorption spectra were recorded using a Perkin-Elmer Lambda850 UV/Vis spectrometer. Emission spectra were recorded on a Perkin-Elmer LS 55 luminescence spectrometer at room temperature. The Cyclic voltammetry measurements were performed on a CHI 660A electrochemical workstation. All samples were dissolved in CH_3_CN and were purged with Ar; in addition, 0.1 M tetrabutylammonium perchlorate (TBAP) was used as a supporting electrolyte. A standard three-electrode system composed of a glassy carbon working electrode, a Pt-wire auxiliary electrode and a saturated calomel reference electrode (SCE) was used. The scan rate was 0.1 V·s^−1^. The IR spectra were recorded on an EQUINOX 55 Fourier transformation infra-red spectrometer (Bruker, German) in KBr pellets over a range of 400–4000 cm^−1^. The pH measurements were obtained with a Sartorius PB-10 pH meter.

### Synthesis of 4-(phenyldiazenyl)-2,2′-bipyridine (p-azobpy)

Pyridine (3 mL) and 5 mL of 50% NaOH were heated with 2,2′-bipyridine-4-amine (1.0 mmol) to 80 °C. Isonitrosobenzene (1.5 mmol) was added over a 30 min period. The reaction mixture was stirred overnight and then cooled at room temperature. The solvent was evaporated to dryness under reduced pressure. The crude mixture was then purified by column chromatography on silica using CHCl_3_/CH_3_OH (v/v, 50:1) as eluent to obtain the product as a claybank solid (466.3 mg, 89.7%). ^1^H NMR (500 MHz, DMSO-d_6_) δ 8.93 (d, *J* = 5.5 Hz, 1H), 8.74 (d, *J* = 4.5 Hz, 1H), 8.69 (s, 1H), 8.46 (d, *J* = 8.0 Hz, 1H), 8.00 (ddd, *J* = 9.5, 4.5, 3.0 Hz, 3H), 7.84 (dd, *J* = 5.0, 2.0 Hz, 1H), 7.70–7.62 (m, 4H), 7.54–7.46 (m, 1H). ^13^C NMR (126 MHz, DMSO-d_6_) δ 158.2, 157.9, 155.0, 152.3, 151.8, 149.9, 137.9, 133.4, 130.2, 129.0, 125.2, 123.7, 121.2, 118.4, 117.6, 112.1. FT-IR (KBr pellet, cm^−1^): *ν*(N = N) 1448.0, m. ESI-MS (*m/z*): [M+H]^+^ calculated for C_16_H_12_N_4_, 261.1; found, 261.1.

### Synthesis of 4-(2,2′-bipyridin-4-yldiazenyl)-N,N-dimethylaniline (dmap-azobpy)

NaNO_2_ (2.1 mmol) and 2, 2′-bipyridin-4-amine (2.0 mmol) were dissolved in water (10 mL). The mixture was then cooled to 2 °C. Cold concentrated HCl (800 μL) was added slowly to ensure that the temperature was below 5 °C. The mixture was stirred for another 10 min and then added dropwise into a dimethylaniline/acetic acid/water (0.2 mL/1 mL/20 mL) solution incubated in an ice bath. A bright red precipitate was generated immediately. After this addition, the mixture was stirred overnight, and the precipitate was collected by filtering, washing with cold water and then drying under reduced pressure obtain the product as a dark red solid (576.2 mg, 95.1%). ^1^H NMR (500 MHz, CDCl_3_) δ 8.81 (d, *J* = 5.5 Hz, 1H), 8.78 (d, *J* = 1.5 Hz, 1H), 8.76 (d, *J* = 5.0 Hz, 1H), 8.52 (d, *J* = 8.0 Hz, 1H), 7.97 (d, *J* = 9.0 Hz, 2H), 7.87 (td, *J* = 7.5, 2.0 Hz, 1H), 7.72 (dd, *J* = 5.5, 2.0 Hz, 1H), 7.36 (dd, *J* = 6.5, 5.0 Hz, 1H), 6.78 (d, *J* = 9.0 Hz, 2H), 3.14 (s, 6H).^13^C NMR (126 MHz, CDCl_3_) δ 159.8, 156.9, 155.1, 153.6, 149.4, 143.8, 137.1, 126.3, 124.1, 121.6, 116.1, 114.3, 111.5, 40.3. FT-IR (KBr pellet, cm^−1^): *ν*(N = N) 1452.9, m; *ν*(-CH_3_) 2919.7, s. ESI-MS (*m/z*): [M+H]^+^ calculated for C_18_H_17_N_5_, 304.1; found, 304.1.

### Synthesis of [Ir(dfppy)_2_(p-azobpy)]Cl (Ir1)

A mixture of [Ir(dfppy)_2_Cl]_2_ (61 mg, 0.05 mmol) and the p-azobpy ligand (39 mg, 0.15 mmol ) was suspended in 20 mL MeOH/CHCl_3_ (1:1, v/v) and heated at 65 °C for 6 h under argon. The solution was filtered, and the precipitate was washed thrice with 2 mL cold methanol. The filtrate was evaporated under reduced pressure to remove the solvent. The crude product was purified by column chromatography on alumina using dichloromethane and acetone (5:1, v/v) as the eluent, obtaining an orange-yellow powder (56.7 mg, 68.1%). ^1^H NMR (500 MHz, DMSO-d_6_) δ 9.29 (s, 1H), 9.18 (d, J = 8.5 Hz, 1H), 8.37 (t, J = 8.5 Hz, 1H), 8.33 (dd, J = 8.5, 4.5 Hz, 2H), 8.13 (d, J = 6.0 Hz, 1H), 8.08 (t, J = 8.0 Hz, 2H), 8.03 (d, J = 8.5 Hz, 2H), 7.99 (d, J = 6.0 Hz, 2H), 7.89 (d, J = 5.0 Hz, 1H), 7.85–7.67 (m, 5H), 7.35–7.23 (m, 2H), 7.01 (t, J = 11.0 Hz, 2H), 5.66 (ddd, J = 8.0, 5.5, 2.5 Hz, 2H). ^13^C NMR (126 MHz, DMSO-d_6_) δ 171.9, 164.4, 164.3, 163.2, 163.1, 162.3, 162.3, 162.2, 160.2, 160.1, 158.8, 157.9, 155.3, 154.9, 154.8, 154.7, 152.5, 152.3, 150.8, 150.5, 150.3, 140.8, 140.6, 134.4, 130.4, 130.2, 129.9, 128.1, 126.4, 125.1, 123.9, 120.6, 119.4, 113.8, 113.7, 99.8, 99.6, 99.4. FT-IR (KBr pellet, cm^−1^): *ν*(N = N) 1429.7, m. ESI-MS (*m/z*) [M-Cl]^+^ calculated for C_38_H_24_F_4_IrN_6_, 833.1; found, 833.4.

### Synthesis of [Ir(ppy)_2_(p-azobpy)]Cl (Ir2)

This complex was synthesized by a method identical to that described for the preparation of **Ir1,** except that [Ir(ppy)_2_Cl]_2_ (54 mg, 0.05 mmol) was used instead of [Ir(dfppy)_2_Cl]_2_. **Ir2** was isolated as a yellow powder (45.1 mg, 59.3%). ^1^H NMR (500 MHz, DMSO-d_6_) δ 9.23 (s, 1H), 9.12 (d, J = 8.0 Hz, 1H), 8.30 (dd, J = 15.0, 7.5 Hz, 3H), 8.06 (d, J = 6.0 Hz, 1H), 7.99 (t, J = 8.0 Hz, 3H), 7.97–7.92 (m, 4H), 7.91 (d, J = 5.0 Hz, 1H), 7.79 (d, J = 5.5 Hz, 1H), 7.75 (d, J = 7.0 Hz, 1H), 7.71 (t, J = 7.5 Hz, 3H), 7.66 (d, J = 5.5 Hz, 1H), 7.17 (dt, J = 12.5, 6.0 Hz, 2H), 7.03 (t, J = 7.0 Hz, 2H), 6.92 (t, J = 7.0 Hz, 2H), 6.21 (t, J = 7.0 Hz, 2H). ^13^C NMR (126 MHz, DMSO-d_6_) δ 167.3, 167.2, 158.6, 158.1, 155.6, 152.3, 152.1, 150.8, 150.7, 150.4, 149.8, 149.5, 144.3, 140.3, 139.3, 134.3, 131.5, 130.8, 130.4, 129.5, 126.2, 125.6, 124.5, 123.9, 122.9, 120.5, 119.0. FT-IR (KBr pellet, cm^−1^): *ν*(N = N) 1437.3, m. ESI-MS (*m/z*) [M-Cl]^+^ calculated for C_38_H_28_IrN_6_, 761.2; found, 761.3.

### Synthesis of [Ir(2-pq)_2_(p-azobpy)]Cl (Ir3)

This complex was synthesised by a method identical to that described for the preparation of **Ir1,** except that [Ir(2-pq)_2_Cl]_2_ (64 mg, 0.05 mmol) was used instead of [Ir(dfppy)_2_Cl]_2_. **Ir3** was isolated as an orange-yellow powder (52.3 mg, 60.7%). ^1^H NMR (500 MHz, DMSO-d_6_) δ 8.84 (d, *J* = 1.5 Hz, 1H), 8.70 (d, *J* = 8.0 Hz, 1H), 8.65–8.54 (m, 4H), 8.32 (dd, *J* = 13.5, 6.5 Hz, 3H), 8.20–8.08 (m, 2H), 8.04–7.89 (m, 5H), 7.69 (ddd, *J* = 27.0, 13.5, 6.5 Hz, 4H), 7.43 (t, *J* = 7.0 Hz, 2H), 7.31 (d, *J* = 9.0 Hz, 1H), 7.24 (d, *J* = 9.0 Hz, 1H), 7.18 (dd, *J* = 13.5, 7.0 Hz, 3H), 7.13–7.06 (m, 1H), 6.84 (dd, *J* = 14.0, 7.0 Hz, 2H), 6.43 (dd, *J* = 15.5, 7.5 Hz, 2H). ^13^C NMR (126 MHz, DMSO-d_6_) δ 170.2, 170.1, 158.4, 157.6, 155.0, 152.2, 151.1, 151.1, 149.6, 147.9, 147.2, 146.2, 141.0, 140.4, 134.4, 134.2, 134.1, 131.7, 131.5, 131.1, 130.3, 129.9, 129.4, 129.3, 128.7, 128.3, 128.2, 127.9, 127.9, 127.3, 125.3, 124.5, 124.4, 123.9, 123.3, 120.2, 118.8, 118.7, 118.1. FT-IR (KBr pellet, cm^−1^): *ν*(N = N) 1443.2, m. ESI-MS (*m/z*) [M-Cl]^+^ calculated for C_46_H_32_IrN_6_, 861.2; found, 861.3.

### Synthesis of [Ir(dbq)_2_(p-azobpy)]Cl (Ir4)

This complex was synthesised by a method identical to that described for the preparation of **Ir1,** except that [Ir(dbq)_2_Cl]_2_ (69 mg, 0.05 mmol) was used instead of [Ir(dfppy)_2_Cl]_2_. **Ir4** was isolated as an orange-yellow powder (48.8 mg, 53.6%). ^1^H NMR (500 MHz, DMSO-d_6_) δ 9.29 (d, *J* = 2.0 Hz, 1H), 9.18 (t, *J* = 8.5 Hz, 3H), 8.88 (dd, *J* = 8.5, 3.0 Hz, 2H), 8.83 (d, *J* = 8.0 Hz, 2H), 8.40 (d, *J* = 3.0 Hz, 1H), 8.38–8.31 (m, 3H), 8.21 (d, *J* = 3.0 Hz, 1H), 8.09 (d, *J* = 6.0 Hz, 1H), 7.97 (dd, *J* = 19.5, 7.0 Hz, 5H), 7.92–7.85 (m, 3H), 7.69 (dd, *J* = 16.5, 9.0 Hz, 4H), 7.32 (td, *J* = 8.0, 2.5 Hz, 2H), 6.47 (dd, *J* = 7.5, 2.0 Hz, 2H). ^13^C NMR (126 MHz, DMSO-d_6_) δ 158.9, 158.5, 155.9, 153.3, 152.3, 151.6, 148.9, 148.7, 146.2, 146.2, 143.7, 143.5, 142.9, 140.7, 138.0, 137.9, 134.4, 132.8, 132.5, 131.9, 131.4, 130.4, 129.8, 129.0, 128.8, 125.3, 124.5, 123.9, 119.1, 117.1, 69.9. FT-IR (KBr pellet, cm^−1^): *ν*(N = N) 1443.7, m. ESI-MS (*m/z*) [M-Cl]^+^ calculated for C_48_H_30_IrN_8_, 911.2; found, 911.3.

### Synthesis of [Ir(dfppy)_2_(dmap-azobpy)]Cl (Ir5)

This complex was synthesized by a method identical to that described for the preparation of **Ir1,** except that the ligand dmap-azobpy (46 mg, 0.15 mmol) was used instead of ligand p-azobpy. **Ir5** was isolated as a red powder (58.2 mg, 66.4%). ^1^H NMR (500 MHz, DMSO-d_6_) δ 9.09 (d, J = 8.5 Hz, 1H), 9.05 (s, 1H), 8.32 (dd, J = 12.5, 6.5 Hz, 3H), 8.06 (dd, J = 12.0, 8.0 Hz, 2H), 7.94 (t, J = 5.5 Hz, 2H), 7.90 (d, J = 9.5 Hz, 2H), 7.84 (d, J = 4.0 Hz, 2H), 7.74 (t, J = 6.0 Hz, 2H), 7.30–7.23 (m, 2H), 6.99 (dd, J = 12.5, 9.5 Hz, 2H), 6.93 (d, J = 9.5 Hz, 2H), 5.67–5.61 (m, 2H), 3.17 (s, 6H). ^13^C NMR (126 MHz, DMSO-d_6_) δ 164.4, 163.2, 163.2, 162.3, 162.2, 160.2, 160.1, 159.8, 157.2, 155.7, 155.4, 155.3, 155.1, 154.9, 151.8, 150.7, 150.3, 150.1, 143.5, 140.7, 140.5, 129.7, 128.1, 127.4, 126.1, 125.1, 123.9, 123.8, 119.8, 118.7, 113.8, 113.6, 112.5, 99.7, 99.5, 99.3. FT-IR (KBr pellet, cm^−1^): *ν*(N = N) 1430.1, m; *ν*(-CH_3_) 2915.9, s. ESI-MS (*m/z*) [M-Cl]^+^ calculated for C_40_H_29_F_4_IrN_7_, 876.2; found, 876.3.

### Synthesis of [Ir(ppy)_2_(dmap-azobpy)]Cl (Ir6)

This complex was synthesized by a method identical to that described for the preparation of **Ir1,** except that [Ir(ppy)_2_Cl]_2_ (54 mg, 0.05 mmol) was used instead of [Ir(dfppy)_2_Cl]_2_, and the ligand dmap-azobpy (46 mg, 0.15 mmol) was used instead of ligand p-azobpy. **Ir6** was isolated as a red powder (43.8 mg, 54.5%). ^1^H NMR (500 MHz, CDCl_3_) δ 9.07 (s, 1H), 9.00 (s, 1H), 8.41 (s, 1H), 8.19 (s, 2H), 7.95 (d, *J* = 7.5 Hz, 3H), 7.91 (d, *J* = 5.0 Hz, 1H), 7.80 (s, 3H), 7.71 (d, *J* = 7.0 Hz, 2H), 7.58 (s, 1H), 7.53 (d, *J* = 10.5 Hz, 2H), 7.04 (d, *J* = 6.5 Hz, 4H), 6.92 (d, *J* = 7.0 Hz, 4H), 6.32 (t, *J* = 6.5 Hz, 2H), 3.31 (s, 6H). ^13^C NMR (126 MHz, CDCl_3_) δ 168.0, 167.9, 156.9, 155.7, 151.2, 150.5, 150.3, 150.1, 148.5, 143.4, 140.6, 138.2, 131.8, 131.7, 130.9, 128.5, 125.8, 124.9, 123.4, 123.3, 122.7, 119.8, 118.7, 116.3, 115.4, 113.8, 41.3. FT-IR (KBr pellet, cm^−1^): *ν*(N = N) 1444.5, m; *ν*(-CH_3_) 2921.8, s. ESI-MS (*m/z*) [M-Cl]^+^ calculated for C_40_H_33_IrN_7_, 804.2; found, 804.3.

### Synthesis of [Ir(2-pq)_2_(dmap-azobpy)]Cl (Ir7)

This complex was synthesized by a method identical to that described for the preparation of **Ir1,** except that [Ir(2-pq)_2_Cl]_2_ (64 mg, 0.05 mmol) was used instead of [Ir(dfppy)_2_Cl]_2_, and the ligand dmap-azobpy (46 mg, 0.15 mmol) was used instead of ligand p-azobpy. **Ir7** was isolated as a dark red powder (56.8 mg, 62.8%). ^1^H NMR (500 MHz, DMSO-d_6_) δ 8.59 (tt, *J* = 14.5, 8.5 Hz, 6H), 8.31 (d, *J* = 8.0 Hz, 2H), 8.11 (dd, *J* = 15.5, 7.0 Hz, 3H), 7.94 (t, *J* = 8.5 Hz, 2H), 7.81 (d, *J* = 9.0 Hz, 3H), 7.73–7.65 (m, 1H), 7.42 (t, *J* = 6.5 Hz, 2H), 7.38 (d, *J* = 9.0 Hz, 1H), 7.24 (d, *J* = 9.0 Hz, 1H), 7.17 (dd, *J* = 13.0, 6.5 Hz, 3H), 7.09 (t, *J* = 8.0 Hz, 1H), 6.89 (d, *J* = 9.5 Hz, 2H), 6.83 (t, *J* = 7.5 Hz, 2H), 6.43 (t, *J* = 6.5 Hz, 2H), 3.14 (s, 6H). ^13^C NMR (126 MHz, DMSO-d_6_) δ 170.2, 159.3, 157.0, 155.6, 154.9, 151.7, 151.4, 148.8, 147.7, 147.3, 147.2, 146.3, 143.3, 140.9, 140.3, 134.2, 134.2, 131.6, 131.4, 131.1, 129.9, 128.9, 128.2, 127.9, 127.3, 127.2, 125.1, 124.7, 124.5, 123.2, 119.3, 118.7, 117.4, 112.5. FT-IR (KBr pellet, cm^−1^): *ν*(N = N) 1444.0, m; *ν*(-CH_3_) 2917.9, s. ESI-MS (*m/z*) [M-Cl]^+^ calculated for C_48_H_37_IrN_7_, 904.3; found, 904.3.

### Synthesis of [Ir(dbq)_2_(dmap-azobpy)]Cl (Ir8)

This complex was synthesized by a method identical to that described for the preparation of **Ir1,** except that [Ir(dbq)_2_Cl]_2_ (69 mg, 0.05 mmol) was used instead of [Ir(dfppy)_2_Cl]_2_, and the ligand dmap-azobpy (46 mg, 0.15 mmol) was used instead of ligand p-azobpy. Complex **Ir8** was isolated as a dark red powder (53.6 mg, 56.2%). ^1^H NMR (500 MHz, DMSO-d_6_) δ 9.19–9.07 (m, 4H), 8.87 (dd, J = 7.0, 3.0 Hz, 2H), 8.82 (d, J = 8.0 Hz, 2H), 8.33 (d, J = 7.5 Hz, 3H), 8.21 (d, J = 3.0 Hz, 1H), 7.95–7.92 (m, 3H), 7.87 (t, J = 8.5 Hz, 4H), 7.75 (d, J = 6.0 Hz, 1H), 7.69–7.61 (m, 1H), 7.31 (t, J = 7.5 Hz, 3H), 6.90 (d, J = 9.5 Hz, 2H), 6.67 (s, 1H), 6.46 (d, J = 7.5 Hz, 2H), 3.14 (s, 6H). ^13^C NMR (126 MHz, DMSO-d_6_) δ 171.6, 159.7, 157.8, 156.3, 154.9, 152.6, 151.6, 149.0, 146.2, 143.4, 142.9, 140.6, 137.9, 132.8, 132.5, 131.9, 131.4, 129.8, 129.5, 128.9, 128.8, 125.3, 124.5, 119.9, 118.5, 117.0, 112.5, 23.0. FT-IR (KBr pellet, cm^−1^): *ν*(N = N) 1443.0, m; *ν*(-CH_3_) 2923.7, s. ESI-MS (*m/z*) [M-Cl]^+^ calculated for C_50_H_35_IrN_9_, 954.3; found, 954.3.

### Synthesis of [Ir(dfppy)_2_(bpy-NH_2_)]Cl (IrNH_2_1)

This complex was synthesized by a method identical to that described for the preparation of **Ir1,** except that the ligand bpy-NH_2_ (26 mg, 0.15 mmol) was used instead of ligand p-azobpy. **IrNH**_**2**_**1** was isolated as a green-yellow powder (43.6 mg, 58.6%). ^1^H NMR (500 MHz, DMSO-d_6_) δ 8.43 (d, *J* = 8.0 Hz, 1H), 8.32–8.23 (m, 3H), 8.04 (dd, *J* = 14.5, 7.5 Hz, 2H), 7.87 (d, *J* = 4.5 Hz, 1H), 7.84–7.73 (m, 2H), 7.68 (d, *J* = 5.0 Hz, 1H), 7.66–7.62 (m, 1H), 7.32 (t, *J* = 7.5 Hz, 1H), 7.29–7.17 (m, 4H), 6.92 (t, *J* = 14.0 Hz, 2H), 6.70 (dd, *J* = 6.5, 2.5 Hz, 1H), 5.64 (dd, *J* = 8.5, 2.5 Hz, 1H), 5.58 (dd, *J* = 8.5, 2.5 Hz, 1H). ^13^C NMR (126 MHz, DMSO-d_6_) δ 163.6, 163.2, 162.1, 157.0, 156.7, 156.4, 155.9, 154.8, 150.5, 149.8, 149.7, 149.4, 140.4, 140.3, 140.2, 128.9, 128.3, 127.9, 124.9, 124.4, 123.8, 123.7, 123.7, 123.6, 113.9, 113.8, 113.6, 113.4, 112.5, 109.5, 99.2, 99.1. FT-IR (KBr pellet, cm^−1^): *ν*(-NH_2_) 3443.0, s. ESI-MS (*m/z*) [M-Cl]^+^ calculated for C_32_H_21_F_4_IrN_5_, 744.1; found, 744.3.

### Synthesis of [Ir(dbq)_2_(bpy-NH_2_)]Cl (IrNH_2_4)

This complex was synthesized by a method identical to that described for the preparation of **Ir1,** except that [Ir(dbq)_2_Cl]_2_ (69 mg, 0.05 mmol) was used instead of [Ir(dfppy)_2_Cl]_2_, and the ligand bpy-NH_2_ (26 mg, 0.15 mmol) was used instead of ligand p-azobpy. **IrNH**_**2**_**4** was isolated as a red powder (42.2 mg, 51.3%). ^1^H NMR (500 MHz, DMSO-d_6_) δ 9.24–9.16 (m, 2H), 8.91 (dd, *J* = 26.0, 2.5 Hz, 2H), 8.82 (d, *J* = 8.0 Hz, 2H), 8.50 (d, *J* = 8.0 Hz, 1H), 8.36–8.25 (m, 4H), 8.20 (d, *J* = 3.0 Hz, 1H), 7.95 (t, *J* = 7.5 Hz, 2H), 7.92–7.85 (m, 3H), 7.82 (s, 1H), 7.63–7.54 (m, 1H), 7.36–7.20 (m, 5H), 6.62 (d, *J* = 6.0 Hz, 1H), 6.47 (d, *J* = 7.5 Hz, 1H), 6.42 (d, *J* = 7.5 Hz, 1H). ^13^C NMR (126 MHz, DMSO-d_6_) δ 157.0, 156.9, 155.4, 152.1, 151.9, 151.3, 150.4, 150.1, 149.7, 146.2, 146.1, 143.1, 142.8, 142.7, 140.4, 138.2, 137.8, 132.9, 132.5, 132.4, 131.9, 131.8, 131.4, 129.9, 129.6, 129.0, 128.8, 128.7, 125.3, 124.5, 124.4, 116.7, 116.6, 112.4, 109.5, 79.7. FT-IR (KBr pellet, cm^−1^): *ν*(-NH_2_) 3464.2, s. ESI-MS (*m/z*) [M-Cl]^+^ calculated for C_42_H_27_IrN_7_, 822.2; found, 822.3.

### X-ray crystallography

Single crystals of **Ir1** that were suitable for an X-ray crystallographic study were grown from dichloromethane-toluene (1:1, v/v) at room temperature. An orange crystal was recorded on a Rigaku R-AXIS SPIDER IP diffractometer (MoKα, λ = 0.71073 Å) at 150(2) K. An absorption correction was applied using SADABS program^59^. The structure solution and full-matrix least-squares refinement based on F2 for **Ir1** were performed with the SHELXS-2014 and SHELXL-2014 program^60^, respectively, incorporated into the OLEX2 program package. Anisotropic thermal parameters were applied to all of the non-hydrogen atoms. All of the hydrogen atoms were included in calculated positions and were refined with isotropic thermal parameters riding on those of the parent atoms. Detailed crystallographic data for the crystal structural analysis were deposited with the Cambridge Crystallographic Data Centre, CCDC 1046980 (**Ir1**), containing the supplementary crystallographic data for the present study. The data can be obtained free of charge from The Cambridge Crystallographic Data Centre via www.ccdc.cam.ac.uk/data_request/cif.

### *In vitro* enzyme assay

Microsomes were prepared according to the method of Omura and Sato^61^. Rats (Wistar, 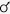 6 ~ 7 weeks) fasted overnight and were sacrificed by an intraperitoneal injection of 80 mg/kg pentobarbitalsodium. The liver containing 0.15 M KCl aq. (pH 7.4) was homogenized in three volumes of the same buffer. The hypoxic condition was generated by bubbling argon gas into the reaction solution for 30 minutes. Rat liver microsomes (0.25 mg protein/mL) and 50 μM NADPH were pre-incubated at 37 °C for 30 min. A phosphorescence probe (2.5 μM) containing 0.1% DMSO was added as a co-solvent. The initial reaction rate under hypoxia was determined by the initial slope of the time-dependent phosphorescence intensity change. The reaction mixture after the *in vitro* enzyme assay was extracted by chloroform and analyzed by ESI-MS.

### Cell culture

The HeLa cells were obtained from the ATCC and were cultured in DMEM supplemented with 10% inactivated fetal bovine serum and 1% penicillin-streptomycin (Gibco, USA). The cell lines were maintained at 37 °C in a humidified incubator containing 5% CO_2_. Cell lines were used within two months after resuscitation of frozen aliquots thawed from liquid nitrogen.

### D multicellular spheroid formation

3D multicellular spheroids were formed in agarose-coated 96-well plates as previously described^62^−^64^. Two hundred microliters of a 2.5 × 10^4^ cells/mL single cell suspension was plated onto agarose-coated (sterile, 0.75% (w/v) in PBS) 96-well imaging plates. The cells were allowed to aggregate for 96 h without motion, resulting in the formation of single spheroids with diameters of 400–500 μm per well. The small cell spheroids (diameters of 100–150 μm) were formed by the addition 200 μL of a 2.5 × 10^3^ cells/mL single cell suspension that was allowed to aggregate for 48 h.

### Cytotoxicity assay

Adherent 2D cell viability was measured using the MTT assay. Cells were plated at a density of 5 × 10^3^ cells/well in 96-well plates and incubated under standard culturing conditions for 24 h. Test compounds were then added at the indicated concentrations to quadruplicate wells. Control wells were prepared by the addition of culture medium. Wells containing culture medium without cells were used as blanks. The plates were incubated at 37** **°C in a 5% CO_2_ incubator for 12 h. Upon completion of the incubation, stock MTT dye solution (20 mL, 5 mg/mL) was added to each well. After additional 4 h incubation, 150 μL of DMSO was added to dissolve the MTT formazan. The optical density of each well was then measured on a microplate reader at a wavelength of 590 nm. The cytotoxicities of the probes to the 3D multicellular spheroids were evaluated using a cell spheroid growth inhibition assay^63^,^64^. The spheroids 400–500 μm in diameter were incubated with 2.5 μM Ir(III) complexes for 48 h. As an indication of spheroid proliferation, the diameters of the spheroids were measured with an inverted fluorescence microscope (Zeiss) after 6, 12, 24 and 48 h, and images were obtained.

### Cellular imaging

HeLa cells seeded on 35 mm glass-bottomed dishes were cultured overnight before the assay. Cells incubated under normoxic (20% O_2_) or hypoxic (1% O_2_ concentration cultured with or without DPI) conditions for 6 h were then treated with the Ir(III) complexes (final concentration 2.5 μM) for 30 min under the same conditions as the pre-culture. After washing the cells twice with phosphate buffer, phosphorescence images were captured using a Zeiss LSM 710 NLO confocal microscope (63 × /NA 1.4 oil immersion objective). The excitation wavelength of the laser was 405 nm, and the emission spectra were integrated over the range of 490–670 nm.3D multicellular spheroids with diameters of approximately 400–500 μm were harvested after approximately 4 days of growth. Test compounds were then added to the wells to achieve final concentrations of 2.5 μM and incubated for another 6 h. The cell spheroids were gently washed twice with phosphate buffer and imaged using a confocal microscope (10 × /NA 0.40 air objective lens).

## Additional Information

**How to cite this article**: Sun, L. *et al.* Azo-Based Iridium(III) Complexes as Multicolor Phosphorescent Probes to Detect Hypoxia in 3D Multicellular Tumor Spheroids. *Sci. Rep.* **5**, 14837; doi: 10.1038/srep14837 (2015).

## Supplementary Material

Supplementary Information

## Figures and Tables

**Figure 1:**
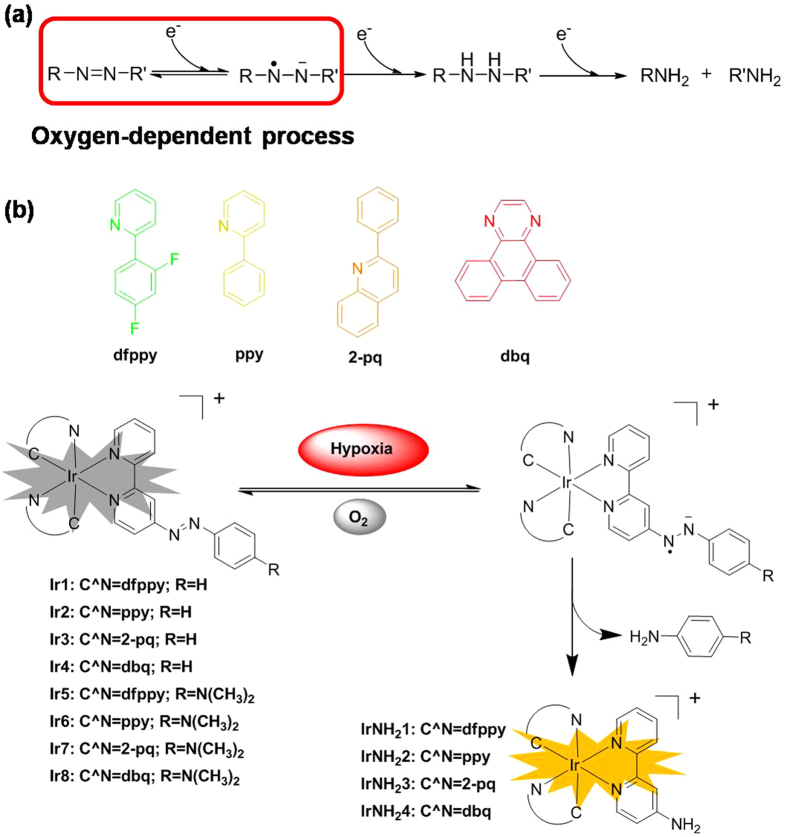
Detection mechanism and chemical structures of the phosphorescent Ir(III) complexes **Ir1**-**Ir8** synthesized and evaluated in this study.

**Table 1:** Oxidation and reduction potentials of various probes.

**Probe**	**azo^−1/−2^**	**azo^0/−1^**	**Ir^4+/3+^**
**Ir1**	−0.8442	−0. 6748	+1.0170
**Ir2**	−1.0966	−0. 7796	+1.0556
**Ir3**	−0.9228	−0. 6825	+1.1910
**Ir4**	−0.9362	−0. 7579	+1.0651
**Ir5**	−0.9006	−0. 7749	+1.2620
**Ir6**	−0.9332	−0. 8381	+1.2973
**Ir7**	−0.9016	−0. 7914	+1.3087
**Ir8**	−1.1233	−0. 8930	+1.0315
