# Supplementary material for: Azo-Based Iridium(III) Complexes as Multicolor Phosphorescent Probes to Detect Hypoxia in 3D Multicellular Tumor Spheroids
Source: Sci Rep. 2015 Oct 1;5:14837. doi: 10.1038/srep14837 (PMC4589790; doi:10.1038/srep14837)
Supplement: Supplementary Information [file srep14837-s1.doc]

Supporting Information

Azo-Based Iridium(III) Complexes as Multicolor Phosphorescent Probes to Detect Hypoxia in 3D Multicellular Tumor Spheroids

Lingli Sun, Guanying Li, Xiang Chen, Yu Chen,

Chengzhi Jin, Liangnian Ji, and Hui Chao*

*MOE Key Laboratory of Bioinorganic and Synthetic Chemistry, School of Chemistry and Chemical Engineering, Sun Yat-Sen University, Guangzhou 510275 China*

## *E-mail :* [*ceschh@mail.sysu.edu.cn*](mailto:ceschh@mail.sysu.edu.cn)

| **Table S1** Crystal data and structure refinement for **Ir1** | |
| --- | --- |
| Parameters | **Ir1** |
| Formula | C_38_H_24_ClF_4_IrN_6_ |
| Formula weight (g/mol） | 868.28 |
| Temp (K) | 150(2) |
| Cryst system M | Triclinic |
| Space group | P1 |
| *a*(Å) | 9.7144(10) |
| *b* (Å) | 11.4831(12) |
| *c*(Å) | 18.5077(19) |
| *α* (deg) | 75.720(2) |
| *β* (deg) | 76.254(2) |
| *γ* (deg) | 70.462(2) |
| V(Å^3^) | 1858.2(3) |
| Z | 2 |
| Calc. density (g/cm^3^) | 1.60142 |
| Absorption coefficient (mm^-1^) | 3.903 |
| F(000) | 3416 |
| Crystal size (mm) | 0.12 × 0.11 × 0.10 |
| Theta range for data collection | 6.082 to 54.916 |
| Index ranges | -12 ≤ h ≤ 11, -14 ≤ k ≤ 14, -23 ≤ l ≤ 23 |
| Reflections collected | 16679 |
| index ranges | 8380 [R_int_ = 0.0686, R_sigma_ = 0.1037] |
| Data/restraints/parameters | 8380/226/524 |
| Goodness-of-fit on F^2^ | 1.079 |
| Final R indices [I>2sigma(I)] | R_1_ = 0.0834, wR_2_ = 0.2099 |
| R indices (all data) | R_1_ = 0.1065, wR_2_ = 0.2263 |
| Largest diff. peak and hole | 3.85 and -2.59 e.^-3^ |

| **Table S2** Selected bond lengths (Å) and bond angles (°) for **Ir1** | | | |
| --- | --- | --- | --- |
| Bond lengths (Å) | | Bond lengths (Å) | |
| Ir(1)-N(1) | 2.061(9) | Ir(1)-C(12) | 1.999(10) |
| Ir(1)-N(2) | 2.049(9) | N(5)-N(6) | 1.26(2) |
| Ir(1)-N(3) | 2.125(10) | N(5)-C(30) | 1.432(18) |
| Ir(1)-N(4) | 2.111(9) | N(6)-C(33) | 1.418(16) |
| Ir(1)-C(7) | 1.974(10) |  |  |
| Bond angles (°) | | Bond angles (°) | |
| N(1)-Ir(1)-N(3) | 95.5(4) | C(7)-Ir(1)-N(4) | 97.0(4) |
| N(1)-Ir(1)-N(4) | 88.7(3) | C(7)-Ir(1)-C(12) | 88.6(4) |
| N(2)-Ir(1)-N(1) | 172.5(4) | C(12)-Ir(1)-N(1) | 94.5(4) |
| N(2)-Ir(1)-N(3) | 90.9(4) | C(12)-Ir(1)-N(2) | 80.6(4) |
| N(2)-Ir(1)-N(4) | 96.6(4) | C(12)-Ir(1)-N(3) | 97.5(4) |
| N(4)-Ir(1)-N(3) | 77.0(4) | C(12)-Ir(1)-N(4) | 173.9(4) |
| C(7)-Ir(1)-N(1) | 80.1(4) | N(6)-N(5)-C(30) | 115.5(16) |
| C(7)-Ir(1)-N(2) | 93.9(4) | N(5)-N(6)-C(33) | 117.4(15) |
| C(7)-Ir(1)-N(3) | 172.8(4) |  |  |

**Table S3** Photoluminescence Data for reduced amino products (**IrNH_2_1-IrNH_2_4**)

| Complexes | Τ(ns) | Φ_em_ |
| --- | --- | --- |
| **IrNH_2_1** | 926 | 0.63 |
| **IrNH_2_2** | 334 | 0.04 |
| **IrNH_2_3** | 937 | 0.14 |
| **IrNH_2_4** | 133 | 0.10 |


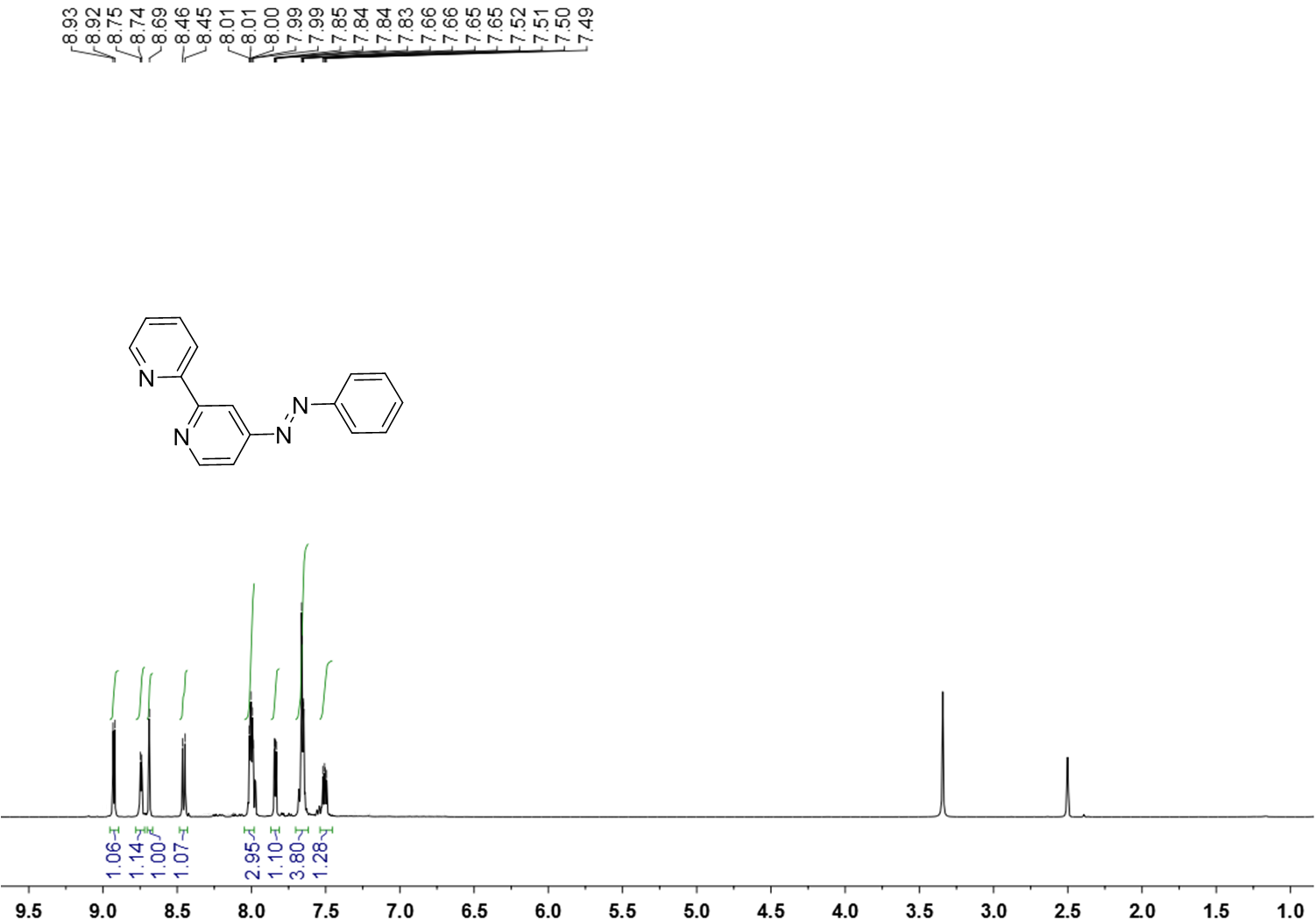


**Figure S1.** ^1^H NMR spectrum of **p-azobpy** in DMSO-d_6_, 500 MHz.


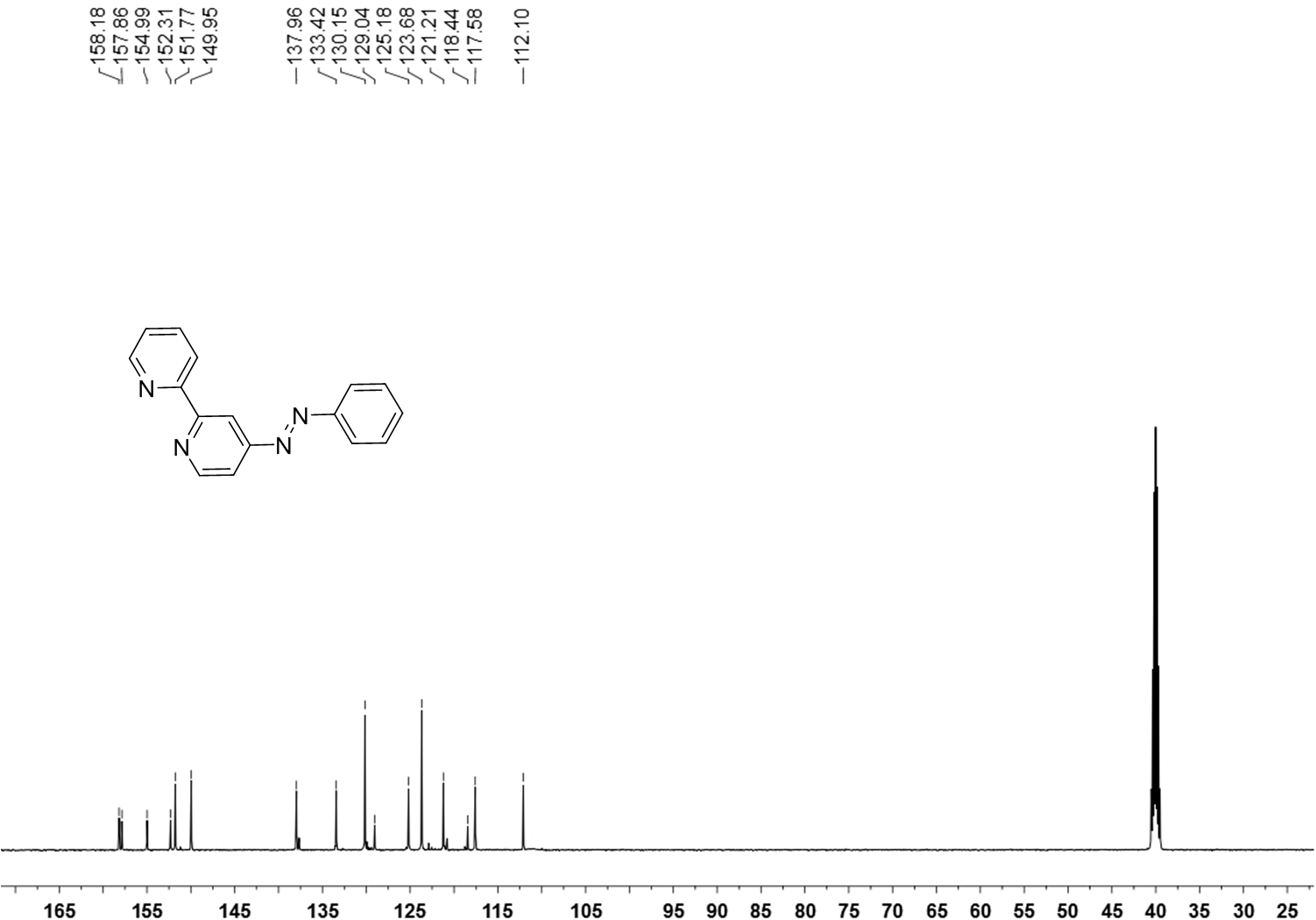


**Figure S2.** ^13^C NMR spectrum of **p-azobpy** in DMSO-d_6_, 126 MHz.


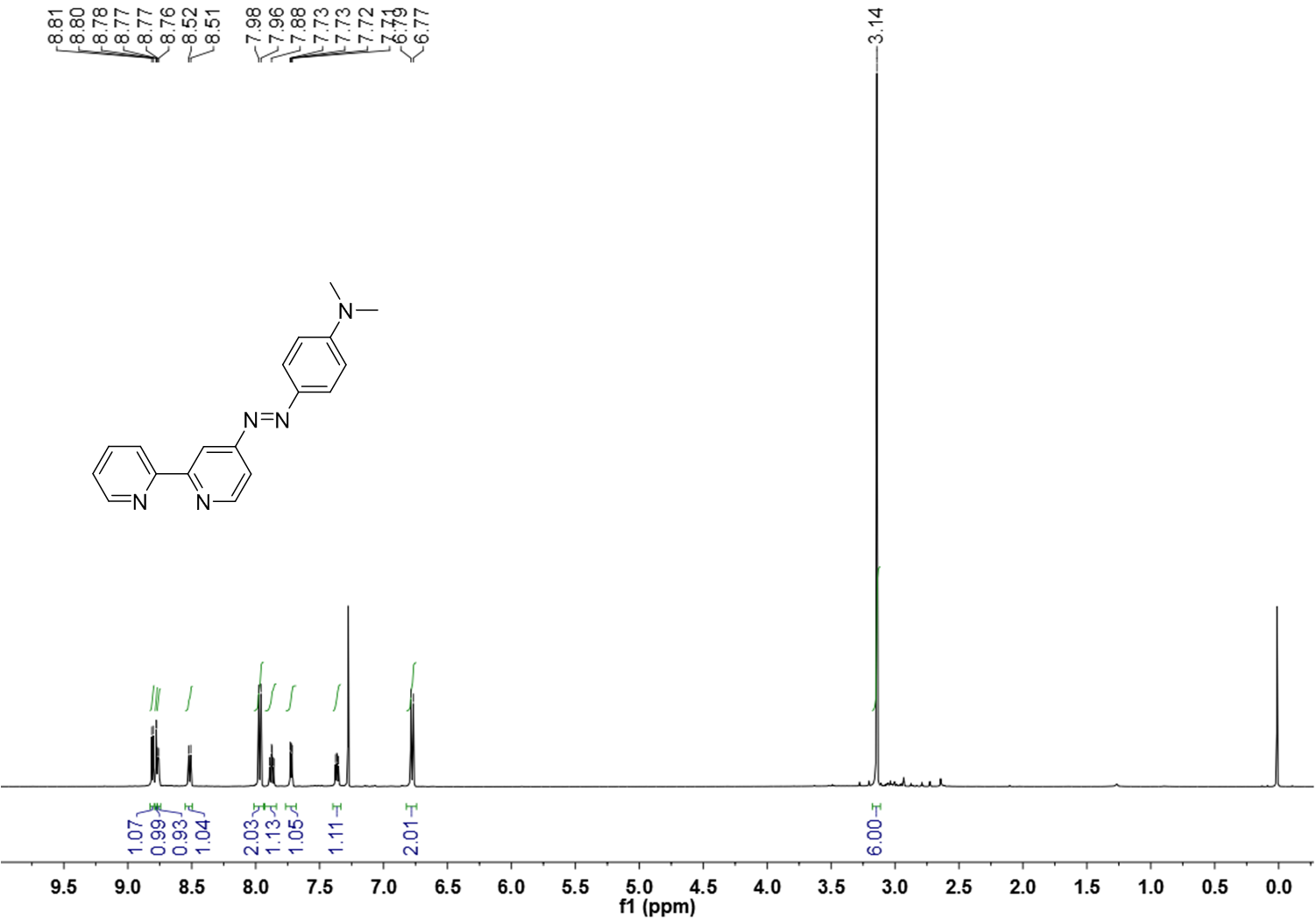


**Figure S3.** ^1^H NMR spectrum of **dmap-azobpy** in CDCl_3_, 500 MHz.


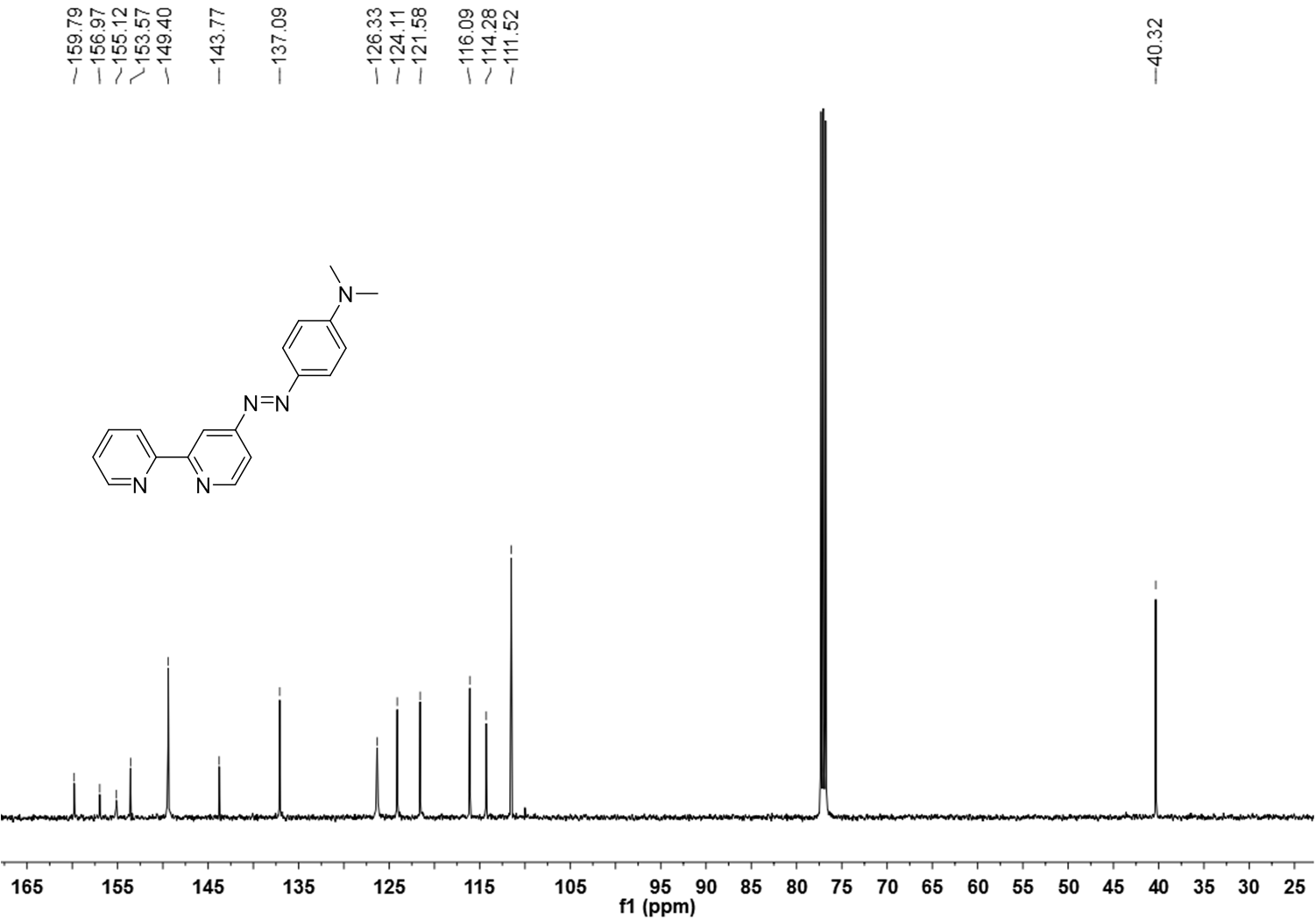


**Figure S4.** ^13^C NMR spectrum of **dmap-azobpy** in CDCl_3_, 126 MHz.

**
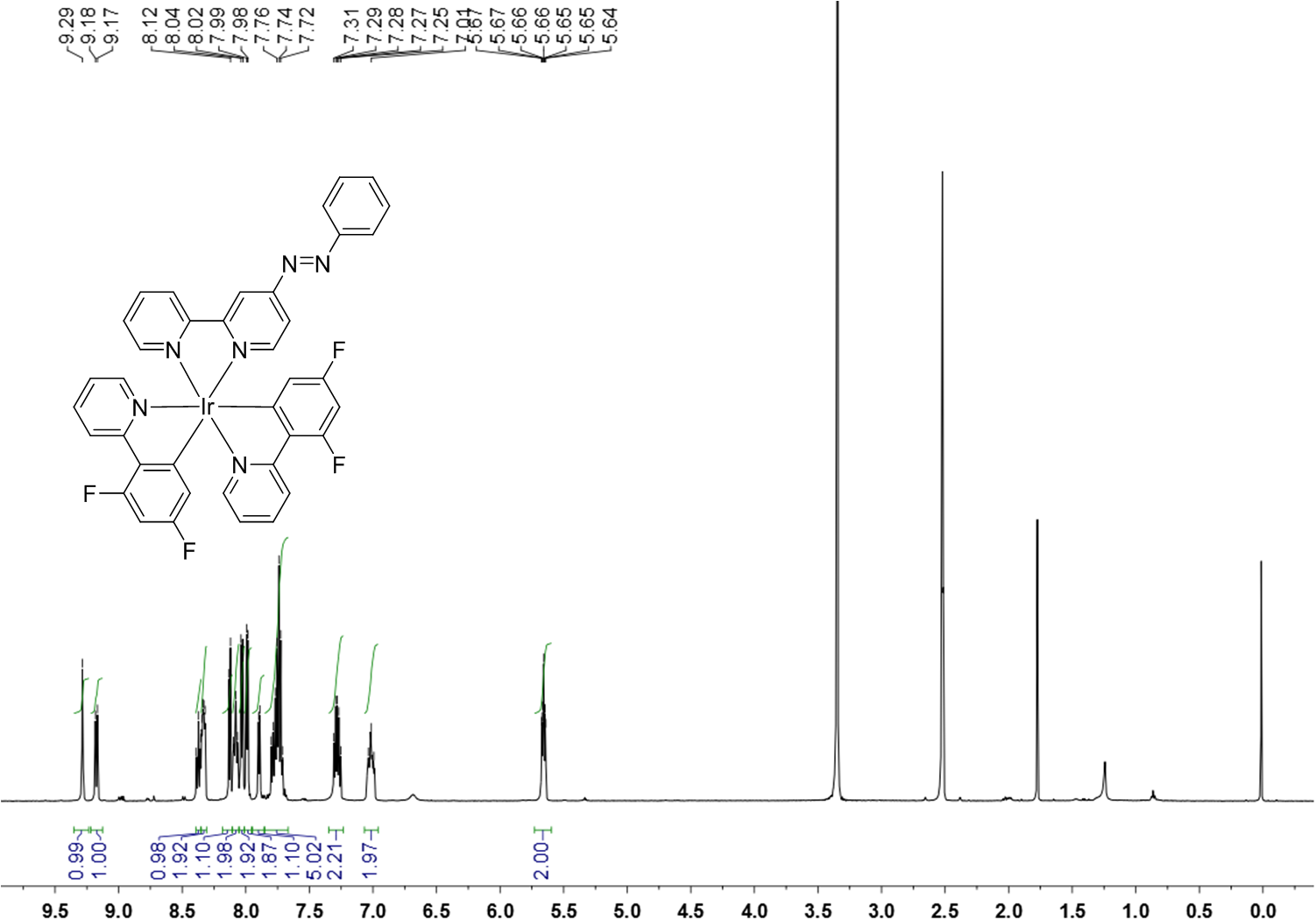
**

**Figure S5*.*** ^1^H NMR spectrum of **Ir1** in DMSO-d_6_, 500 MHz.


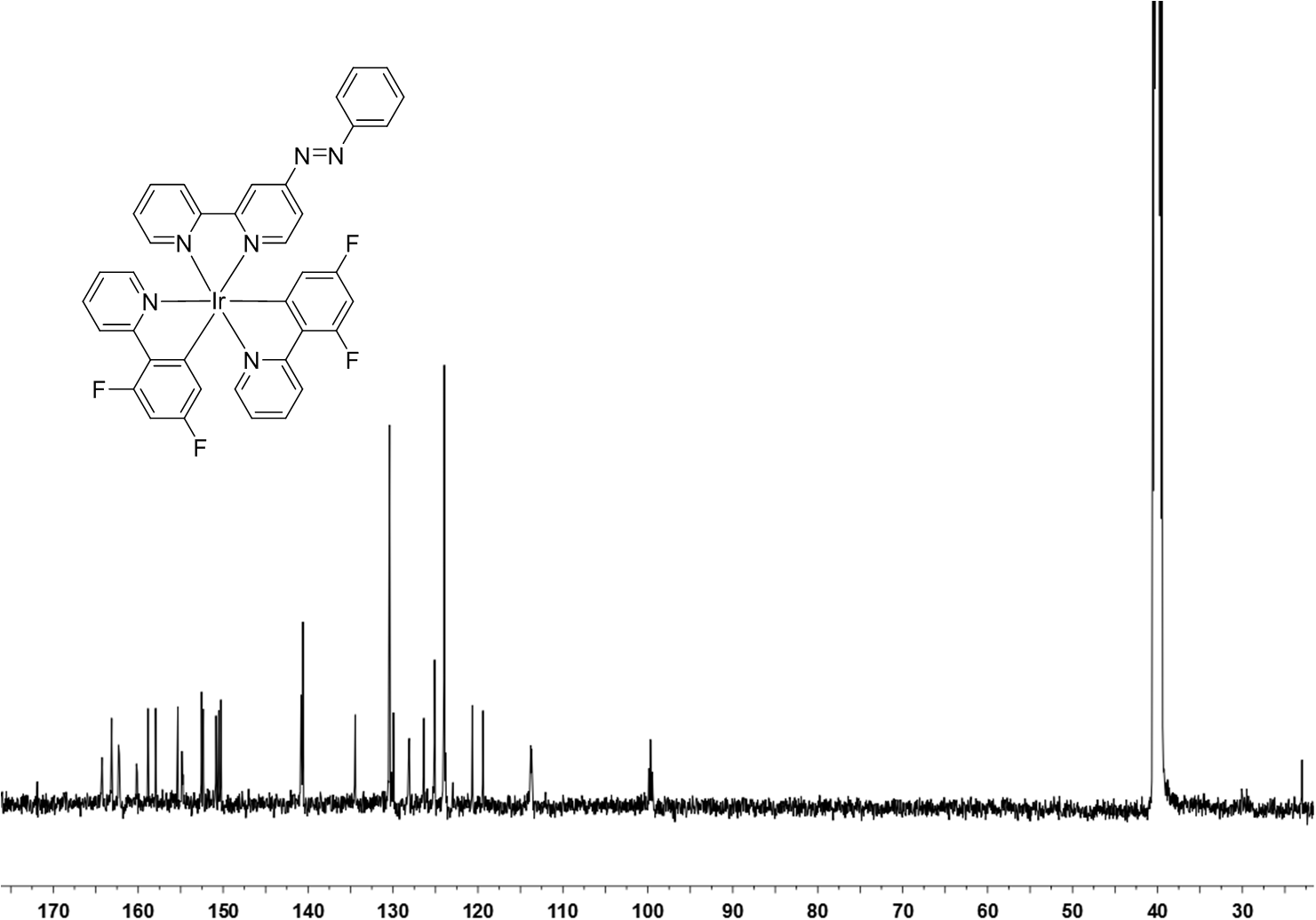


**Figure S6.** ^13^C NMR spectrum of **Ir1** in DMSO-d_6_, 126 MHz.


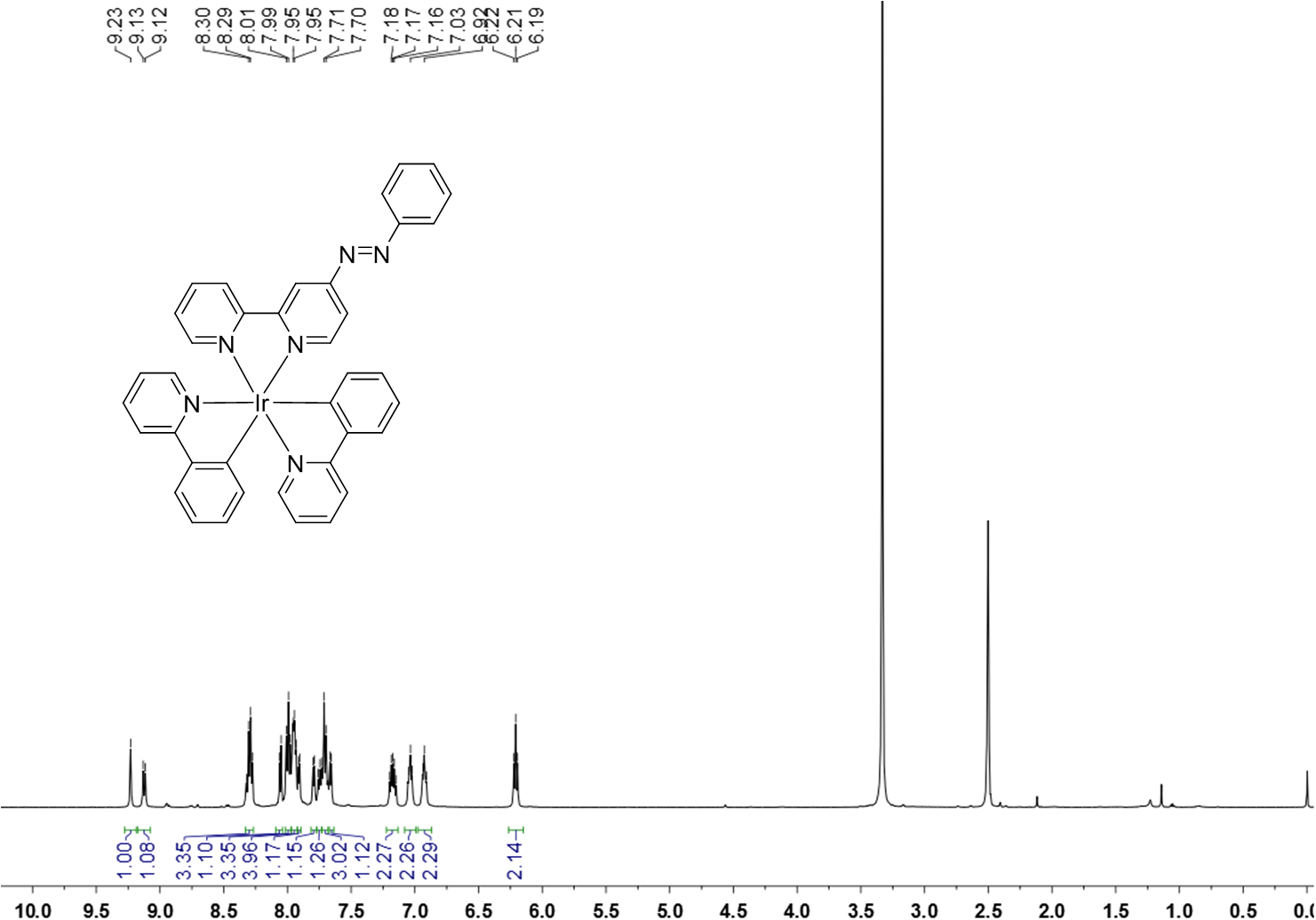


**Figure S7*.*** ^1^H NMR spectrum of **Ir2** in DMSO-d_6_, 500 MHz.


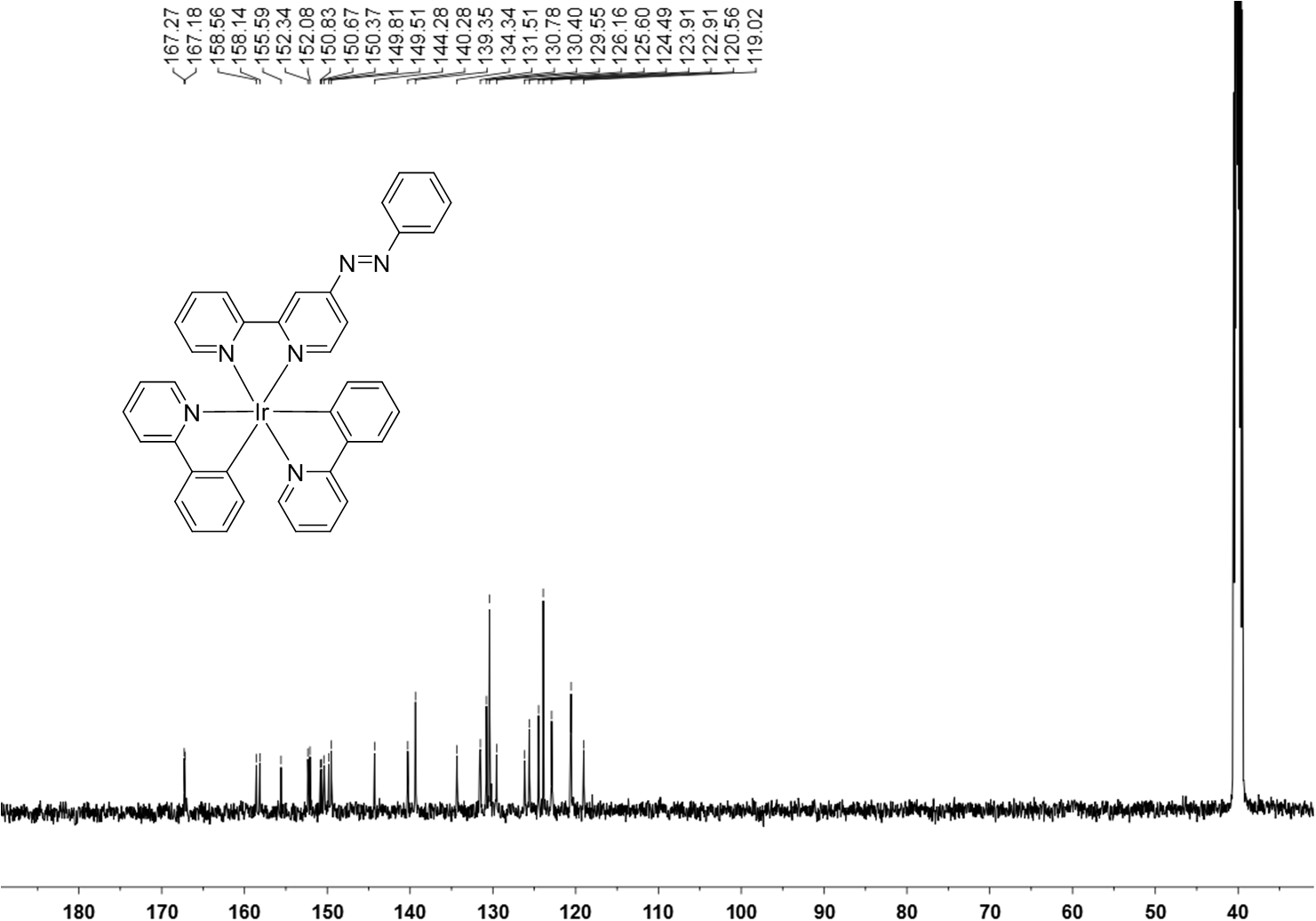


**Figure S8*.*** ^13^C NMR spectrum of **Ir2** in DMSO-d_6_, 126 MHz.


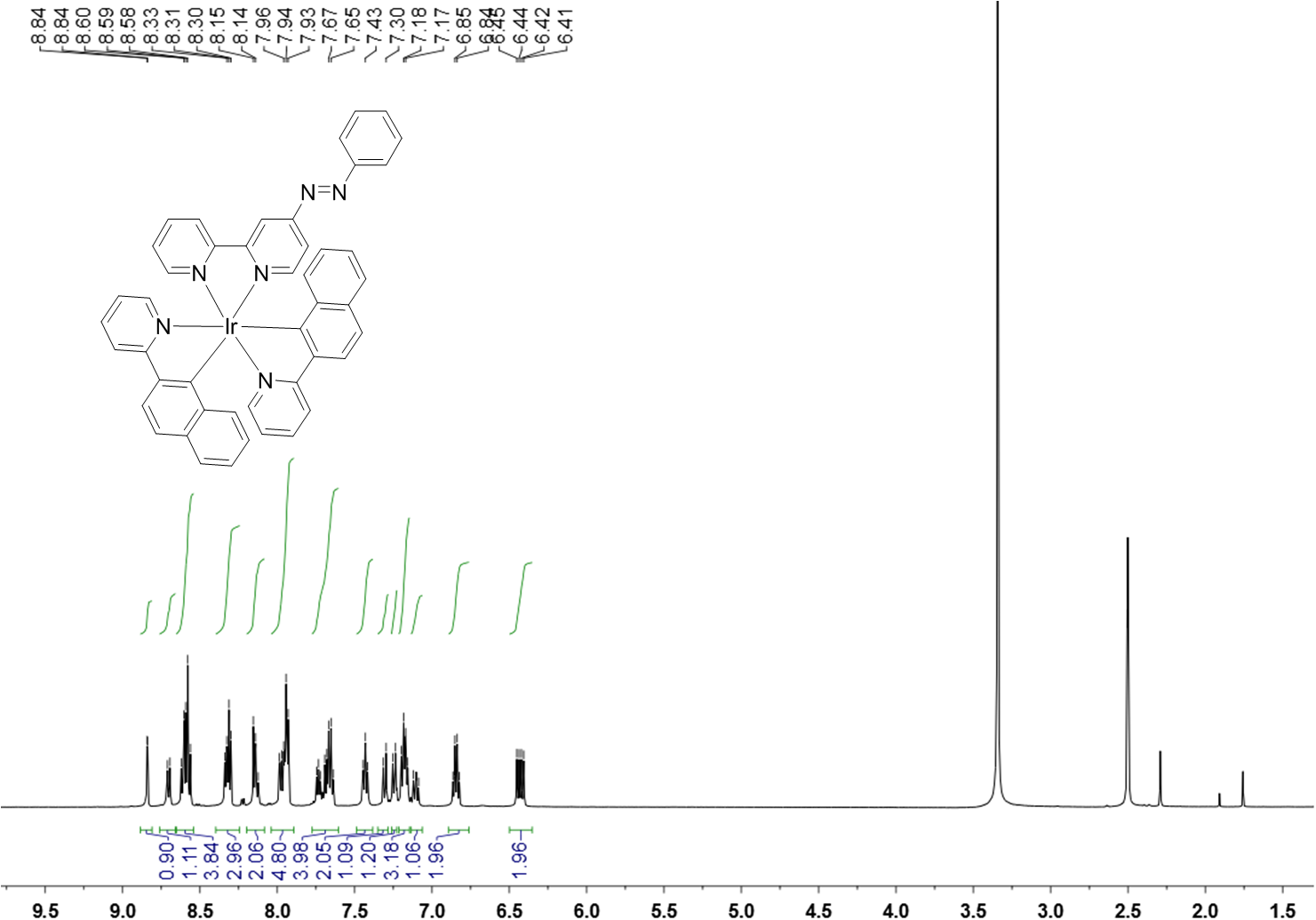


**Figure S9*.*** ^1^H NMR spectrum of **Ir3** in DMSO-d_6_, 500 MHz.


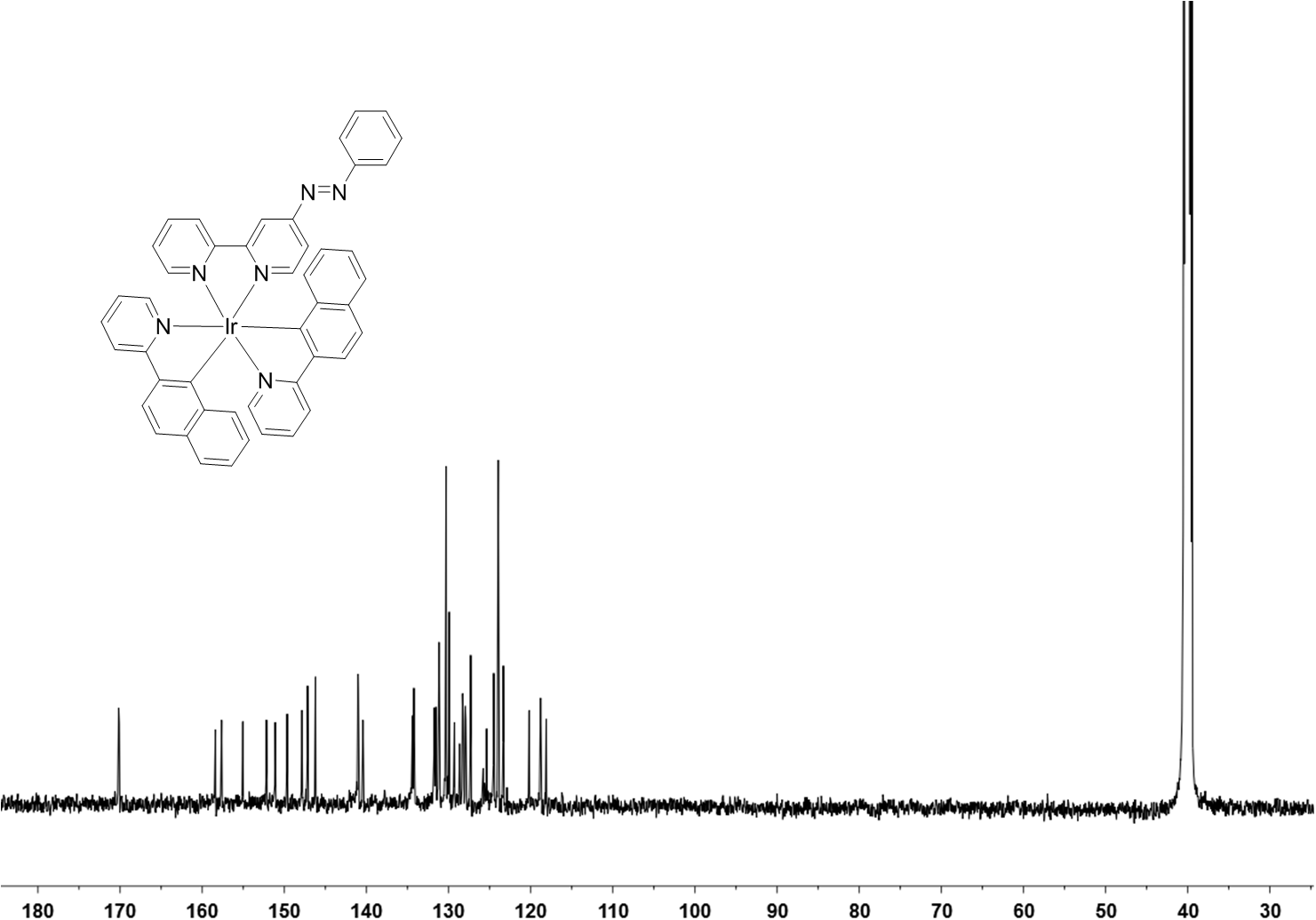


**Figure S10*.*** ^13^C NMR spectrum of **Ir3** in DMSO-d_6_, 126 MHz.


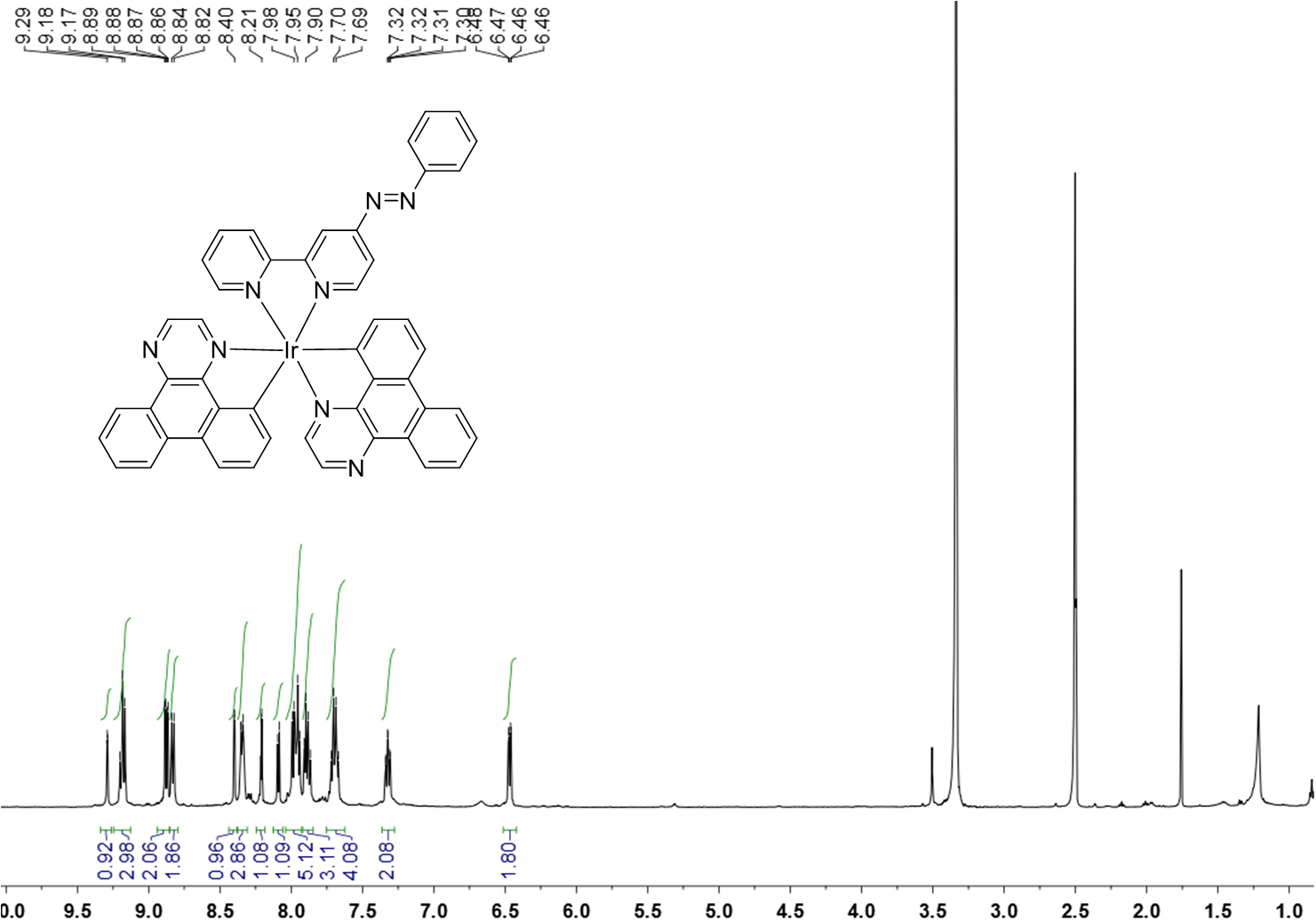


**Figure S11.** ^1^H NMR spectrum of **Ir4** in DMSO-d_6_, 500 MHz.


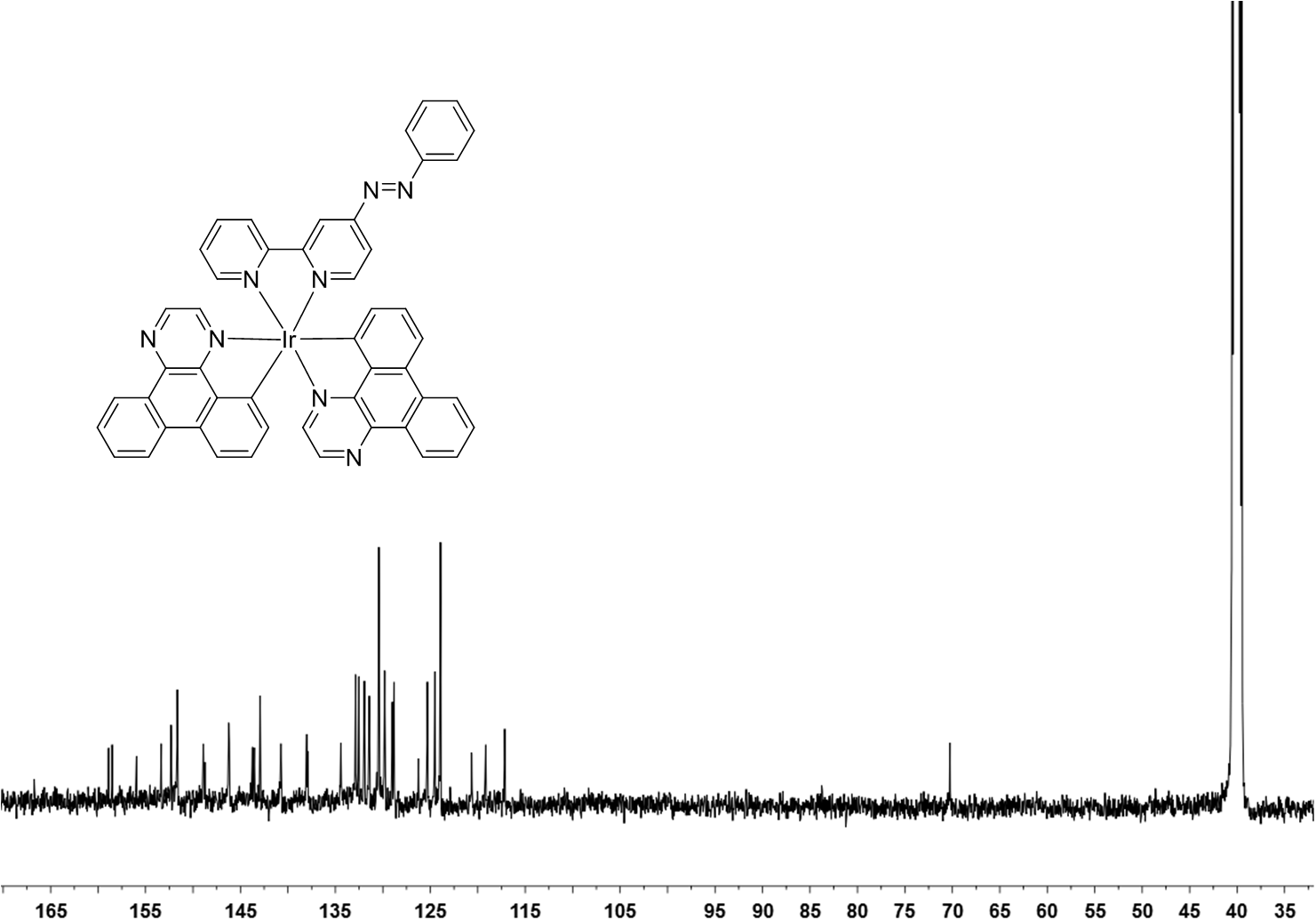


**Figure S12*.*** ^13^C NMR spectrum of **Ir4** in DMSO-d_6_, 126 MHz.


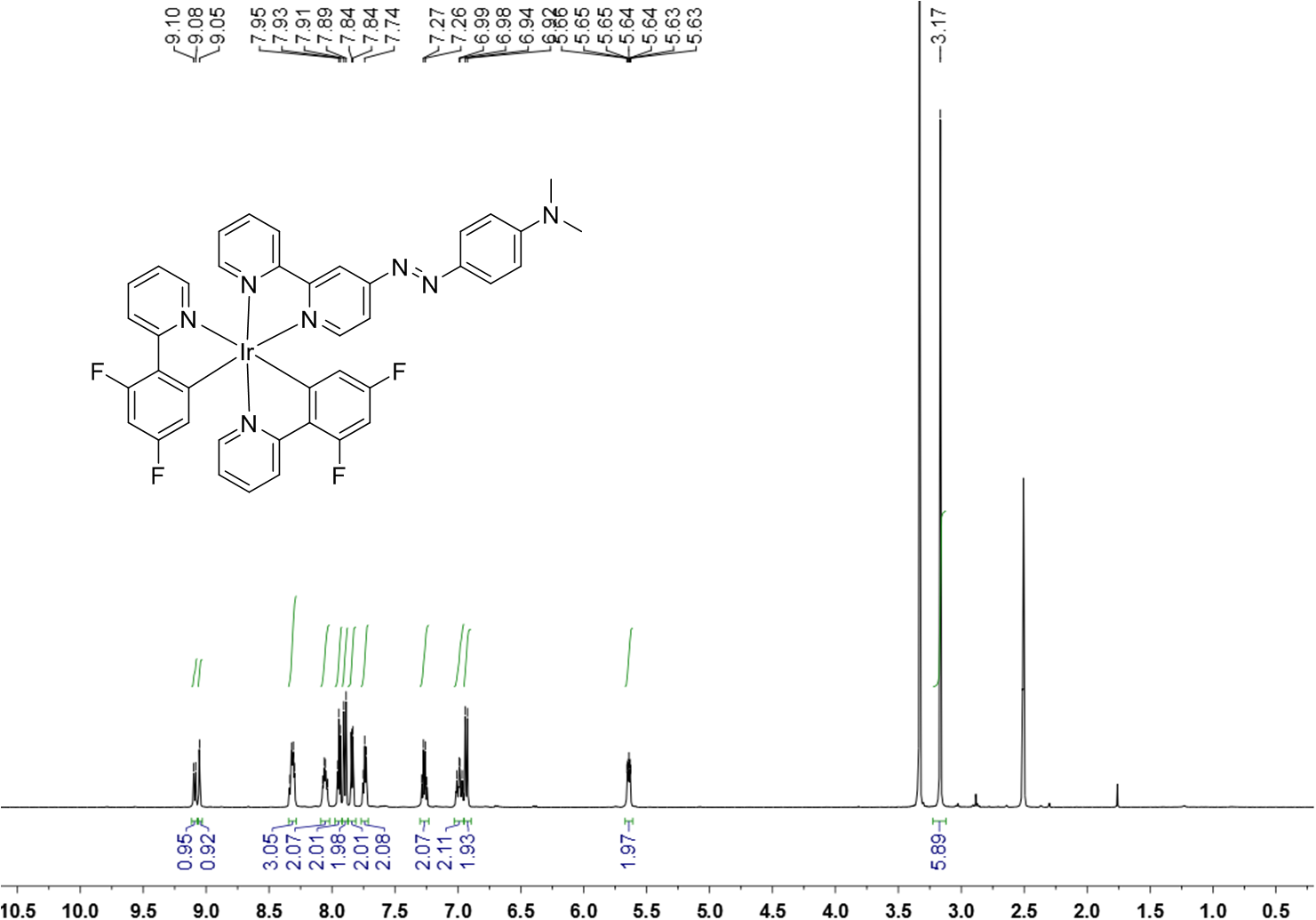


**Figure S13.** ^1^H NMR spectrum of **Ir5** in DMSO-d_6_, 500 MHz.


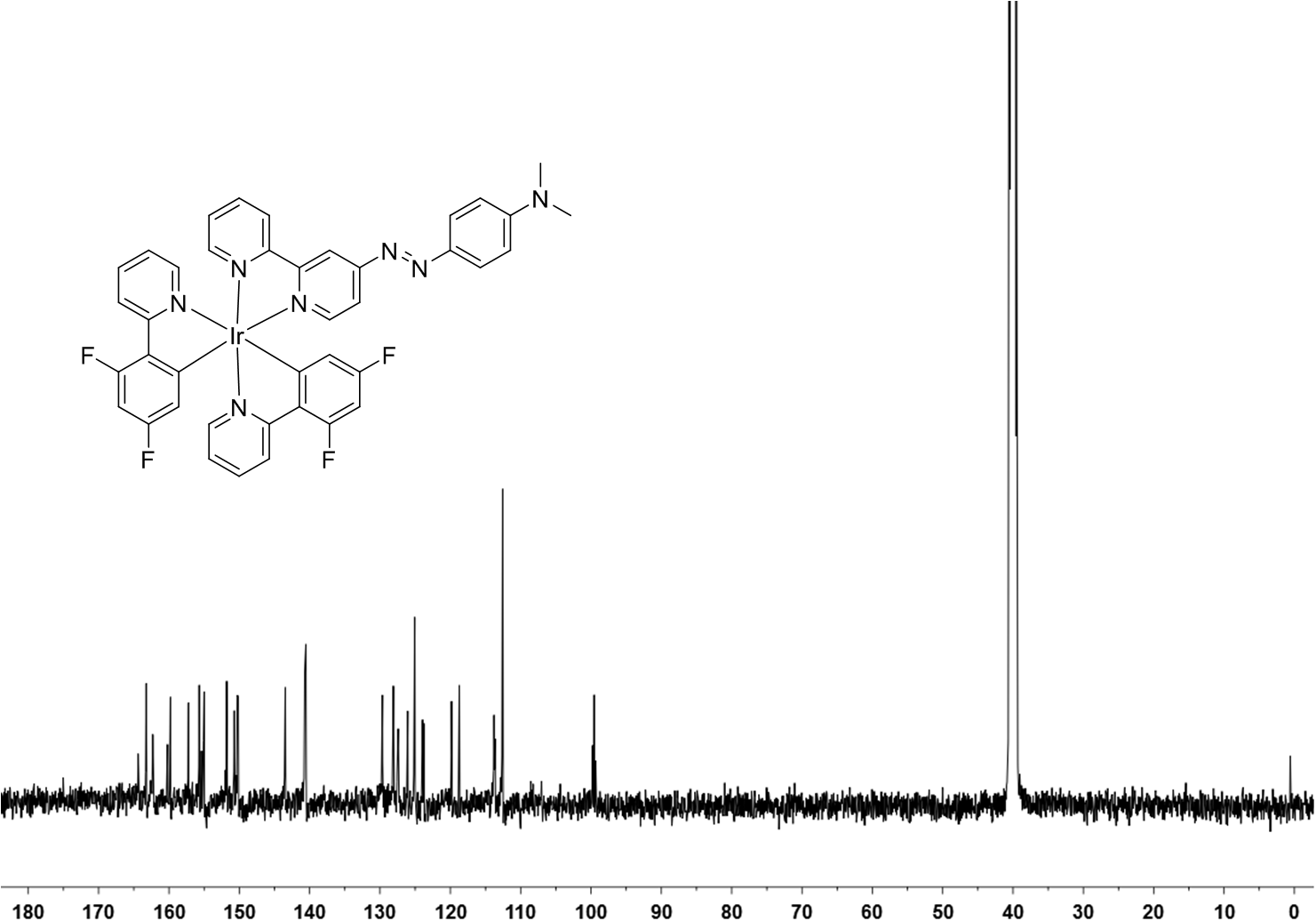


**Figure S14.** ^13^C NMR spectrum of **Ir5** in DMSO-d_6_, 126 MHz.


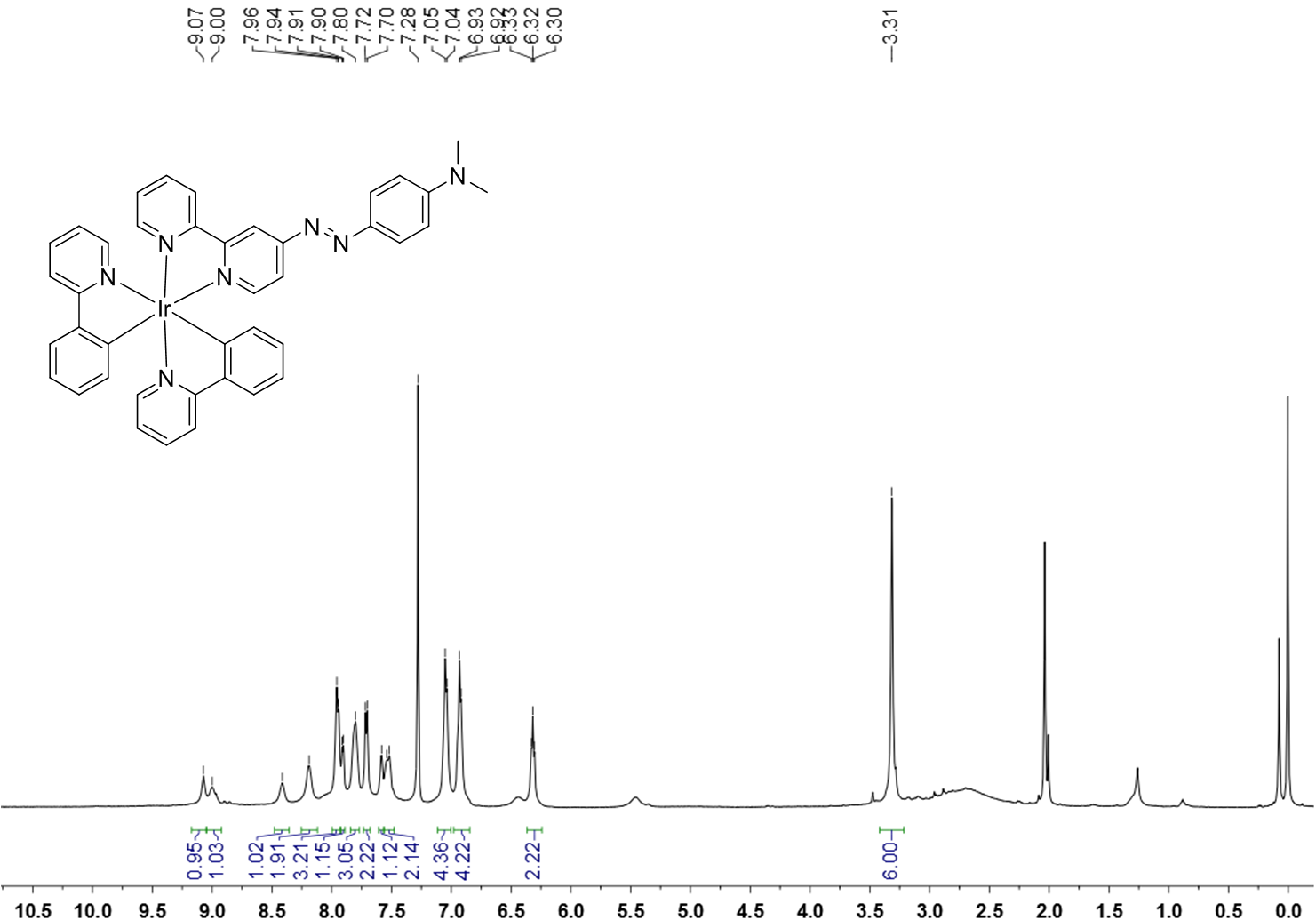


**Figure S15*.*** ^1^H NMR spectrum of **Ir6** in CDCl_3_, 500 MHz.


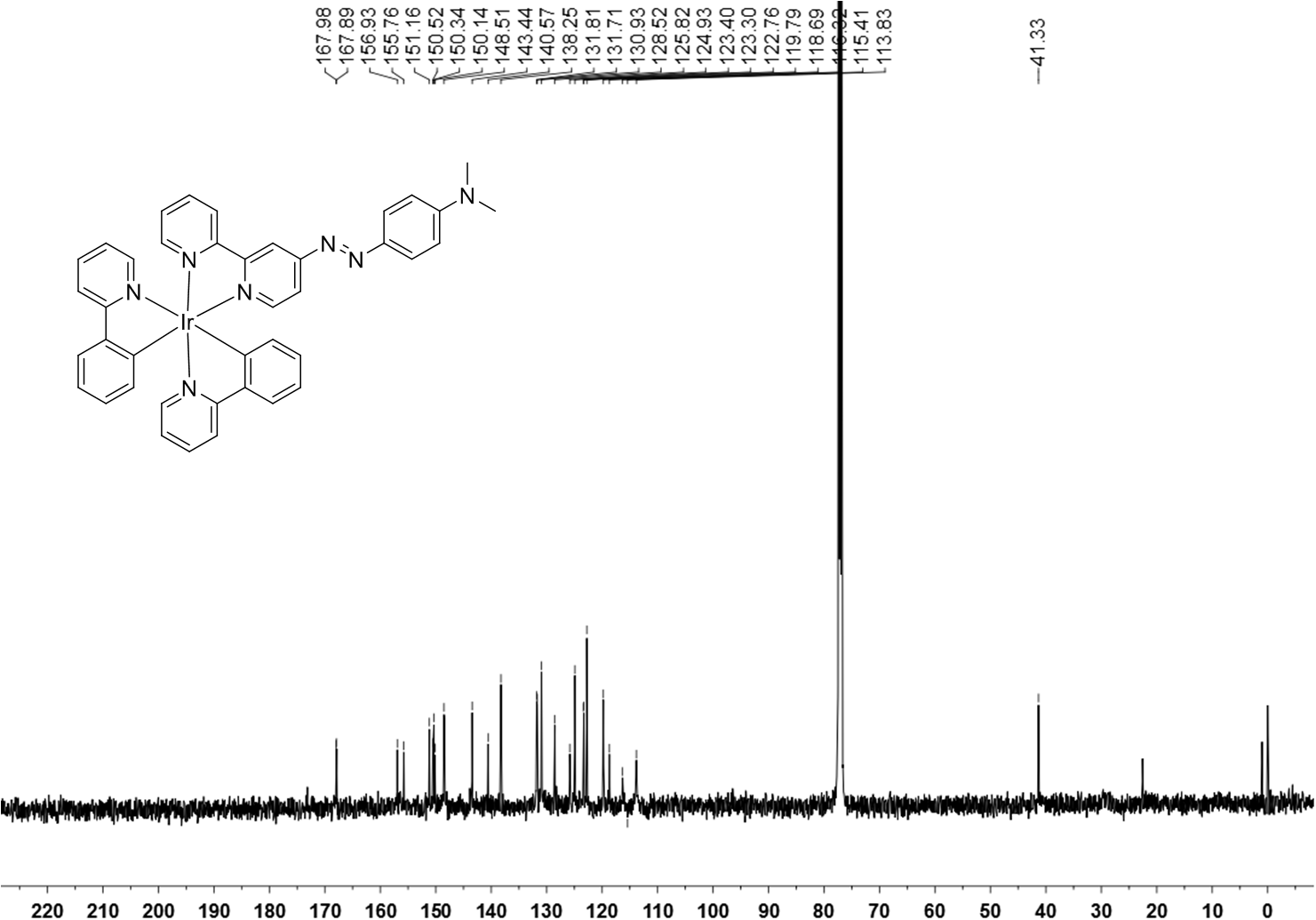


**Figure S16*.*** ^13^C NMR spectrum of **Ir6** in CDCl_3_, 126 MHz.


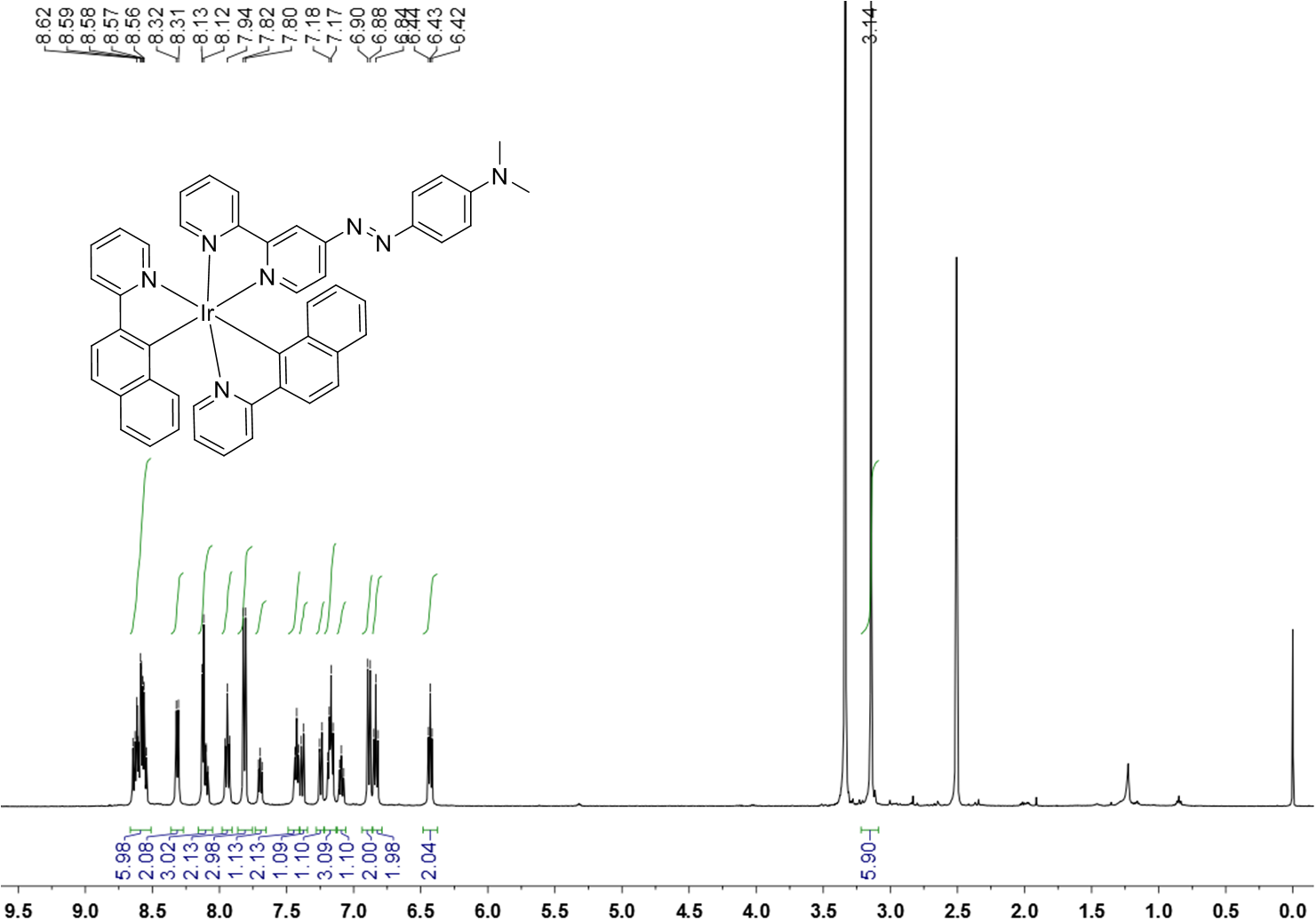


**Figure S17.** ^1^H NMR spectrum of **Ir7** in DMSO-d_6_, 500 MHz.


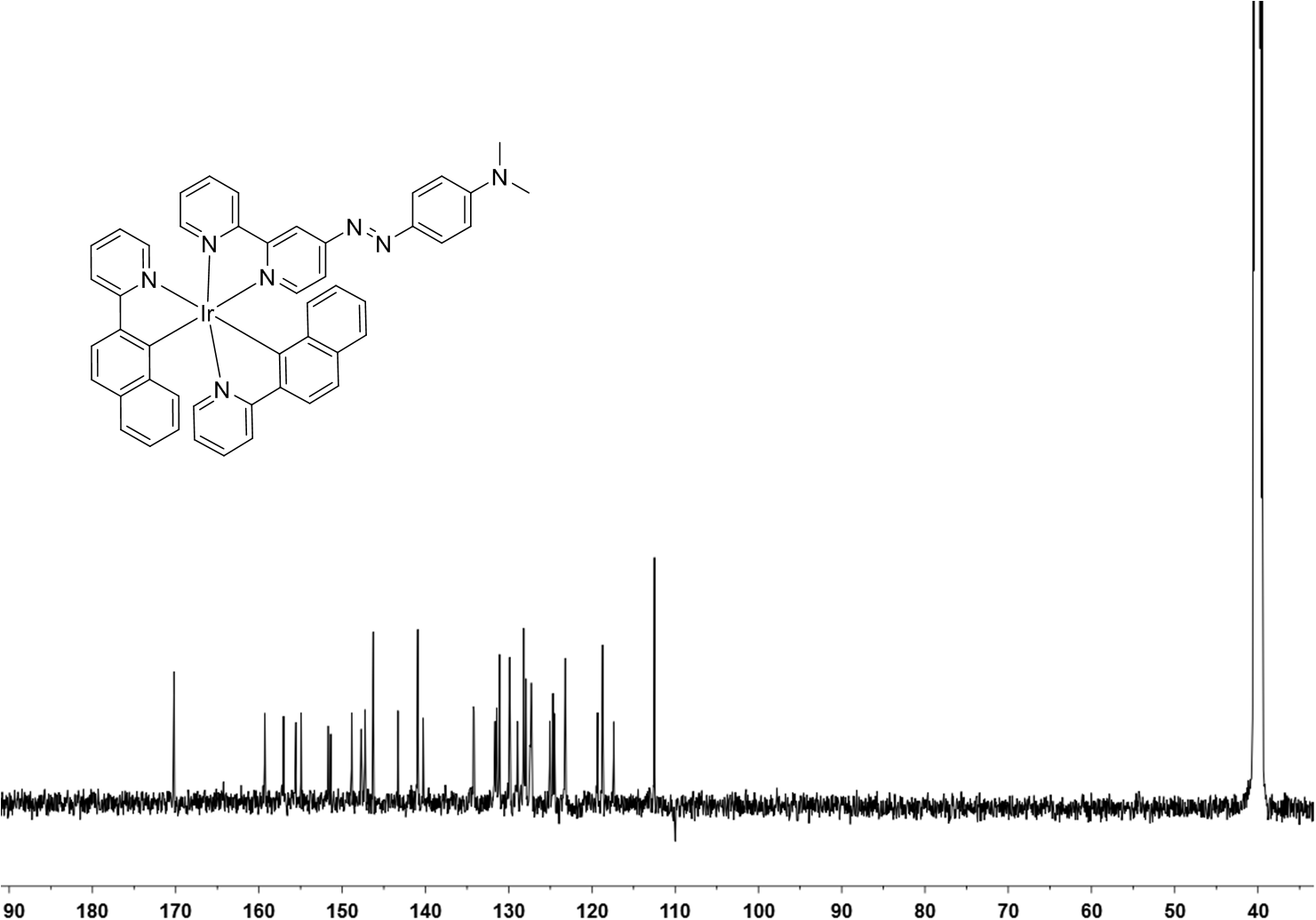


**Figure S18.** ^13^C NMR spectrum of **Ir7** in DMSO-d_6_, 126 MHz.


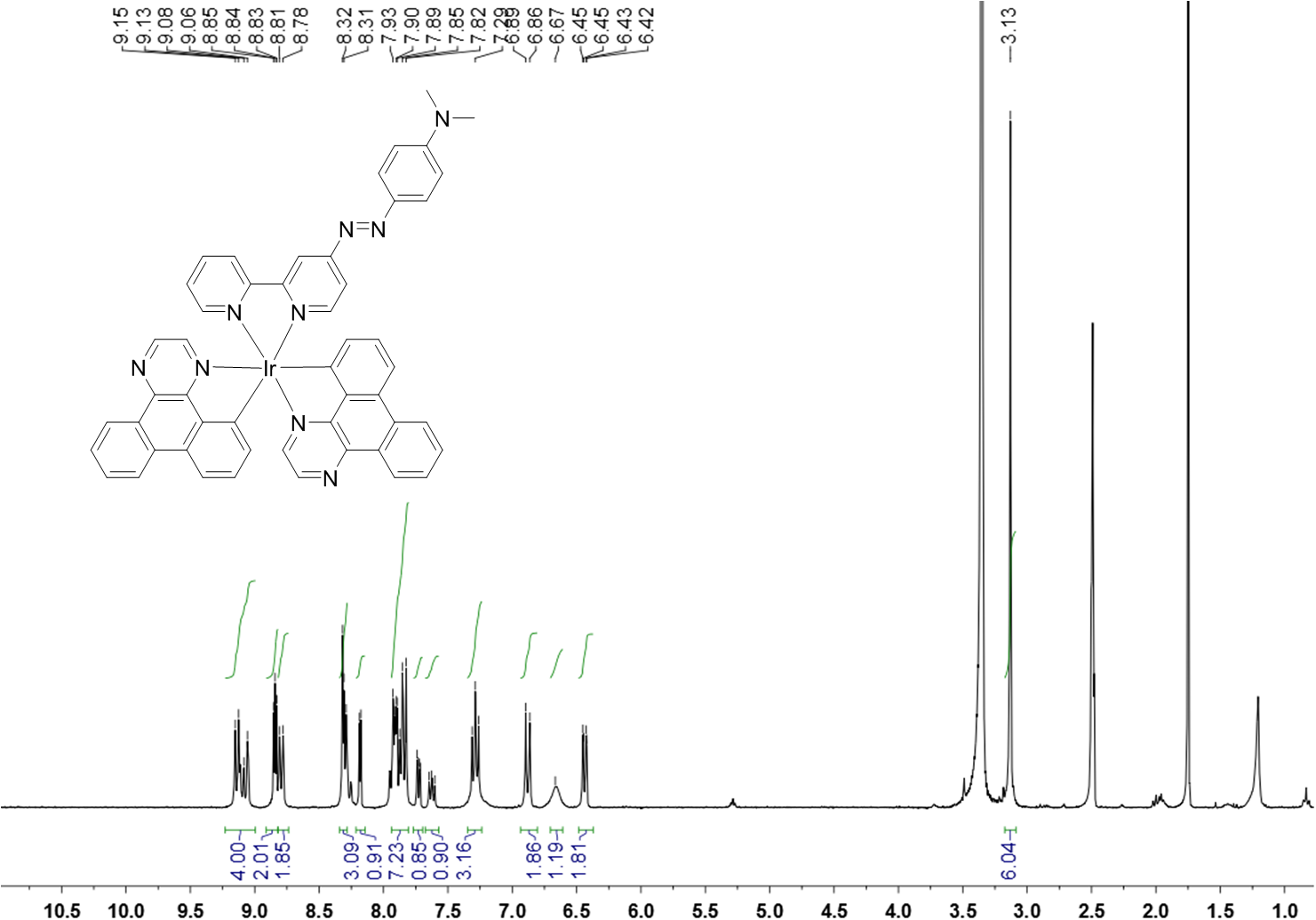


**Figure S19*.*** ^1^H NMR spectrum of **Ir8** in DMSO-d_6_, 500 MHz.


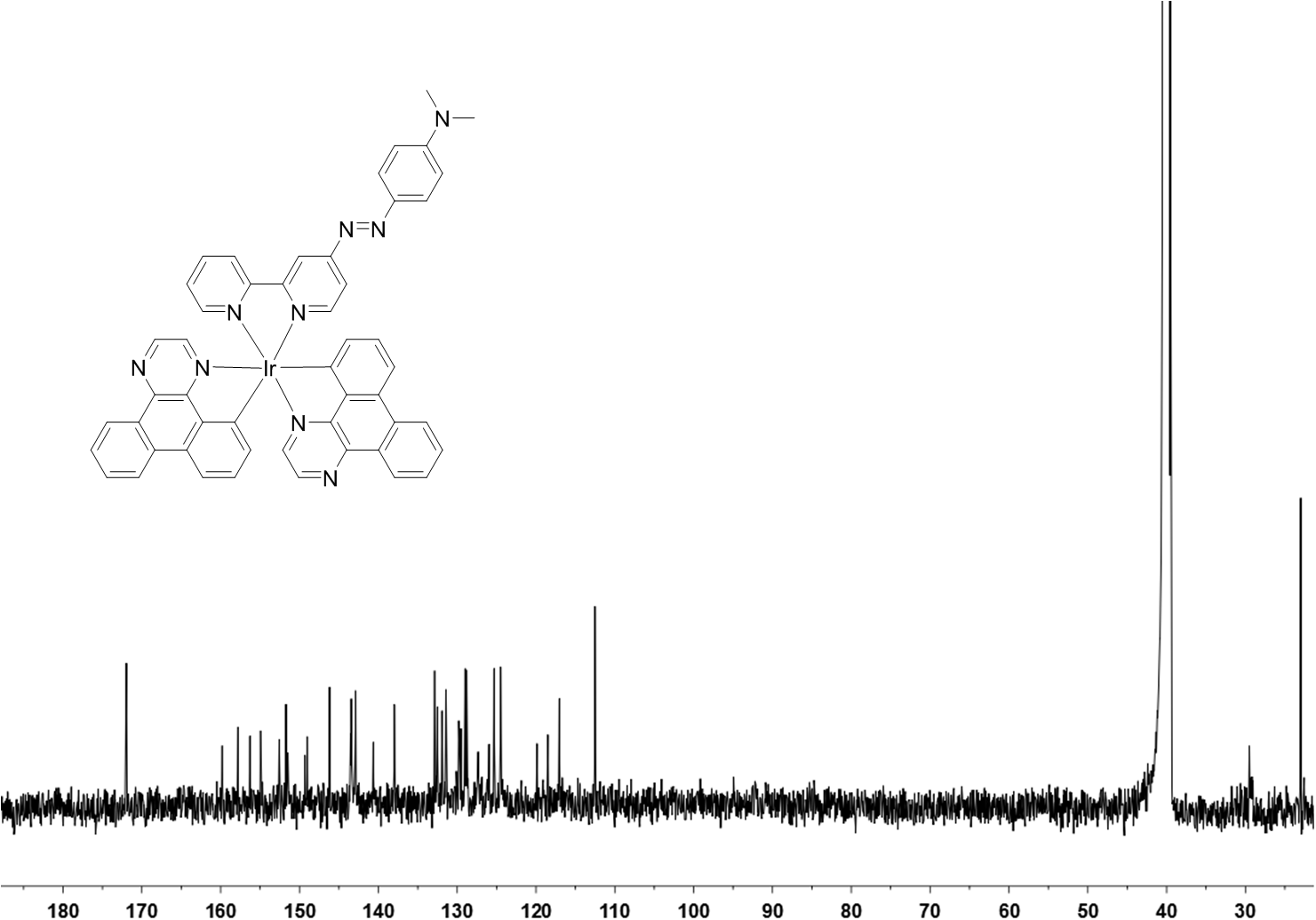


**Figure S20*.*** ^13^C NMR spectrum of **Ir8** in DMSO-d_6_, 126 MHz.


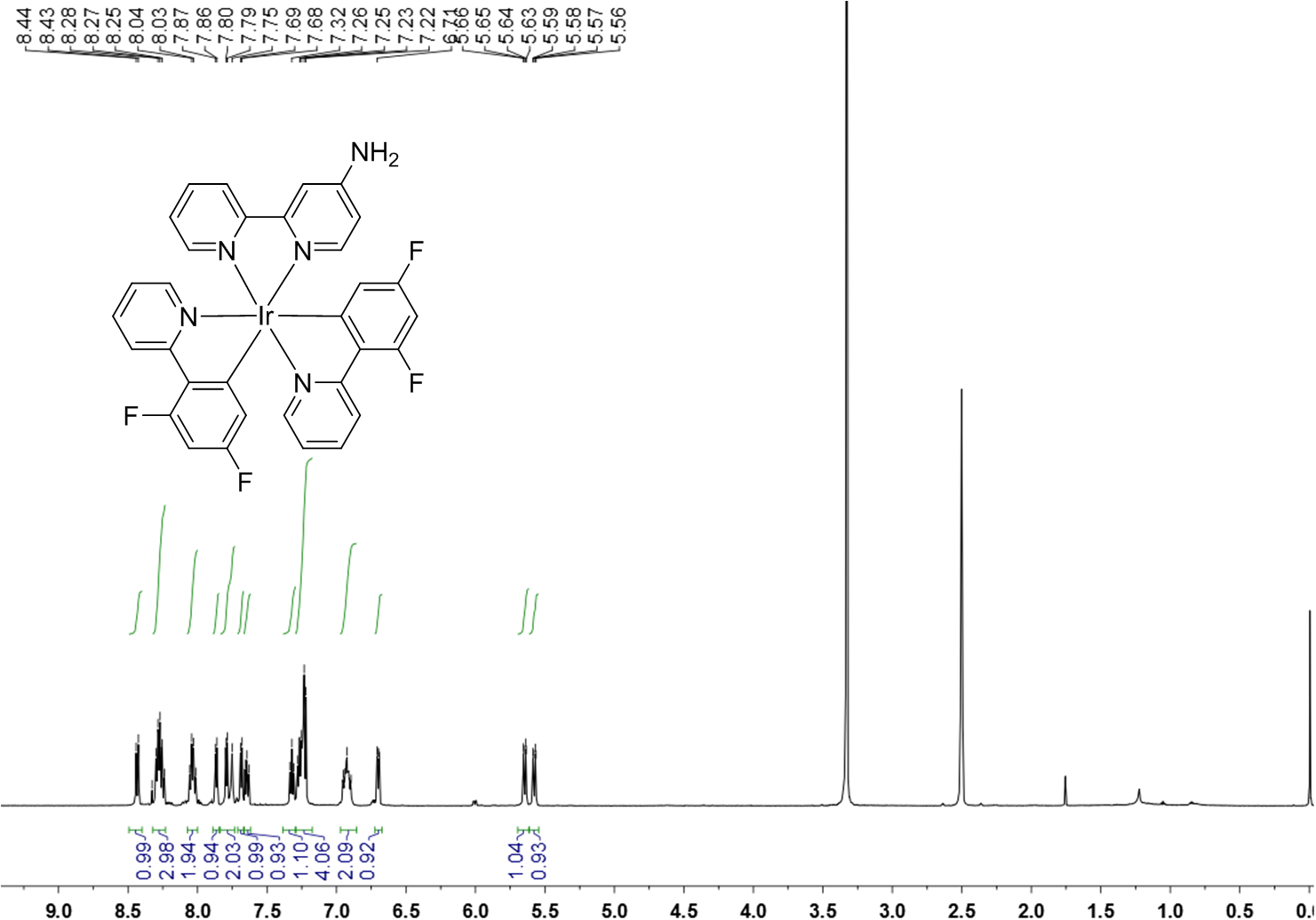


**Figure S21.** ^1^H NMR spectrum of **IrNH_2_1** in DMSO-d_6_, 500 MHz.


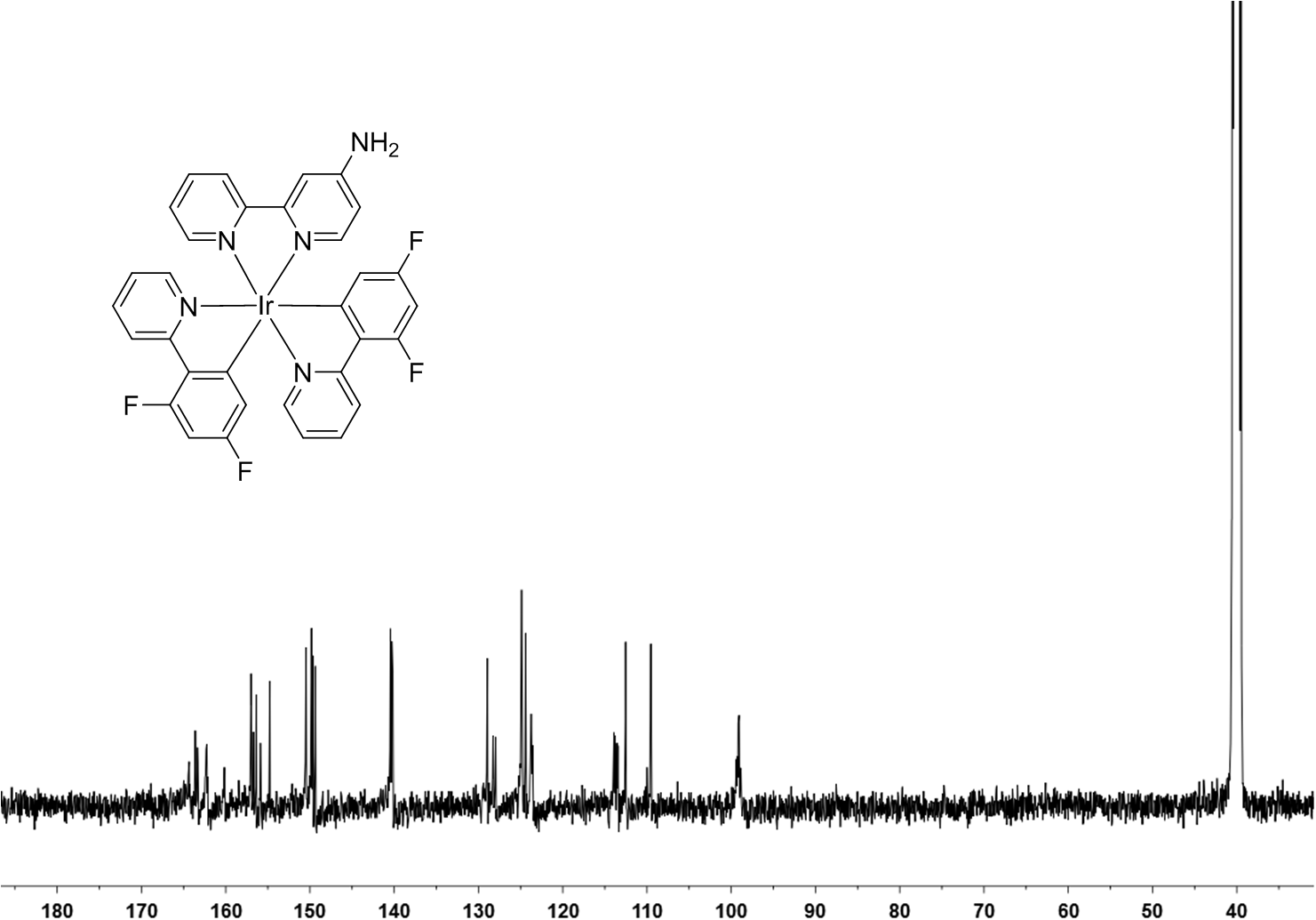


**Figure S22*.*** ^13^C NMR spectrum of **IrNH_2_1** in DMSO-d_6_, 126 MHz.


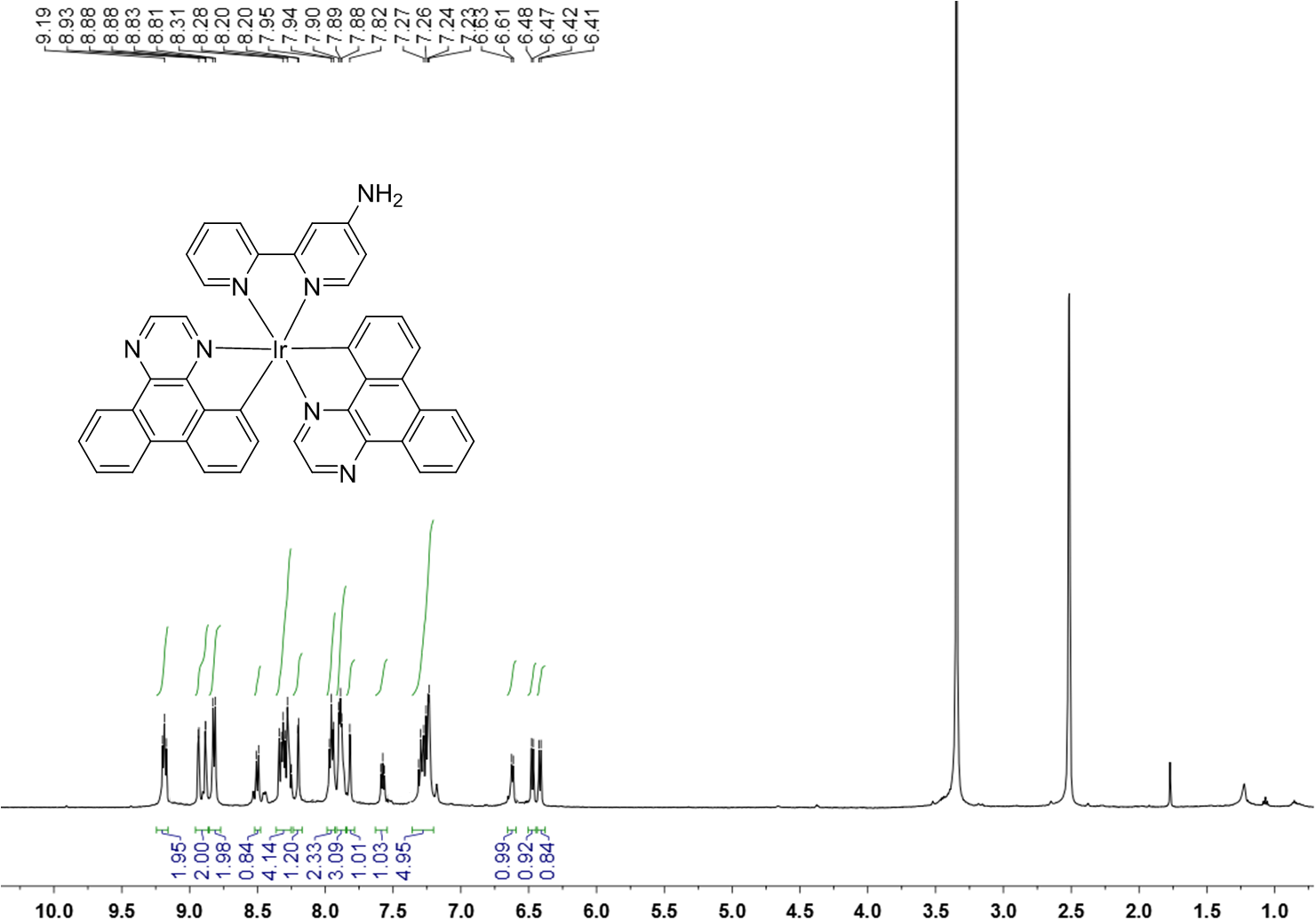


**Figure S23.** ^1^H NMR spectrum of **IrNH_2_4** in DMSO-d_6_, 500 MHz.


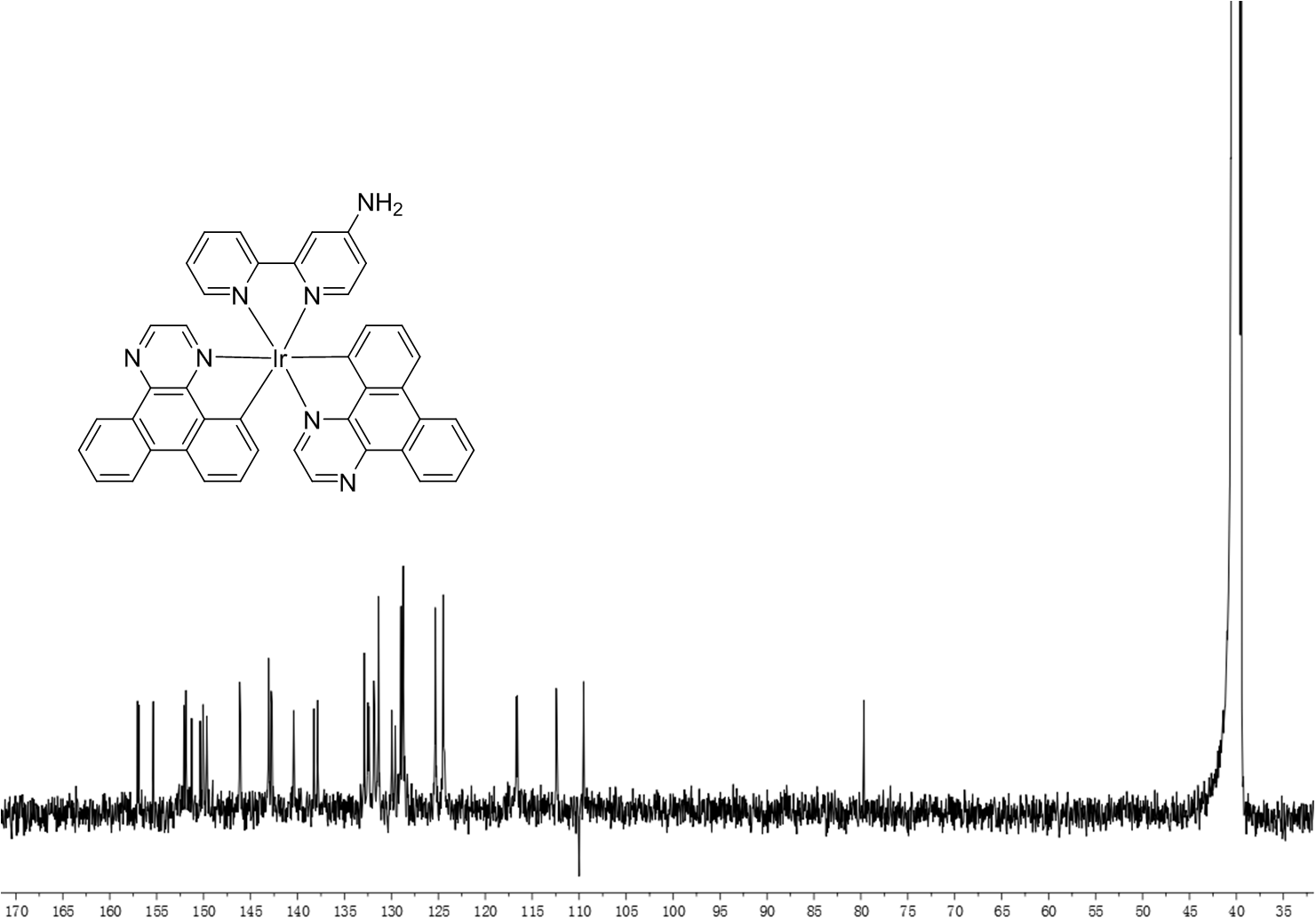


**Figure S24.** ^13^C NMR spectrum of **IrNH_2_4** in DMSO-d_6_, 126 MHz.


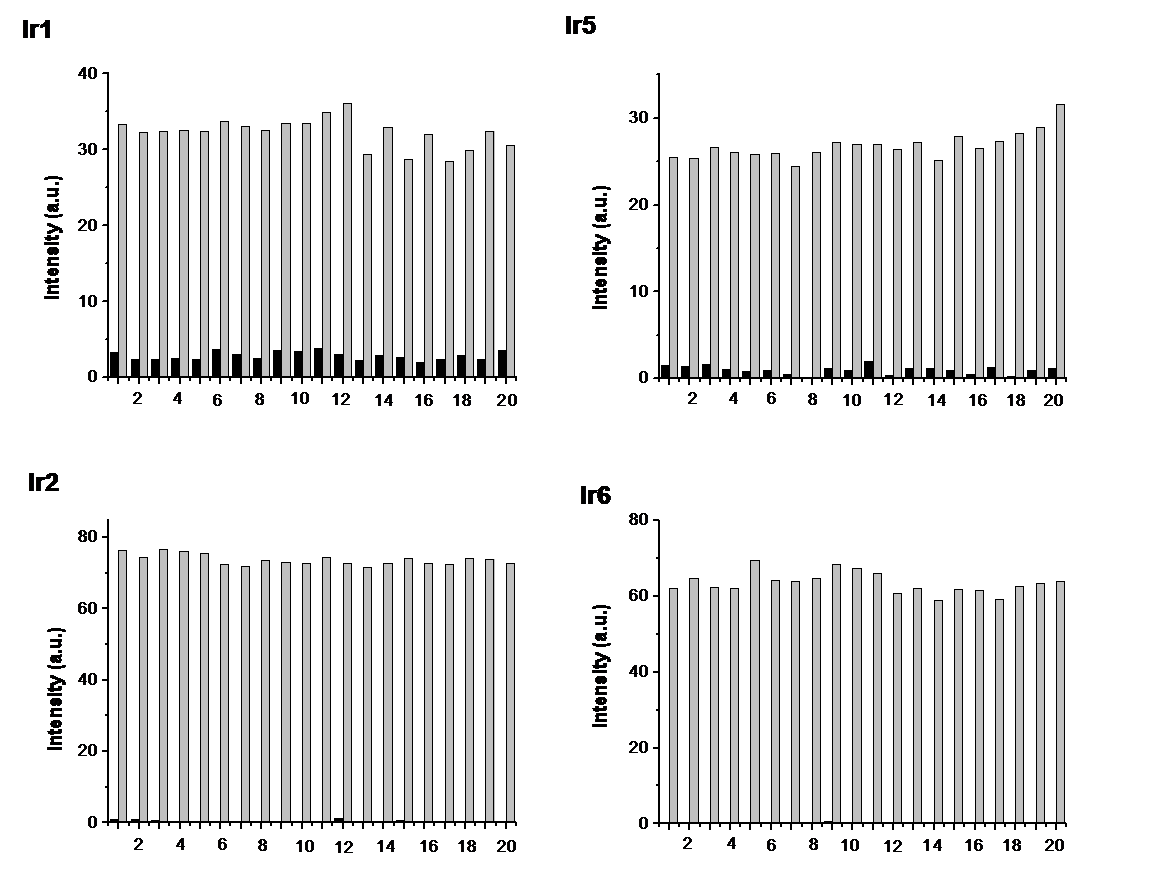

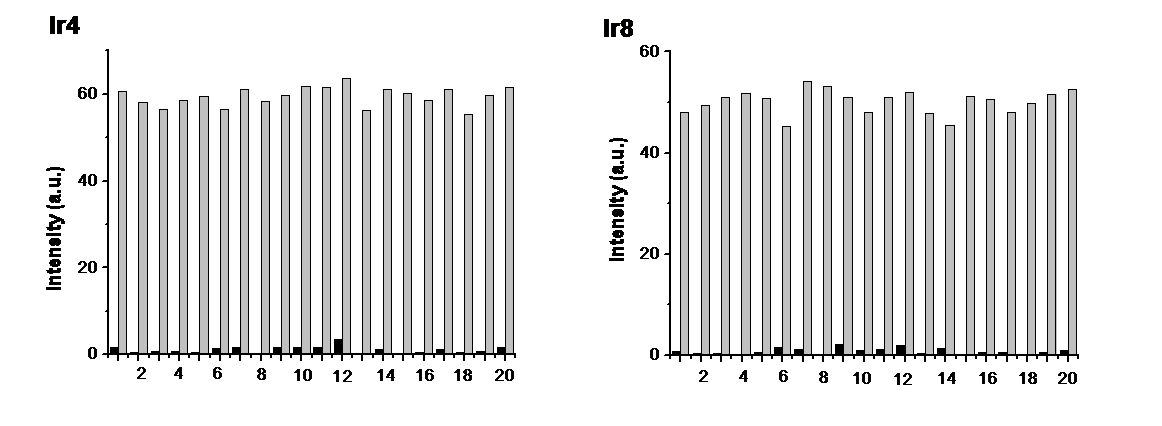
**Figure S25.** Phosphorescence intensity of 2.5 μM probes (the data of **Ir3** and **Ir7** in text) treated with different amino acids or ions (20 mM of each species) in a mixed solution of 0.1 M potassium phosphate buffer (pH 7.4) under normoxia and hypoxia environment. Left-hand bars represent the luminescent responses toward amino acids or ions under normoxia environment, right-hand bars represent the subsequent degassed with argon gas and addition of rat liver microsomes (0.25 mg protein/mL) and 50 μM NADPH as a cofactor for reductases for 30 min at 37 °C. Bar from (1) to (20): Cys, cystine, GSH, sodium ascorbate, H_2_O_2_, Mg^2+^, Cu^2+^, Zn^2+^, Co^2+^, Ca^2+^, SO_4_^2-^, HCO_3_^-^, NO_2_^-^, NO_3_^-^, SO_3_^2-^, I^-^, ClO_4_^-^, ClO^-^, S^2-^, CO_3_^2-^. **Ir1** and **Ir5**: λ_ex/em_ = 405/510 nm; **Ir2** and **Ir6**: λ_ex/em_ = 405/570 nm; **Ir4** and **Ir8**: λ_ex/em_ = 405/640 nm.


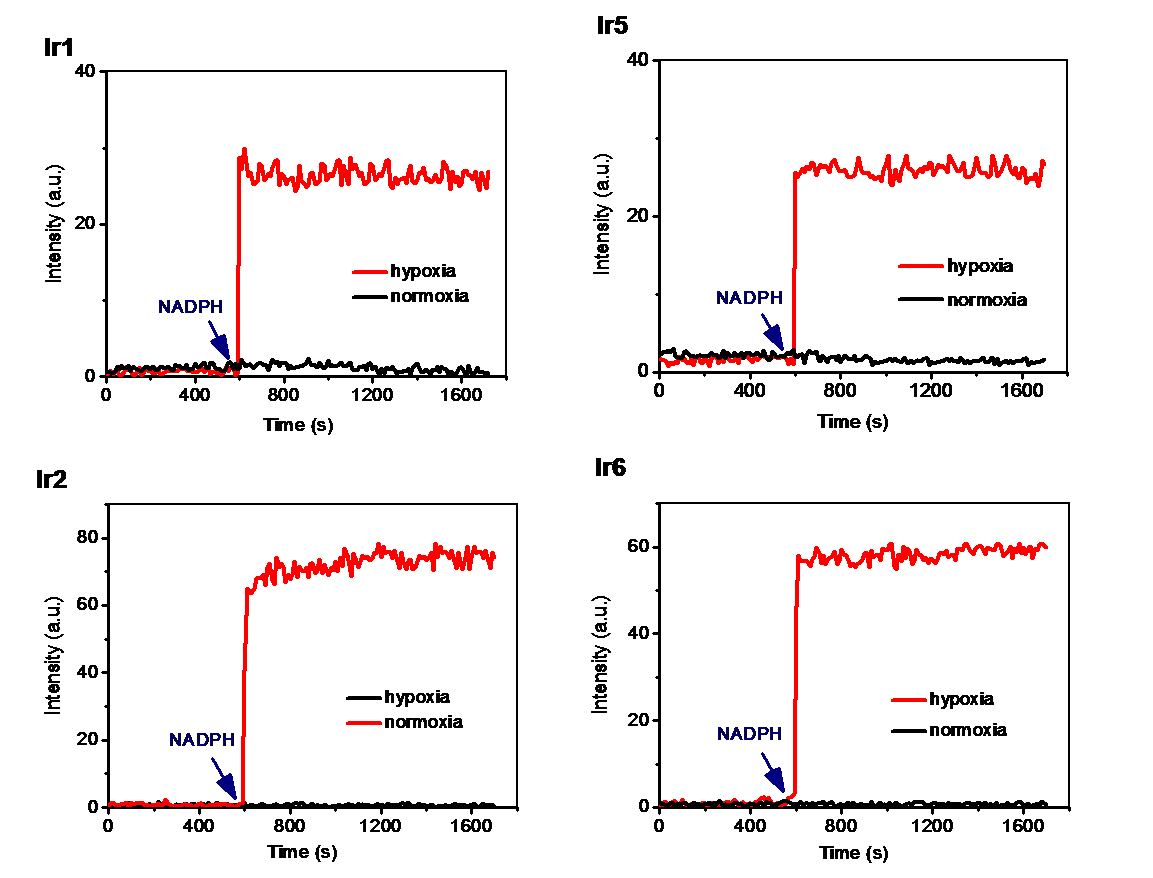

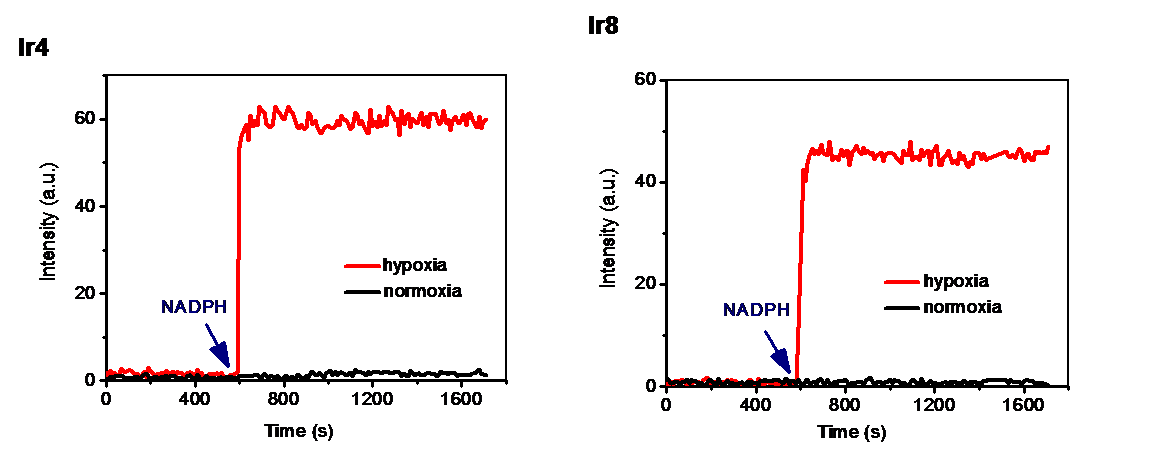
**Figure S26.** Time-dependent changes of Phosphorescence intensity of 2.5 μM probes (the data of **Ir3** and **Ir7** in text) under normoxic and hypoxic conditions. Data were measured at 37 °C in 0.1 M potassium phosphate buffer (pH 7.4) containing rat liver microsomes (0.25 mg protein/mL), 50 μM NADPH as a coenzyme, and 0.1% DMSO as a cosolvent. NADPH was added at the point indicated by the arrowhead. **Ir1** and **Ir5**: λ_ex/em_ = 405/510 nm; **Ir2** and **Ir6**: λ_ex/em_ = 405/570 nm; **Ir4** and **Ir8**: λ_ex/em_ = 405/640 nm.


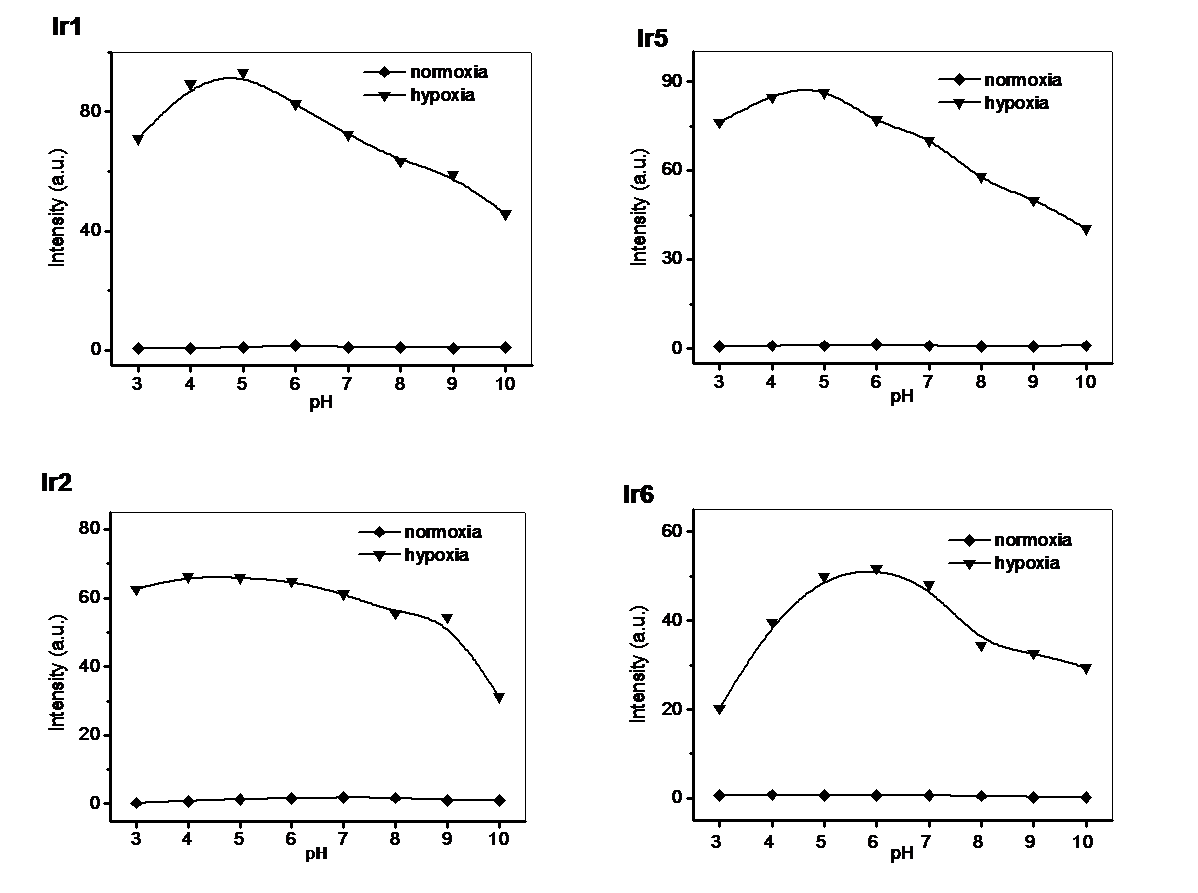

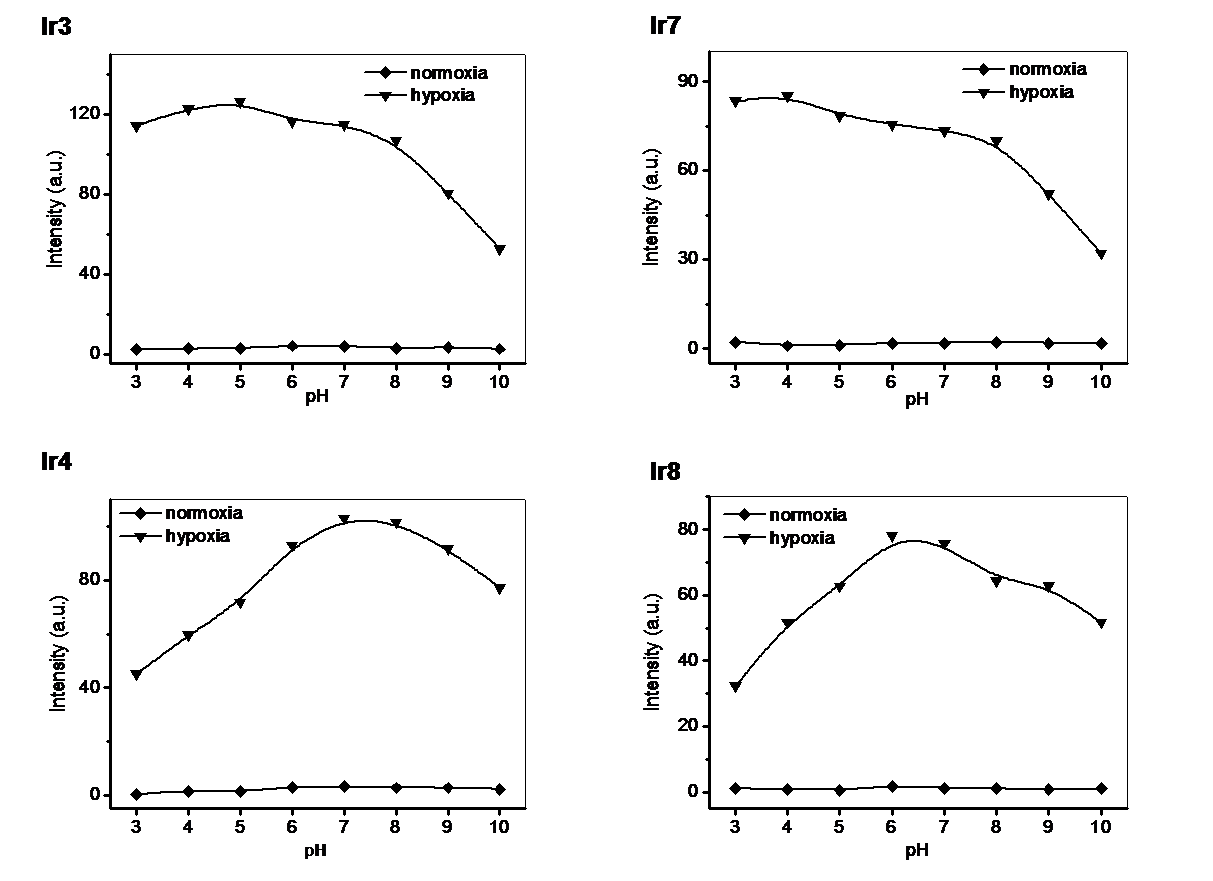


**Figure S27.** The changes in phosphorescence intensity of **Ir1-Ir8** (2.5 μM) in 0.1 M sodium phosphate buffer at various pH values. **Ir1** and **Ir5**: λ_ex/em_ = 405/510 nm; **Ir2** and **Ir6**: λ_ex/em_ = 405/570 nm; **Ir3** and **Ir7**: λ_ex/em_ = 405/580 nm; **Ir4** and **Ir8**: λ_ex/em_ = 405/640 nm.


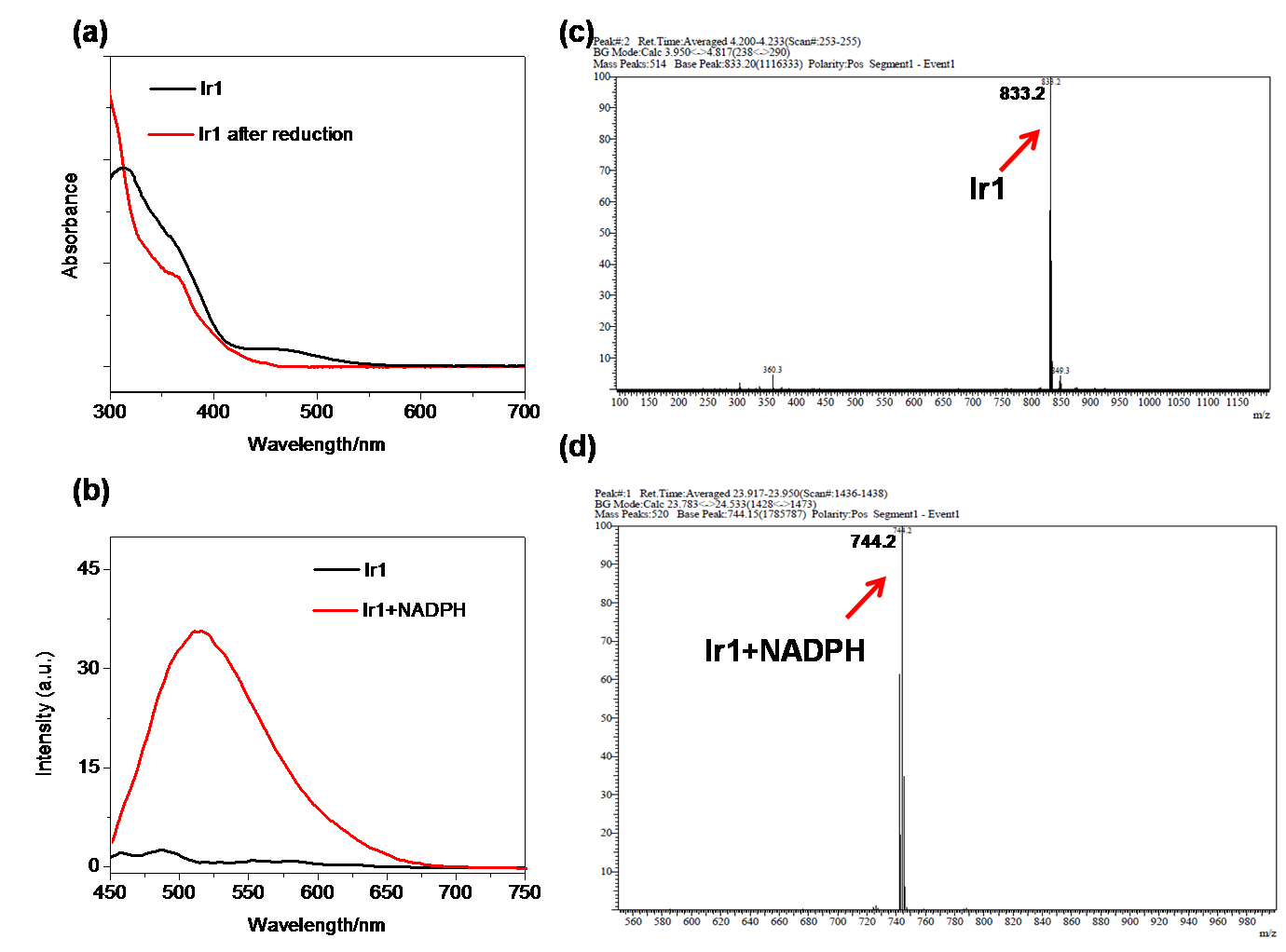

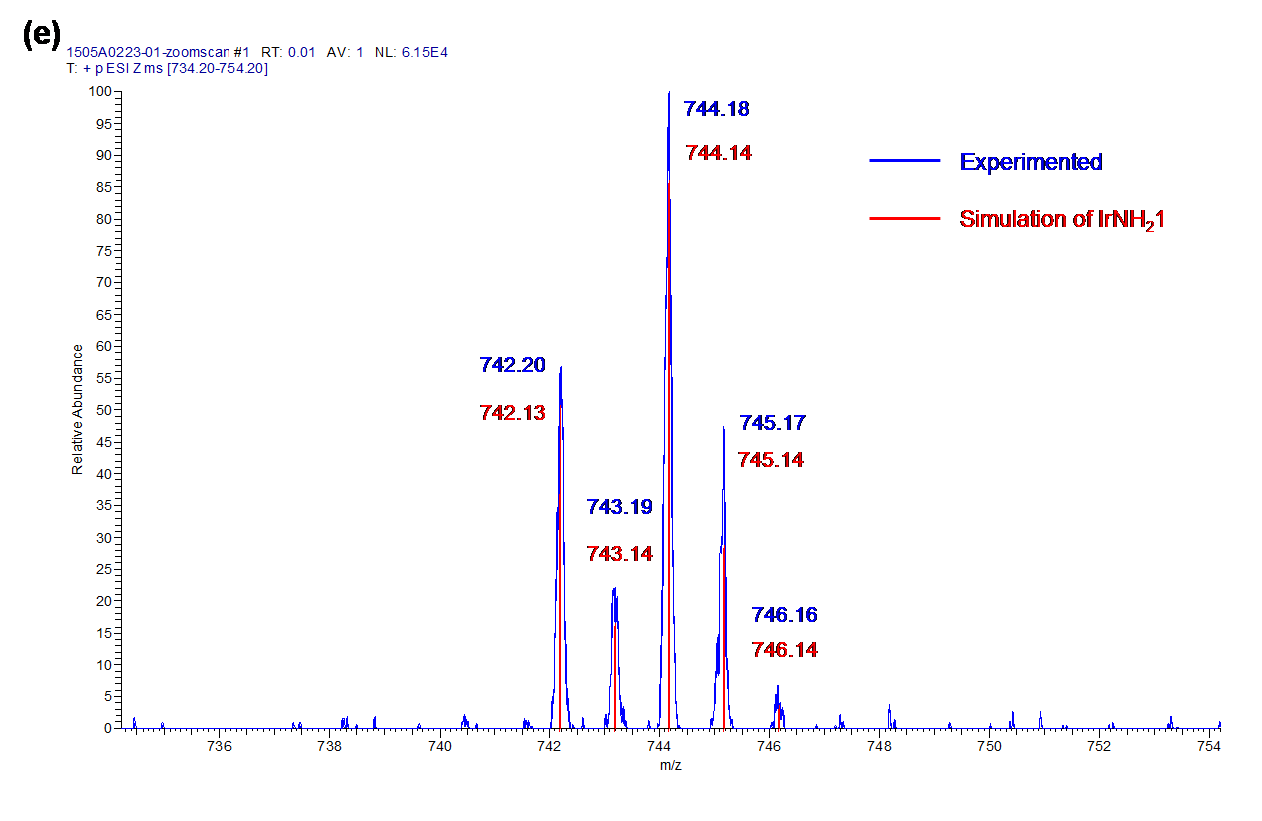


**Figure S28. (a):** Spectral changes of **Ir1** in the presence of rat liver microsomes (0.25 mg protein/mL) under normoxic (black) or hypoxic (red) conditions. **(b):** Phosphorescence spectrum of the reaction mixture generated in the hypoxic metabolism of **Ir1** by NADPH and rat liver microsomes. (red line, with emission maxima at 580 nm). **(c)-(e):** ESI-MS analysis of the reaction mixture generated by **Ir1**; **(c):** ESI-MS spectrum of **Ir1**. **(d):** ESI-MS spectrum of the product of **Ir1** after incubation with rat liver microsomes (0.25 mg protein/mL) and 50 μM NADPH at 37 °C for 30 min. **(e):** ESI-MS spectrum of the reduced product of **Ir1** and the simulation of **IrNH_2_1**.


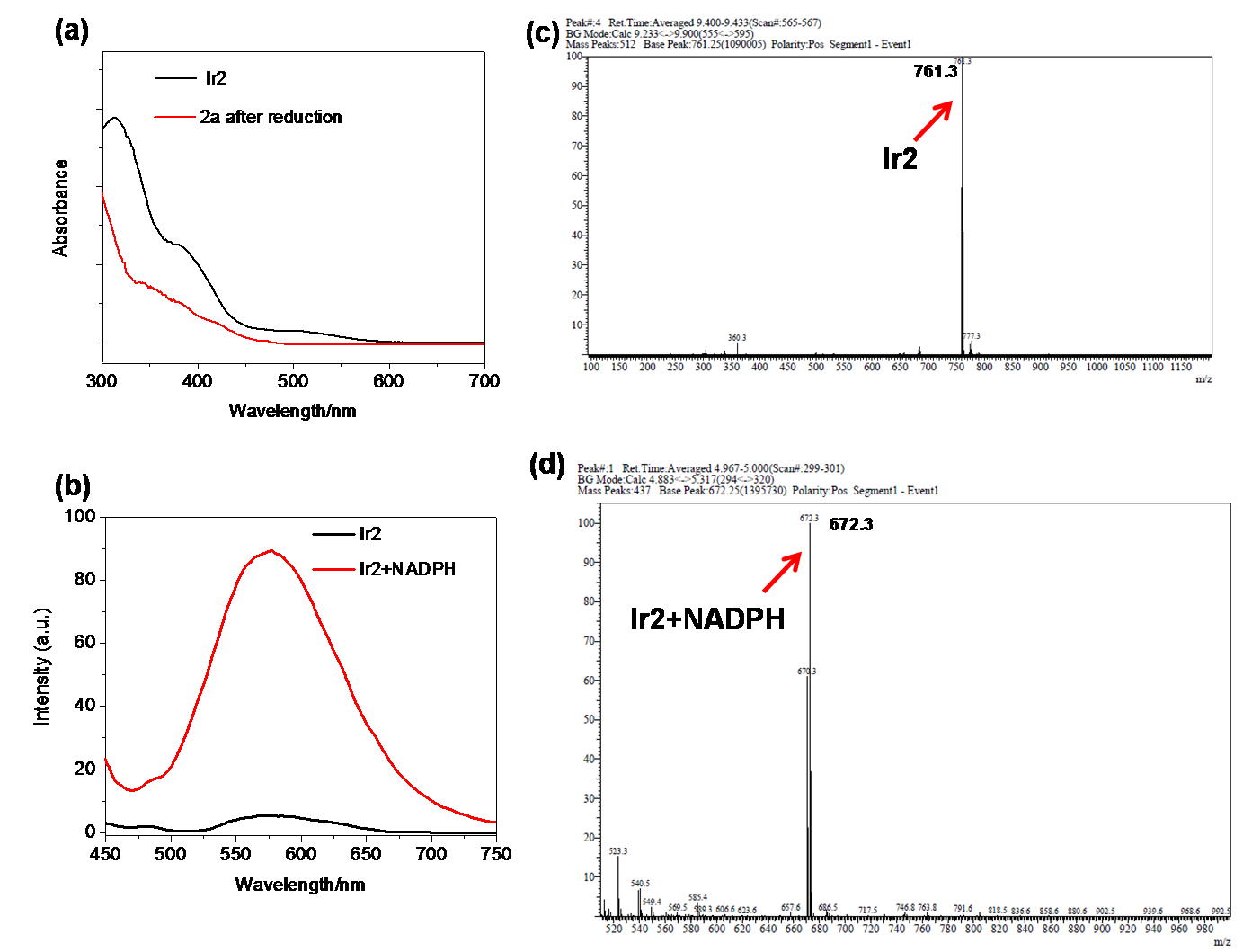

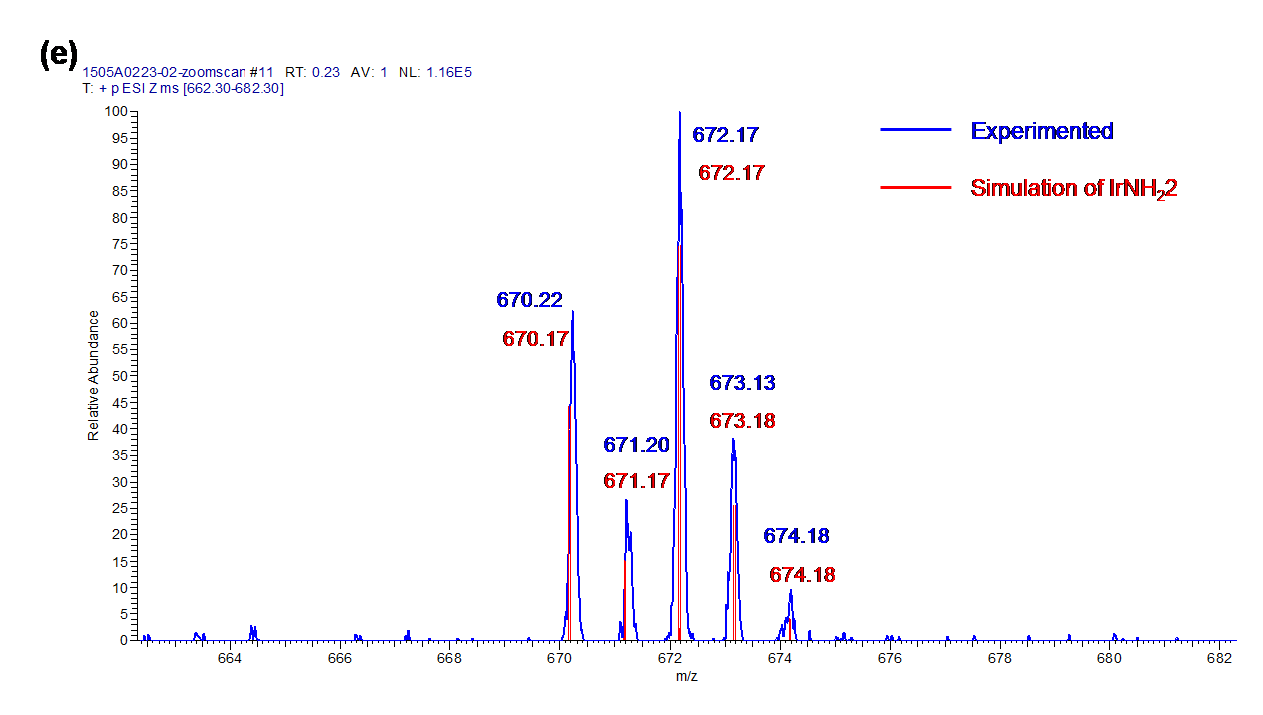
 **Figure S29. (a):** Spectral changes of **Ir2** in the presence of rat liver microsomes (0.25 mg protein/mL) under normoxic (black) or hypoxic (red) conditions. **(b):** Phosphorescence spectrum of the reaction mixture generated in the hypoxic metabolism of **Ir2** by NADPH and rat liver microsomes. (red line, with emission maxima at 580 nm). **(c)-(e):** ESI-MS analysis of the reaction mixture generated by **Ir2**; **(c):** ESI-MS spectrum of **Ir2**. **(d):** ESI-MS spectrum of the product of **Ir2** after incubation with rat liver microsomes (0.25 mg protein/mL) and 50 μM NADPH at 37 °C for 30 min. **(e):** ESI-MS spectrum of the reduced product of **Ir2** and the simulation of **IrNH_2_2**.


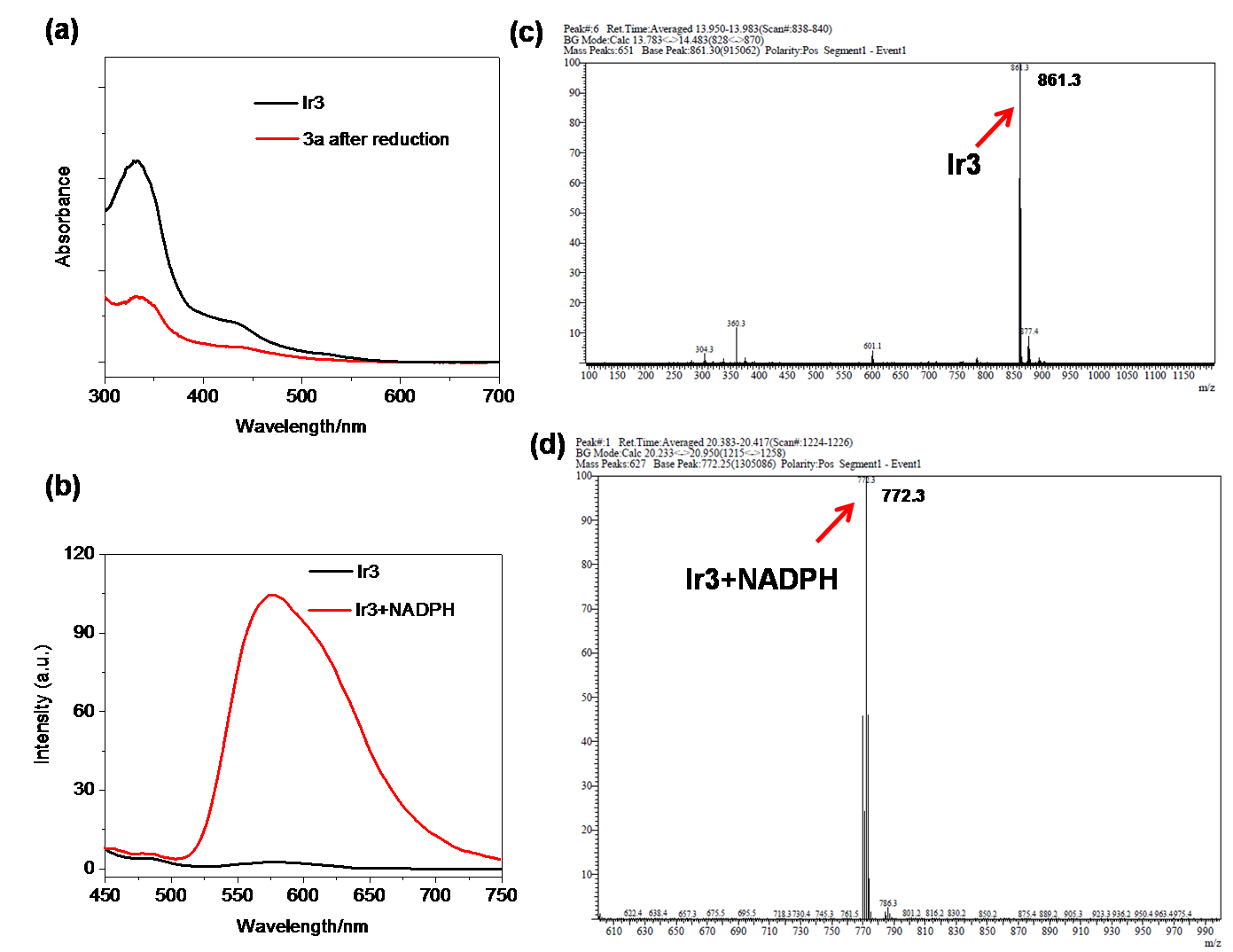

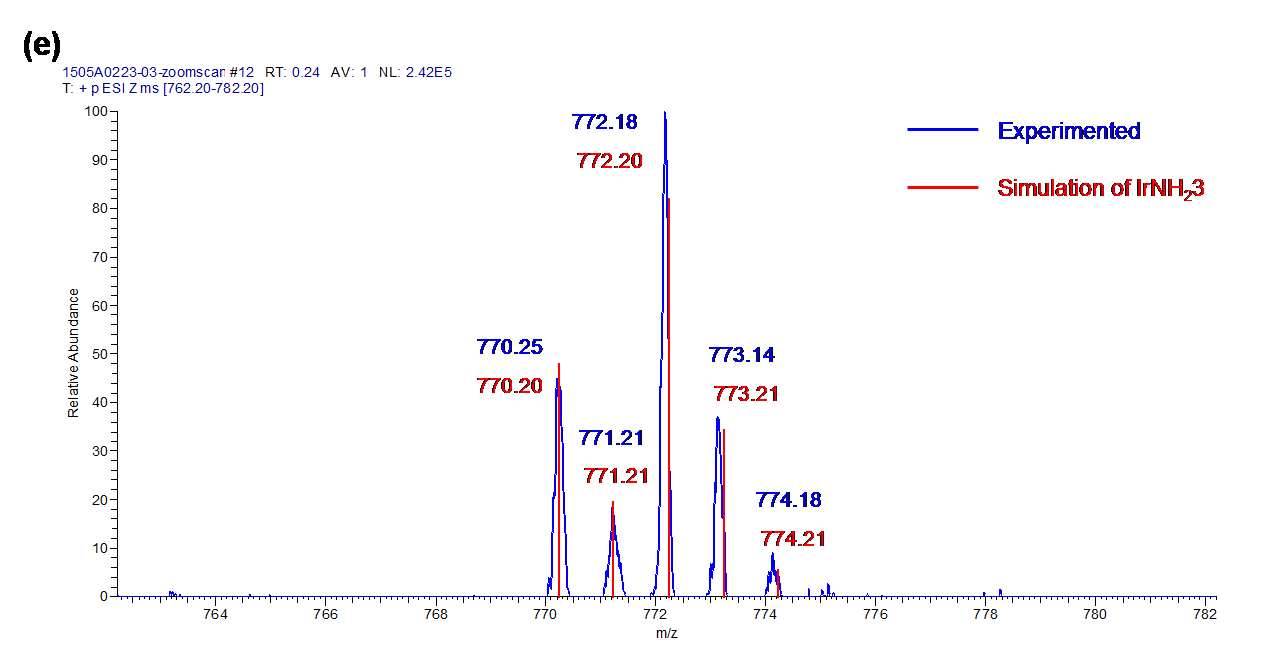
**Figure S30. (a):** Spectral changes of **Ir3** in the presence of rat liver microsomes (0.25 mg protein/mL) under normoxic (black) or hypoxic (red) conditions. **(b):** Phosphorescence spectrum of the reaction mixture generated in the hypoxic metabolism of **Ir3** by NADPH and rat liver microsomes. (red line, with emission maxima at 580 nm). **(c)-(e):** ESI-MS analysis of the reaction mixture generated by **Ir3**; **(c):** ESI-MS spectrum of **Ir3**. **(d):** ESI-MS spectrum of the product of **Ir3** after incubation with rat liver microsomes (0.25 mg protein/mL) and 50 μM NADPH at 37 °C for 30 min. **(e):** ESI-MS spectrum of the reduced product of **Ir3** and the simulation of **IrNH_2_3**.


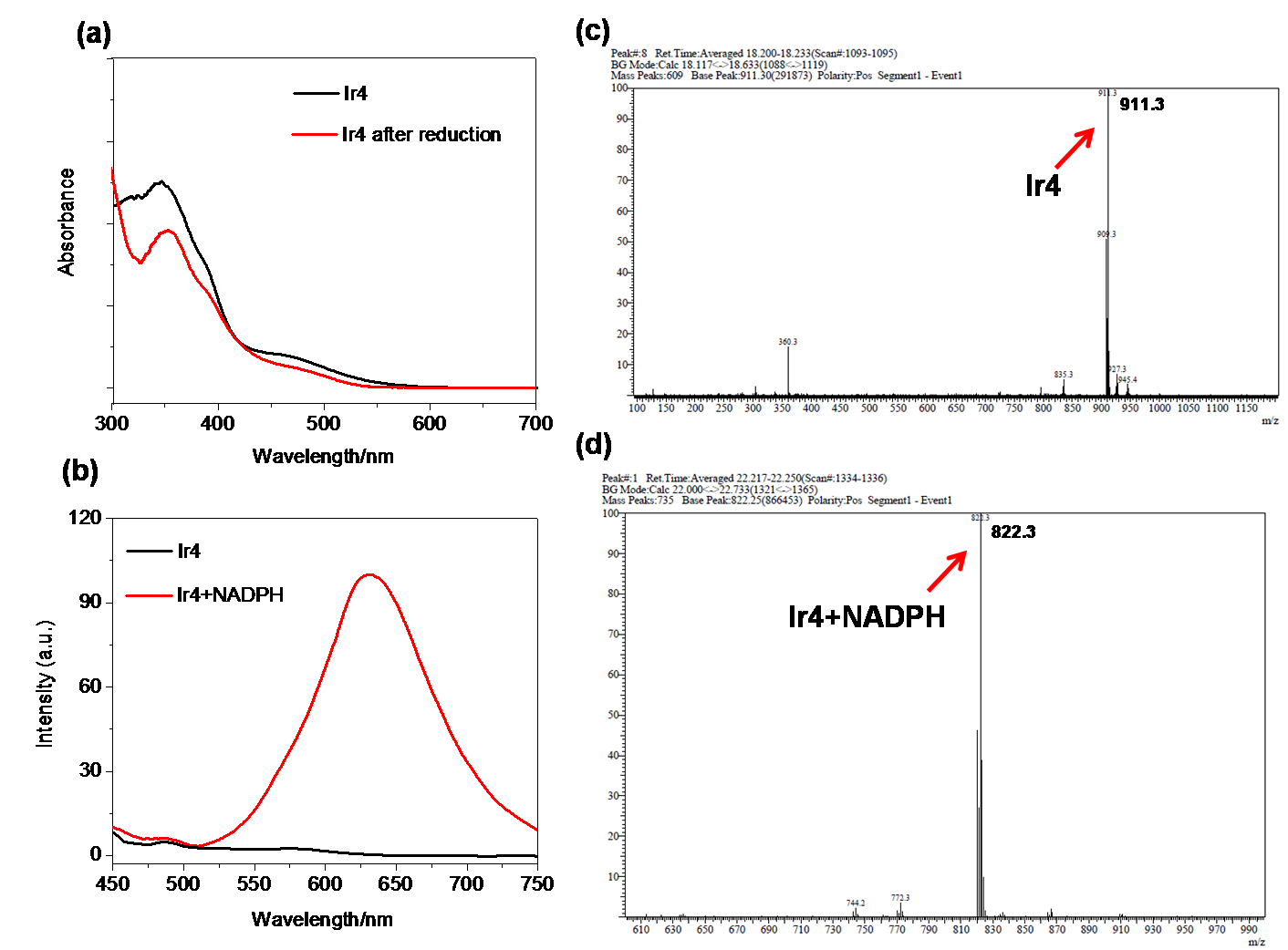

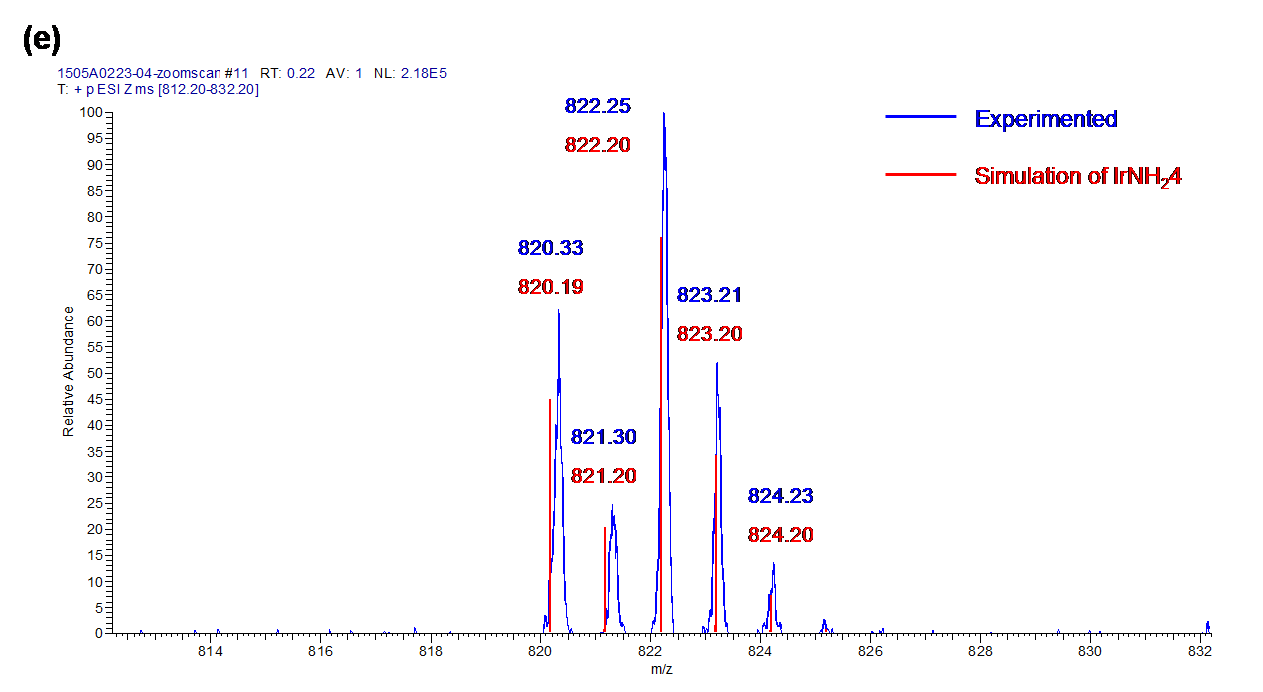
**Figure S31. (a):** Spectral changes of **Ir4** in the presence of rat liver microsomes (0.25 mg protein/mL) under normoxic (black) or hypoxic (red) conditions. **(b):** Phosphorescence spectrum of the reaction mixture generated in the hypoxic metabolism of **Ir4** by NADPH and rat liver microsomes. (red line, with emission maxima at 580 nm). **(c)-(e):** ESI-MS analysis of the reaction mixture generated by **Ir4**; **(c):** ESI-MS spectrum of **Ir4**. **(d):** ESI-MS spectrum of the product of **Ir4** after incubation with rat liver microsomes (0.25 mg protein/mL) and 50 μM NADPH at 37 °C for 30 min. **(e):** ESI-MS spectrum of the reduced product of **Ir4** and the simulation of **IrNH_2_4**.


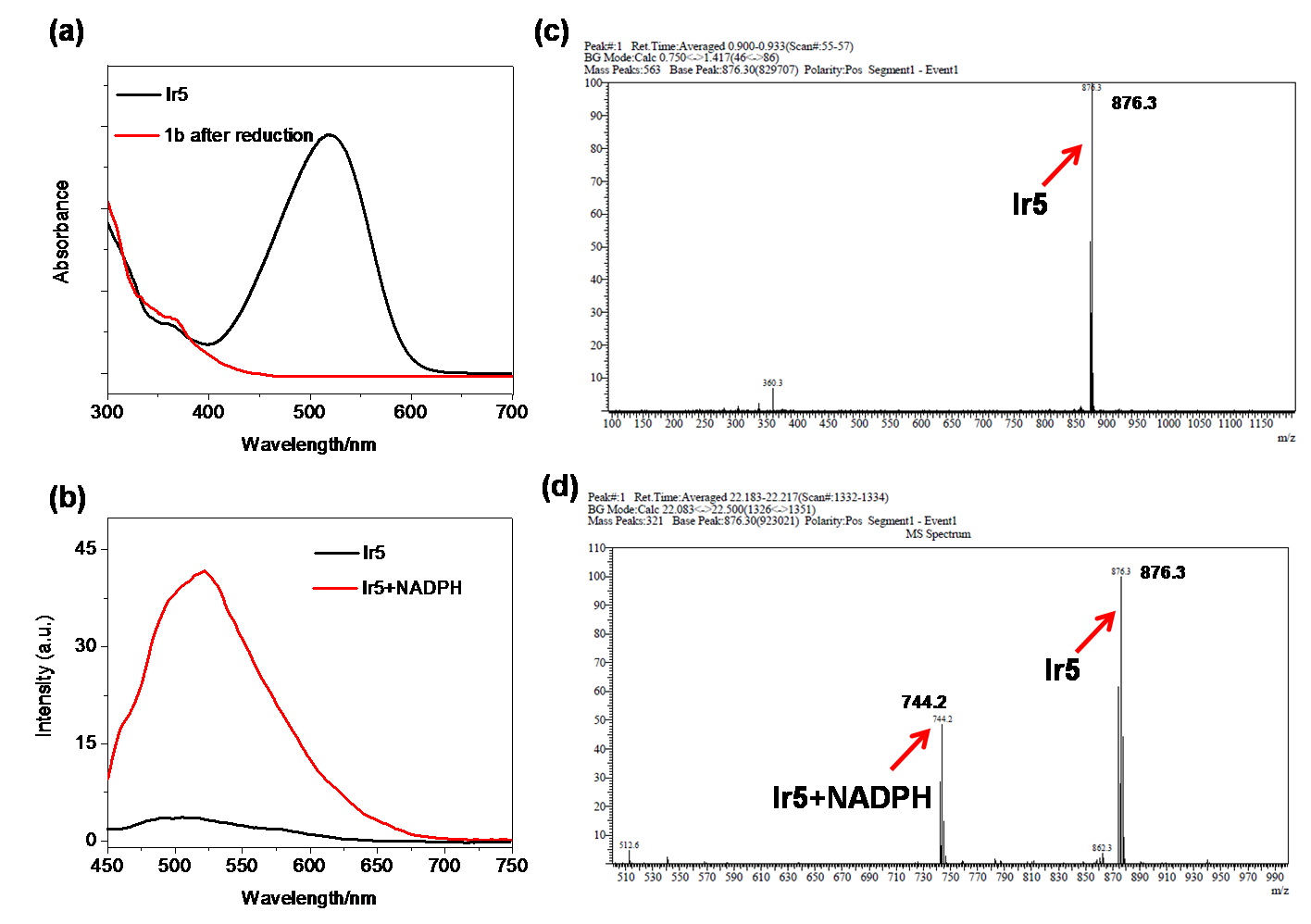

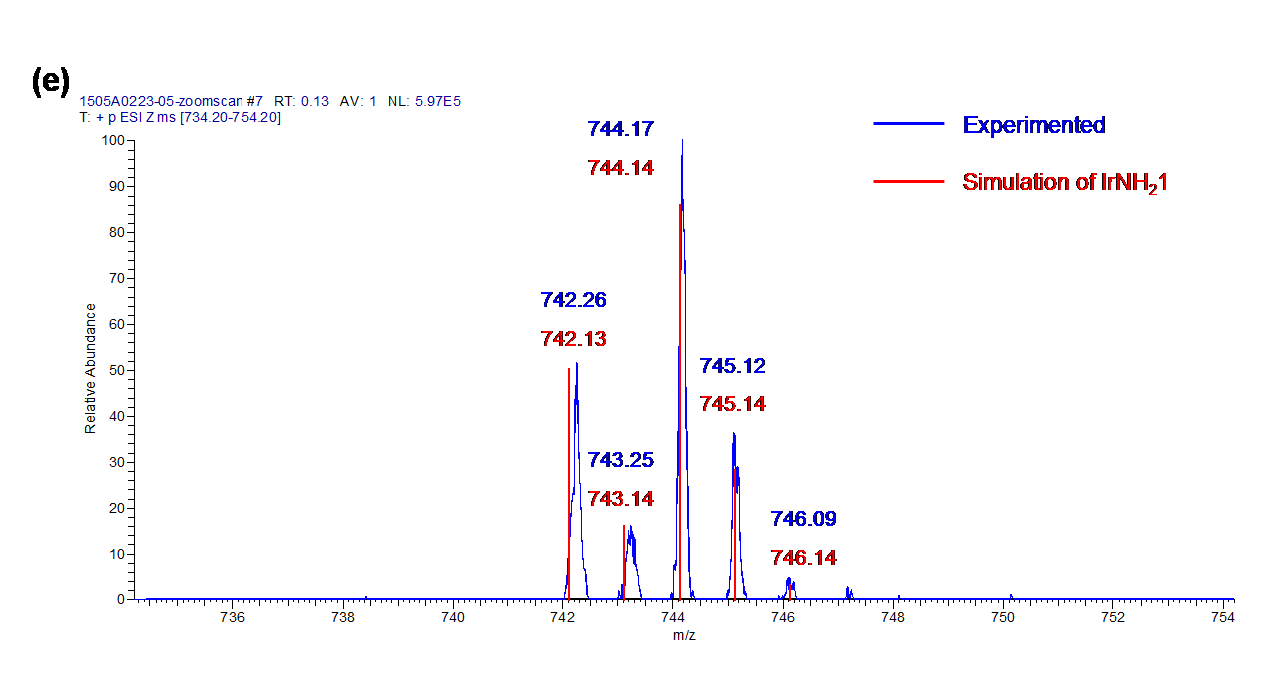
**Figure S32. (a):** Spectral changes of **Ir5** in the presence of rat liver microsomes (0.25 mg protein/mL) under normoxic (black) or hypoxic (red) conditions. **(b):** Phosphorescence spectrum of the reaction mixture generated in the hypoxic metabolism of **Ir5** by NADPH and rat liver microsomes. (red line, with emission maxima at 580 nm). **(c)-(e):** ESI-MS analysis of the reaction mixture generated by **Ir5**; **(c):** ESI-MS spectrum of **Ir5**. **(d):** ESI-MS spectrum of the product of **Ir5** after incubation with rat liver microsomes (0.25 mg protein/mL) and 50 μM NADPH at 37 °C for 30 min. **(e):** ESI-MS spectrum of the reduced product of **Ir5** and the simulation of **IrNH_2_1**.

**
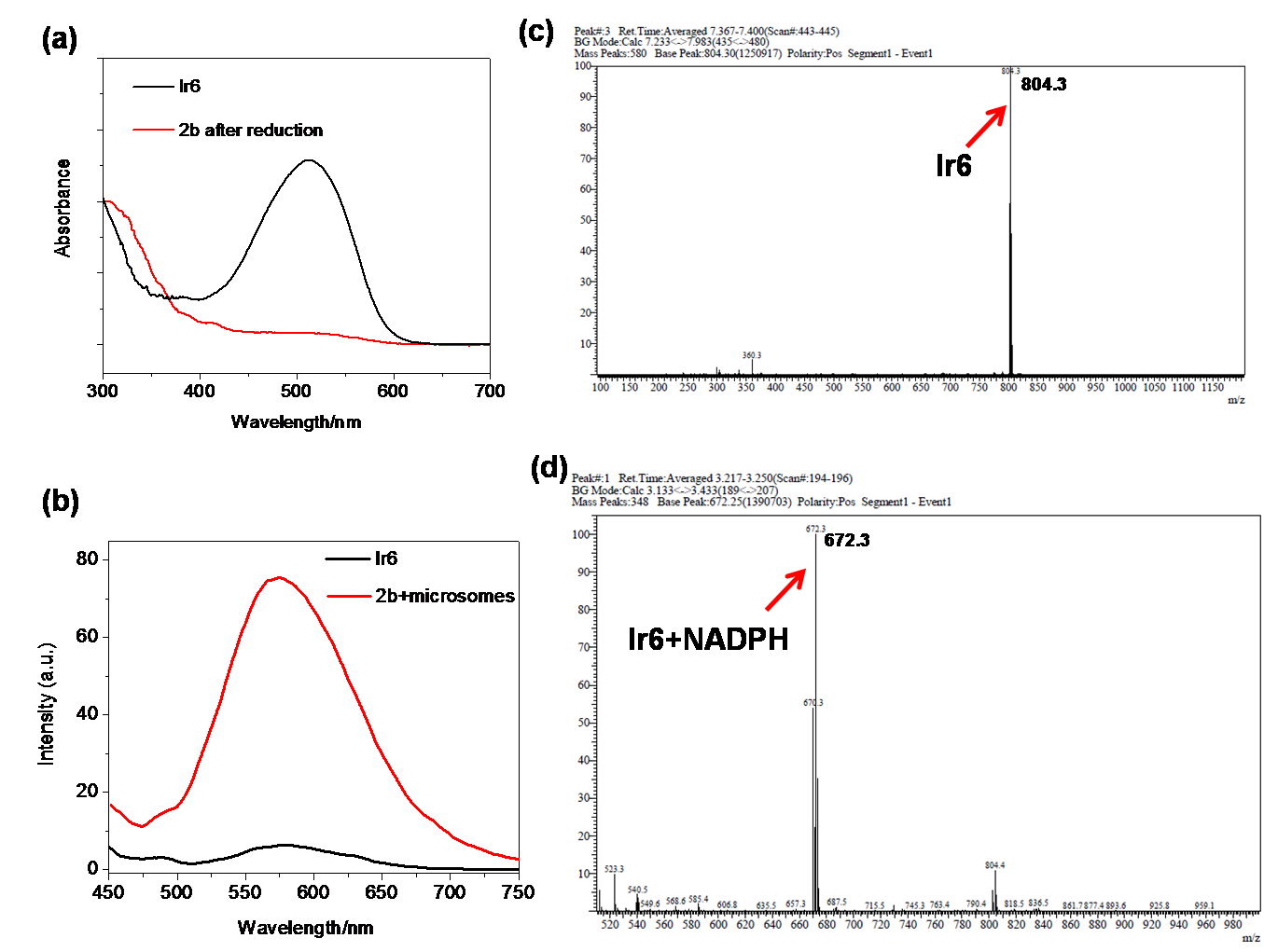

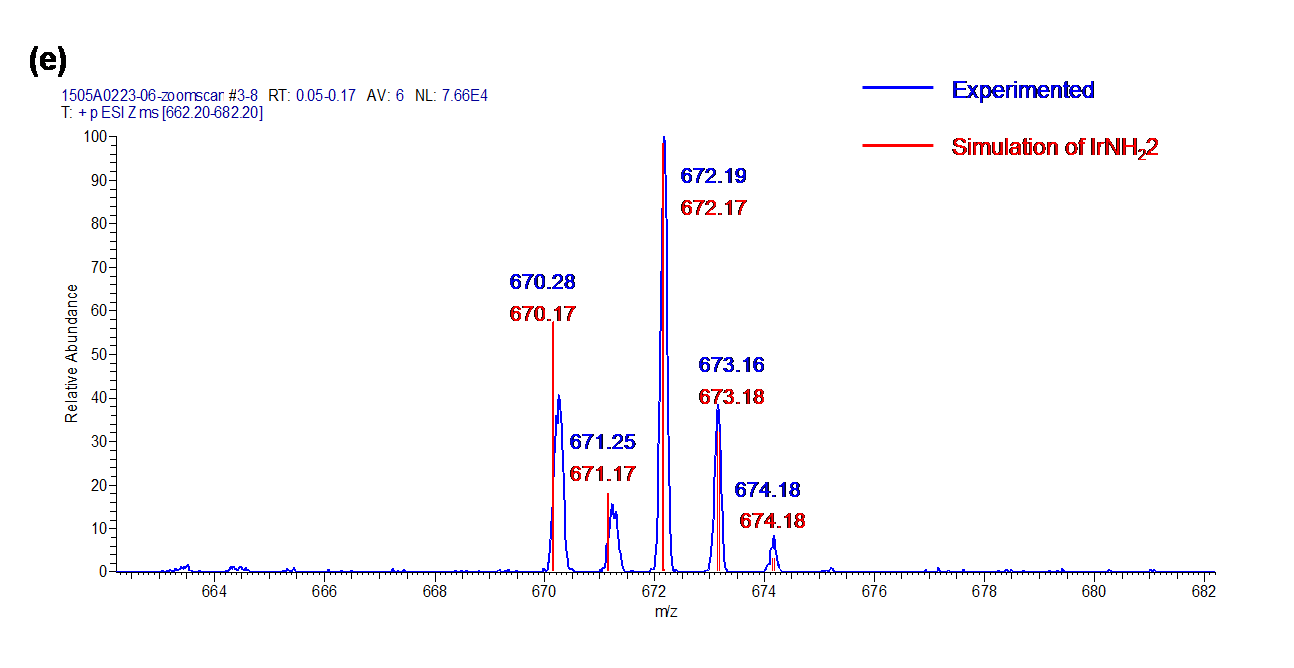
Figure S33. (a):** Spectral changes of **Ir6** in the presence of rat liver microsomes (0.25 mg protein/mL) under normoxic (black) or hypoxic (red) conditions. **(b):** Phosphorescence spectrum of the reaction mixture generated in the hypoxic metabolism of **Ir6** by NADPH and rat liver microsomes. (red line, with emission maxima at 580 nm). **(c)-(e):** ESI-MS analysis of the reaction mixture generated by **Ir6**; **(c):** ESI-MS spectrum of **Ir6**. **(d):** ESI-MS spectrum of the product of **Ir6** after incubation with rat liver microsomes (0.25 mg protein/mL) and 50 μM NADPH at 37 °C for 30 min.**(e):** ESI-MS spectrum of the reduced product of **Ir6** and the simulation of **IrNH_2_2**.


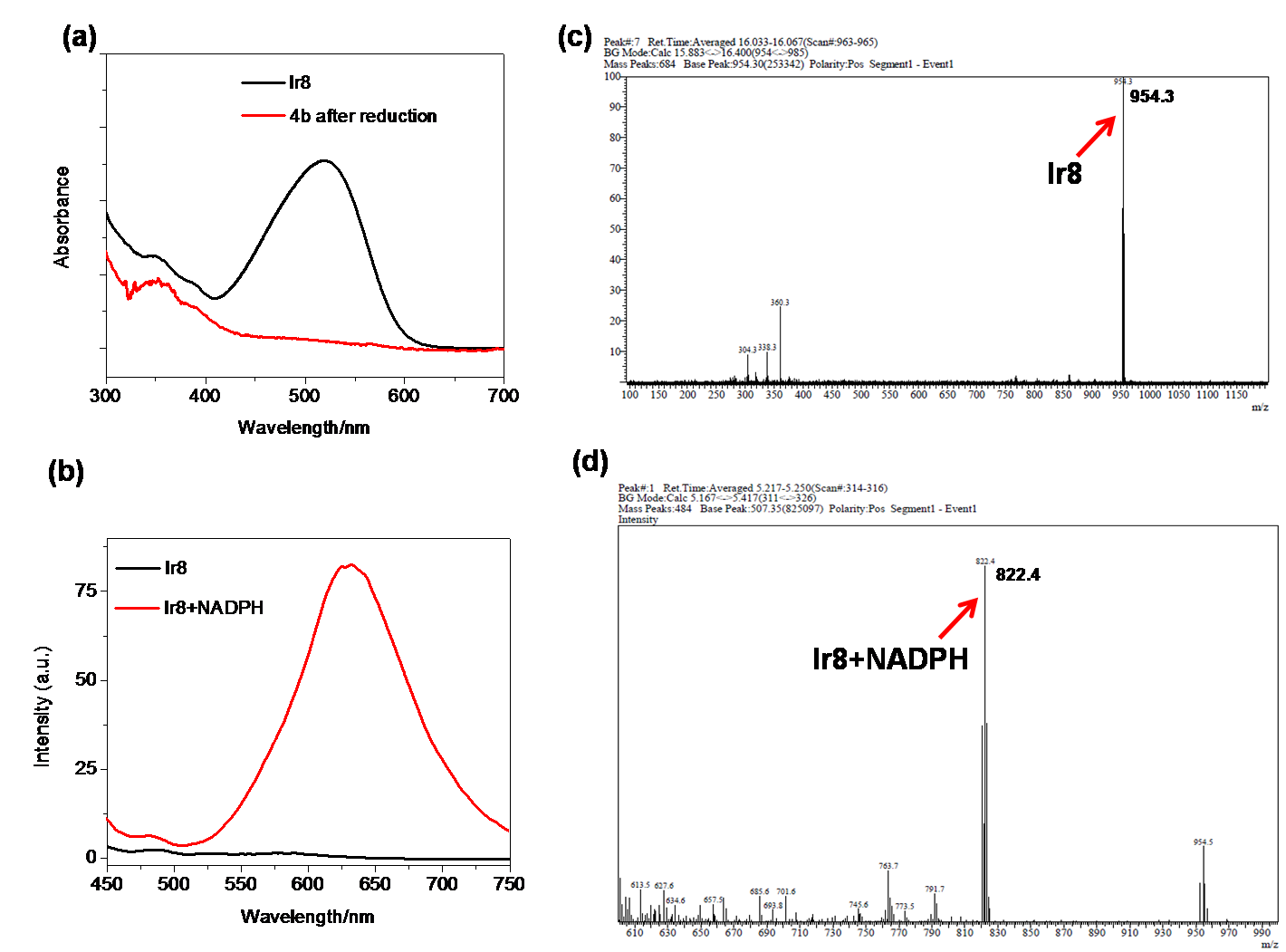

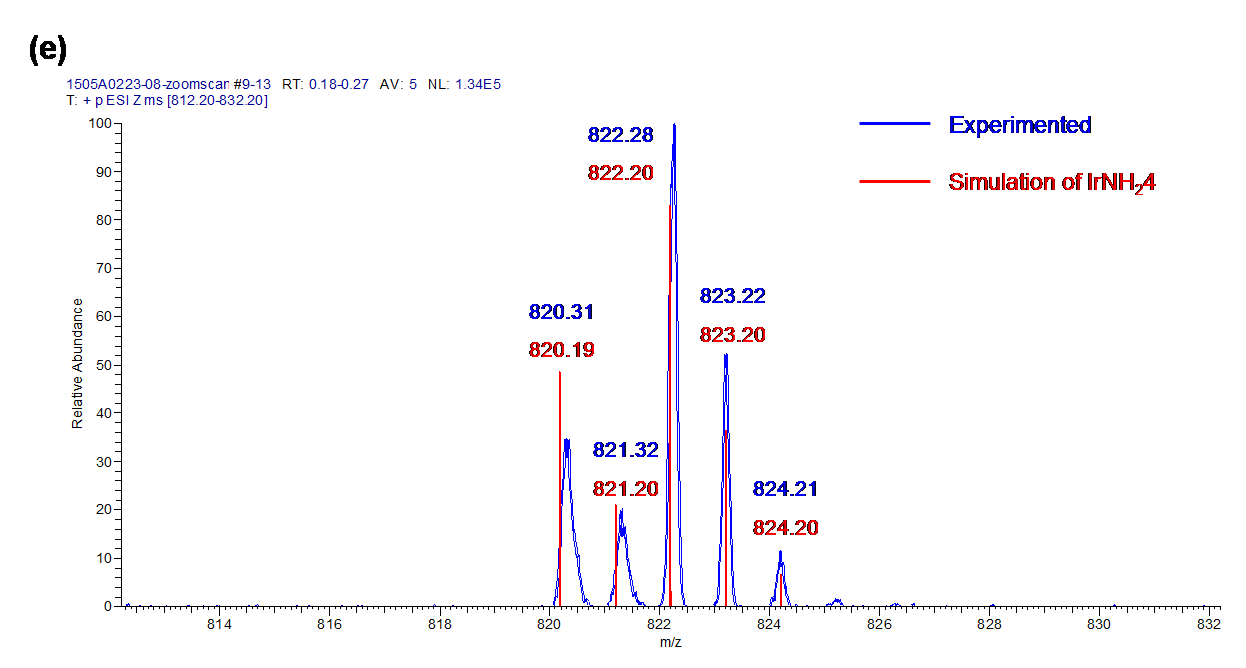
**Figure S34. (a):** Spectral changes of **Ir8** in the presence of rat liver microsomes (0.25 mg protein/mL) under normoxic (black) or hypoxic (red) conditions. **(b):** Phosphorescence spectrum of the reaction mixture generated in the hypoxic metabolism of **Ir8** by NADPH and rat liver microsomes. (red line, with emission maxima at 580 nm). **(c)-(e):** ESI-MS analysis of the reaction mixture generated by **Ir8**; **(c):** ESI-MS spectrum of **Ir8**. **(d):** ESI-MS spectrum of the product of **Ir8** after incubation with rat liver microsomes (0.25 mg protein/mL) and 50 μM NADPH at 37 °C for 30 min. **(e):** ESI-MS spectrum of the reduced product of **Ir8** and the simulation of **IrNH_2_4**.


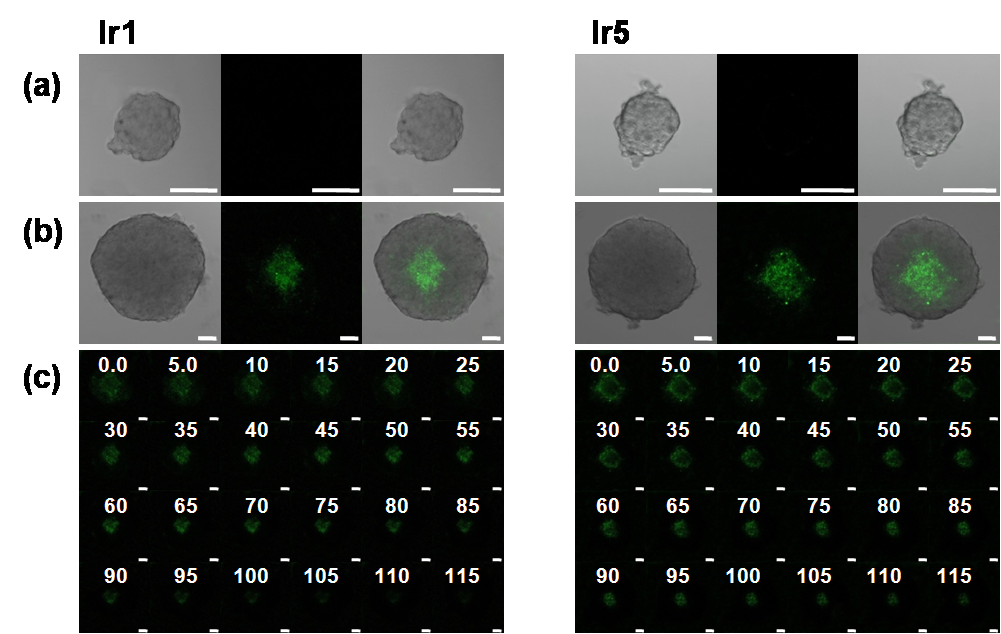
**Figure S35. (a)-(c):** Confocal images of 3D multicellular spheroids incubated with **Ir1** (Left) and **Ir5** (Right). λ_ex/em_ = 405/510 ± 20 nm; **(a):** small 3D spheroids (diameter of 100-150 μm); **(b):** large 3D spheroids (diameter of 400-500 μm); **(c):** Scanned Z-axis for large 3D spheroids at 5 μm intervals. The images were taken under 10×objective. Scale bar: 100 μm.


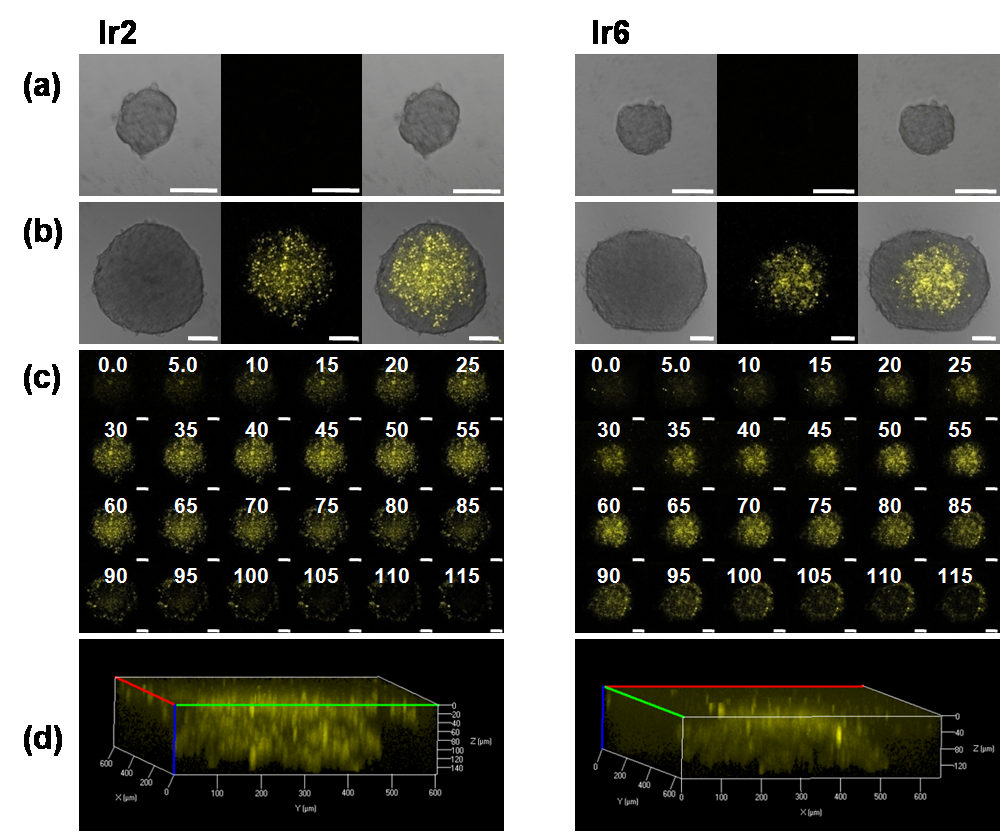
**Figure S36. (a)-(d):** Confocal images of 3D multicellular spheroids incubated with **Ir2** (Left) and **Ir6** (Right). λ_ex/em_ = 405/570 ± 20 nm; **(a):** small 3D spheroids (diameter of 100-150 μm); **(b):** large 3D spheroids (diameter of 400 - 500 μm); **(c):** Scanned Z-axis for large 3D spheroids at 5 μm intervals. **(d):** The merged images of Z-stack images of an intact spheroid. The images were taken under 10×objective. Scale bar: 100 μm.


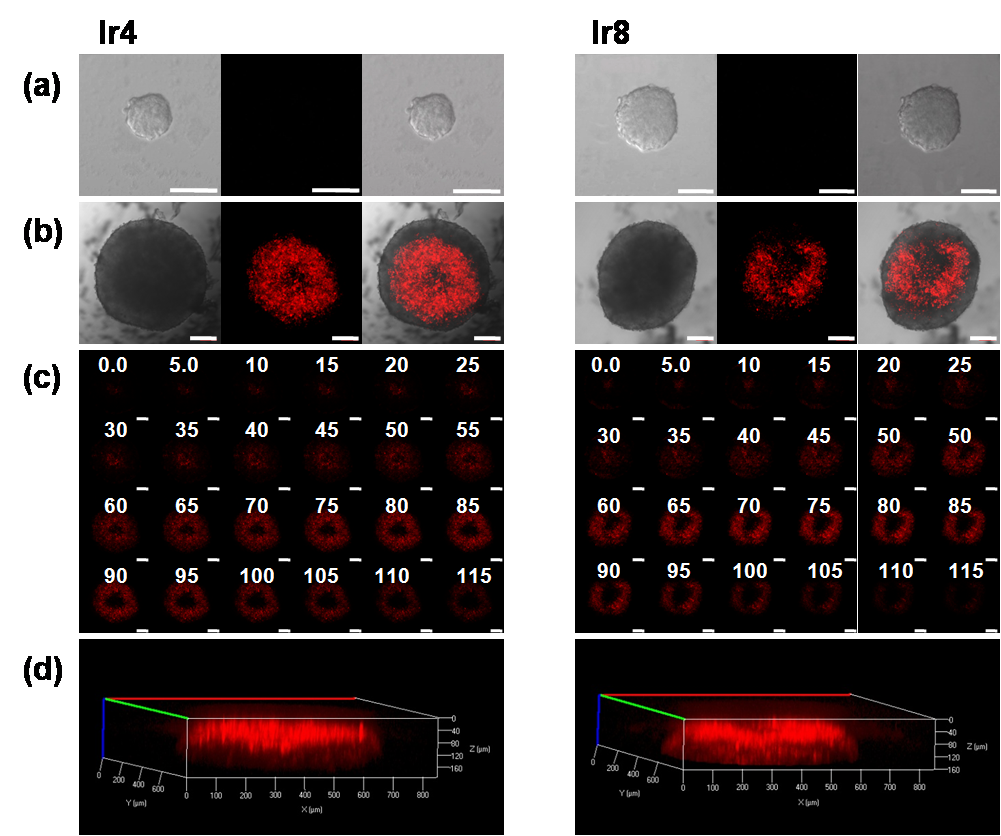
**Figure S37. (a)-(d):** Confocal images of 3D multicellular spheroids incubated with **Ir4** (Left) and **Ir8** (Right). λ_ex/em_ = 405/510 ± 20 nm; **(a):** small 3D spheroids (diameter of 100-150 μm); **(b):** large 3D spheroids (diameter of 400-500 μm); **(c):** Scanned Z-axis for large 3D spheroids at 5 μm intervals. **(d):** The merged images of Z-stack images of an intact spheroid. The images were taken under 10×objective. Scale bar: 100 μm.
